# The ABHD10-ACSL4 axis promotes pancreatic ductal adenocarcinoma growth via suppressing ferroptosis

**DOI:** 10.1186/s13046-026-03810-7

**Published:** 2026-08-12

**Authors:** Qiuying Li, Dadi Peng, Lingjun Kong, Yihua Wang, Jinmin Xue, Qiang Liu, Denghui Wang, Qi Li, Yunhai Luo, Zhengli Tan, Li Lei, Rui Liao, Hongfan Liao, Yanyao Liu, Zhongjun Wu

**Affiliations:** 1 Department of Hepatobiliary Surgery, The First Affiliated Hospital of Chongqing Medical University, Chongqing, 400016 China; 2 Natural Drug Intervention in Aging Key Laboratory of Chongqing Education Commission of China, Chongqing Three Gorges Medical College, Wanzhou, 404120 China; 3 Department of Gastrointestinal Surgery, The People’s Hospital of Yubei District of Chongqing, Chongqing, 401120 China; 4 Department of Hepatobiliary Surgery, the Affiliated Hospital of Guizhou Medical University, Guiyang, Guizhou 550001 China; 5 Department of Oncology, Key Laboratory of Immunity, Inflammation & Cancer (Chongqing Municipal Health Commission), The First Affiliated Hospital of Chongqing Medical University, Chongqing, 400016 China; 6 Department of Cell and Molecular Biology, Karolinska Institute, Stockholm, Sweden; 7 Department of Radiology, The First Affiliated Hospital of Chongqing Medical University, Chongqing, 400016 China

**Keywords:** PDAC, Ferroptosis, ABHD10, ACSL4, Protein palmitoylation

## Abstract

**Background:**

Pancreatic ductal adenocarcinoma (PDAC) is a highly aggressive malignancy with poor prognosis and limited treatment options. Ferroptosis has emerged as a potential therapeutic vulnerability in PDAC, but the upstream mechanisms regulating this process remain unclear. This study aimed to identify compounds with therapeutic potential against PDAC, determine their molecular targets, and elucidate how alpha/beta hydrolase domain-containing protein 10 (ABHD10) regulates tumor growth.

**Methods:**

A high-throughput compound screen against PDAC cell proliferation was performed, followed by limited proteolysis–mass spectrometry to identify the molecular target of halofuginone (HF). Target engagement was validated by surface plasmon resonance, cellular thermal shift assay, and drug affinity responsive target stability assay. Gain- and loss-of-function studies were conducted in PDAC cell lines and xenograft mouse models. Protein interaction, palmitoylation, and ferroptosis-related changes were evaluated using co-immunoprecipitation, acyl-biotinyl exchange, Click-iT labeling, transmission electron microscopy, and biochemical assays. Statistical analyses included Student’s t tests and one-way or two-way analysis of variance, as appropriate.

**Results:**

HF significantly suppressed PDAC growth in vitro and in vivo. ABHD10 was identified as a direct binding target of HF and was upregulated in pancreatic cancer tissues and cell lines. Functional studies showed that ABHD10 promoted cell proliferation, survival, and tumor progression. Mechanistically, ABHD10 interacted with acyl-CoA synthetase long-chain family member 4 (ACSL4) and depalmitoylated it at cysteine 157, thereby suppressing ferroptosis. HF treatment or ABHD10 depletion increased ACSL4 palmitoylation, enhanced lipid peroxidation, disrupted redox homeostasis, and induced ferroptotic changes. Depletion of ACSL4 partially reversed these effects.

**Conclusion:**

The ABHD10–ACSL4 axis promotes PDAC growth by suppressing ferroptosis. Targeting this pathway may represent a promising therapeutic strategy for PDAC.

**Supplementary Information:**

The online version contains supplementary material available at https://doi.org/10.1186/s13046-026-03810-7.

## Background

Pancreatic ductal adenocarcinoma (PDAC) remains a highly aggressive and deadly cancer. This disease is marked by rapid progression and early metastasis, and its 5-year survival rate is unsatisfactory [1–5]. The adverse outcome of PDAC is primarily caused by late diagnosis, the highly aggressive nature of the disease, and the limited efficacy of existing treatments [6–9]. The therapeutic limitation of PDAC highlights the pressing need to better understand the mechanisms underlying disease progression and uncover new therapeutic vulnerabilities.

Protein activity is frequently regulated by post-translational modifications [10–12]. Among these, reversible lipid modification such as S-palmitoylation is a layer of regulation that remains largely unexplored in PDAC. S-palmitoylation reversibly attaches a palmitoyl group to cysteine residues of proteins, thereby influencing protein localization, stability, and interactions with other molecules [13–15]. Accordingly, enzymes that add or remove palmitates may offer druggable targets for therapeutic intervention. Notably, because palmitoylation directly impacts membrane lipid composition and dynamics, it has been shown to intersect with ferroptosis [16, 17]. Ferroptosis, an iron-dependent, lipid peroxidation-driven mode of programmed cell death, has emerged as a potential therapeutic target in cancer [18, 19]. Unlike apoptosis, the unique metabolic characteristics of ferroptosis provide a novel conceptual strategy for dealing with treatment-resistant malignancies such as PDAC [20–22]. Although several drugs and natural compounds induce ferroptosis by increasing intracellular iron levels, promoting lipid peroxidation, and impairing antioxidant defenses [23–28], effectively inducing ferroptosis to inhibit tumor growth in PDAC remains a significant challenge. Given the emerging links between palmitoylation and ferroptosis, we hypothesized that palmitoylation plays a critical role in ferroptosis sensitivity and that a depalmitoylase could act as a central regulator of this process by regulating the palmitoylation dynamics of key ferroptosis-related proteins.

To test this hypothesis, we employed a PDAC cell proliferation-based unbiased compound screening approach and identified halofuginone (HF) as a chemical probe to facilitate mechanistic investigation [29–37]. This screen led to the identification of alpha/beta hydrolase domain-containing protein 10 (ABHD10) as a direct target of this compound. ABHD10 is a mitochondrial member of the ABHD protein family characterized by a conserved α/β-hydrolase fold [38]. Although ABHD10 has been identified as a depalmitoylase that removes palmitoyl groups from S-palmitoylated proteins [39, 40], its role in cancer, including PDAC, remains uncharacterized. Given that dynamic palmitoylation regulates multiple oncogenic pathways, including PI3K–AKT–mTOR, EGFR, and STAT3 signaling [41–43], these observations suggest that depalmitoylation-dependent signaling may represent an underappreciated therapeutic vulnerability in PDAC.

In this study, we show that ABHD10 functions as a constitutive suppressor of ferroptosis in PDAC by depalmitoylating the key ferroptosis executor acyl-CoA synthetase long-chain family member 4 (ACSL4) at residue C157. HF binds ABHD10 and functionally interferes with ABHD10-dependent ACSL4 depalmitoylation in cells, leading to enhanced ACSL4 palmitoylation, lipid peroxidation, and ACSL4-dependent ferroptosis. Our findings delineate a novel ABHD10‑ACSL4‑ferroptosis axis that critically regulates PDAC cell survival and establish a mechanism‑based strategy for therapeutic intervention by targeting this axis.

## Methods

### Clinical specimens

Following the acquisition of informed consent, ten paired samples of pancreatic tumor tissue and corresponding adjacent non-tumorous tissue were collected from patients undergoing pancreatectomy at the First Affiliated Hospital of Chongqing Medical University. The cohort included PDAC as well as other pancreatic tumor subtypes. The clinicopathological features of the patients are presented in Table S1.

### Animal experiments

All procedures involving animals were approved by the Laboratory Animal Ethics Committee of Chongqing Medical University (approval no. IACUC-CQMU-2025-01071). Four- to five-week-old male BALB/c nude mice were obtained from Cyagen Biosciences, Inc. (CA, USA). Male mice were used throughout the xenograft tumor experiments to maintain a consistent host background and avoid introducing sex as an additional biological variable, as the present study was not designed to assess sex-dependent differences. Mice were maintained in a specific pathogen-free environment at 20–25 °C and 50% relative humidity under a 12 h light/dark cycle, with no more than five mice per cage.

#### Xenograft tumor models and drug efficacy assessment

To establish subcutaneous tumor xenografts, PANC-1 or AsPC-1 human pancreatic cancer cells (5 × 10^6^ cells per mouse) suspended in phosphate-buffered saline (PBS; Cat: PB180327, Procell) were injected into the right flank of the nude mice. After one week, these animals were divided at random into two groups (*n* = 5) and received intraperitoneal injections of either 0.75 mg/kg HF (Cat: S8144, Selleck) or vehicle containing the same final concentration of dimethyl sulfoxide (DMSO) every three days for two weeks. This dosing regimen was selected based on previously reported in vivo HF administration protocols [44] and was not intended to represent the maximum tolerated dose (MTD).

#### Genetic manipulation and rescue experiments

For genetic intervention studies, animals were randomly allocated to experimental groups (five mice per group) and inoculated subcutaneously with PANC-1 or AsPC-1 cells stably transduced with the following constructs:

- Figure 3C–J (ABHD10 Loss-of-Function): Lv-shNC (control) and ABHD10-knockdown (Lv-shABHD10) cells.
- Figure 4C–J (ABHD10 Gain-of-Function): Lv-NC (control) and ABHD10-overexpression (Lv-ABHD10) cells.
- Figure 7G–J (Rescue Experiment 1): Lv-shNC (control), Lv-shABHD10, ACSL4-knockdown (Lv-shACSL4), or ABHD10/ACSL4 double-knockdown (Lv-shABHD10 + Lv-shACSL4) cells.
- Figure S10A–D (Rescue Experiment 2):Mice inoculated with Lv-shNC or Lv-shACSL4 PANC-1 cells were further divided to receive treatment with either HF (0.75 mg/kg) or DMSO as described in Sect. 2.2.1.

#### Tumor monitoring

Measurements of tumor dimensions were taken with a caliper every three days to determine width (a) and length (b). Tumor volume was derived from the formula: Volume = (π/6) × a² × b, where ‘a’ is the shortest diameter and ‘b’ is the longest perpendicular diameter. Body weight was also assessed every three days throughout the study. The maximal tumor diameter did not exceed 15 mm, which was in compliance with the institutional ethical guidelines. At the end of the study, mice were humanely euthanized via anesthesia, after which tumor tissues were collected and weighed for subsequent analysis.

### Cell lines

Human pancreatic cancer cell lines (AsPC-1 and PANC-1), human telomerase reverse transcriptase-immortalized human pancreatic nestin-expressing cells (hTERT-HPNE), and HEK293T cells were purchased from Procell (Wuhan, China). Short tandem repeat (STR) profiling was used to verify the identity of each cell line. HEK293T and PANC-1 cells were maintained in Dulbecco’s modified Eagle’s medium (DMEM; Gibco) containing 10% fetal bovine serum (FBS; Procell) together with 1% penicillin/streptomycin (Procell), whereas AsPC-1 cells were grown in RPMI 1640 medium (Procell) containing the same supplements. Cells were maintained at 37 °C in a humidified incubator with 5% CO₂.

### Cell viability assay

Cell viability was evaluated with the Cell Counting Kit‑8 (CCK‑8; Cat: C0038, Beyotime) following the supplier’s instructions. Briefly, 5,000 cells were seeded into each well of 96-well plates and cultured overnight for attachment. Cells received various treatments for the time periods described in the corresponding figures. Afterwards, each well got 10 µL of CCK‑8 reagent, followed by incubation at 37 °C for 3 h. Optical density was then recorded at 450 nm with a microplate reader. Viability values were normalized to the untreated controls. The half‑maximal inhibitory concentration (IC_50_) values were calculated from dose-response curves generated in GraphPad Prism software (version 10.0).

### High-throughput screening (HTS)

A library of 566 purified, well‑characterized natural products and anti‑tumor compounds (Table S6) was screened for anti‑proliferative activity against PANC‑1 cells. 5,000 cells were plated into each well of 96-well plates and cultured to allow attachment before exposure to compounds from the library at a final concentration of 1 µM. Following 48 h at 37 °C, cell viability was determined by CCK-8 assay following the supplier’s recommendations. Absorbance was recorded at 450 nm using a microplate reader. Compounds exhibiting significant decrease of cell viability relative to the DMSO‑treated control group were selected for subsequent validation.

### Wound-healing assay

When cultures in 6-well plates reached approximately 90% confluency, straight wounds were created with a sterile 200-µL pipette tip. Following PBS washing, fresh serum-free medium supplemented with the specified treatments was added. For Fig. S1, cells received HF treatment at 0, 5, 50, or 100 nM for 48 h. In Figs. S4 and S6, cells stably expressing control (Lv-shNC for Fig. S4/Lv-NC for Fig. S6), ABHD10-targeting shRNA (Lv-shABHD10), or ABHD10 cDNA (Lv-ABHD10) were treated with DMSO or 50 nM HF for 48 h. Wound images were acquired immediately (0 h) and 48 h after treatment using a Nikon Eclipse Ti microscope. Migration was quantified by measuring wound width with ImageJ and reported as wound closure percentage.

### Colony formation assay

**Fig. 1:**
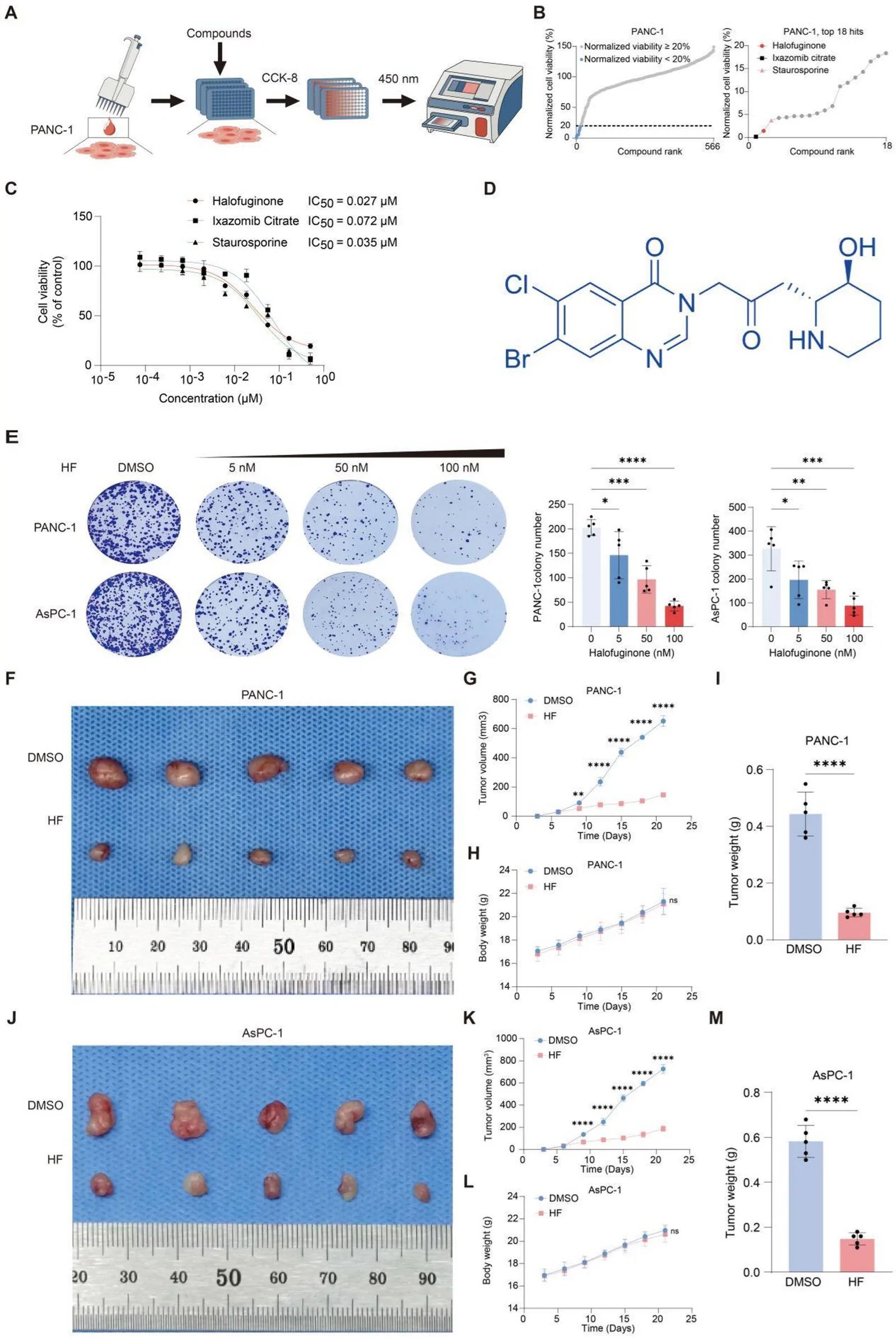
Identification and validation of HF as an inhibitor of PDAC growth. **A** Schematic diagram of the differential cytotoxicity screening workflow using a library of 566 compounds in PANC-1 cells. **B** Ranked distribution of normalized cell viability after compound treatment. The dashed line indicates the 20% viability cutoff; blue and grey dots denote compounds below and above this cutoff, respectively. The right panel shows the top 18 hits, with halofuginone, ixazomib citrate, and staurosporine highlighted. **C** Dose-response curves and calculated IC₅₀ values of HF, staurosporine, and ixazomib citrate in PANC-1 cells after 48 h treatment. IC₅₀ values were calculated by nonlinear regression. **D **Chemical structure of HF. **E** Colony formation assay of PANC-1 and AsPC-1 cells treated with the indicated concentrations of HF for 48 h, followed by culture in drug-free medium for 14 days. Representative images (left) and quantification (right) are shown. **F**–**I **Antitumor effects of HF in PANC-1 subcutaneous xenografts. Mice were treated with HF (0.75 mg/kg, intraperitoneally) or vehicle every 3 days for 2 weeks. **F** Endpoint tumor images. **G** Tumor growth curves over time. Tumor volumes were measured every 3 days. **H** Body weight curves over time. Body weights were recorded every 3 days.** I** Endpoint tumor weights. Each point represents an individual tumor. **J**–**M** Antitumor effects of HF in AsPC-1 subcutaneous xenografts. Mice were treated with HF (0.75 mg/kg, intraperitoneally) or vehicle every 3 days for 2 weeks. **J** Endpoint tumor images. **K** Tumor growth curves over time. Tumor volumes were measured every 3 days. **L** Body weight curves over time. Body weights were recorded every 3 days. **M** Endpoint tumor weights. Each point represents an individual tumor. Data are presented as mean ± SEM. Statistical analyses were performed using unpaired two-tailed Student’s t-test, one-way ANOVA, or two-way ANOVA, as appropriate. *p < 0.05; **p < 0.01; ***p < 0.001; ****p < 0.0001; ns, not significant

PANC-1 or AsPC-1 cells were inoculated into 6-well plates at 400–600 cells per well. Once attached, the cells were exposed to HF (0, 5, 50, or 100 nM; Fig. 1) for 48 h. For cells stably expressing control (Lv-shNC/Lv-NC), shABHD10, or ABHD10 cDNA, they received either DMSO or 50 nM HF (Figs. 3 and 4). The culture medium was subsequently changed to fresh complete medium and refreshed every 3–4 days. Following 14 days of culture, colonies were fixed in 4% paraformaldehyde (PFA; Cat: BL539A, Biosharp) for half an hour and stained using crystal violet solution (Cat: C0121, Beyotime) for 20 min. After gentle PBS washing and air‑drying, colonies were counted using ImageJ software.

### Transwell invasion assay

To assess invasive capacity, cells suspended in medium without serum were added onto Transwell inserts pre-coated with Matrigel (Cat: C0372, Beyotime), and treatments were added simultaneously. For Fig. S1, cells were treated with HF (0, 5, 50, or 100 nM). For Figs. S4 and S6, cells stably expressing control (Lv-shNC/Lv-NC), shABHD10, or ABHD10 cDNA were treated with DMSO or 50 nM HF. The bottom wells were supplemented with complete medium containing 20% FBS. Following a 24‑hour incubation, nonmigrated cells on the membrane’s upper side were wiped off, whereas those that had crossed to the opposite side were fixed with 4% PFA, stained with crystal violet, visualized using a Nikon Eclipse Ti microscope, and analyzed by ImageJ.

### Western blot (WB) analysis

Cell- and tissue-derived protein lysates were prepared on ice using Western and IP Cell Lysis Buffer (Cat: P0013, Beyotime). Protein levels were measured using a bicinchoninic acid protein assay (BCA; Cat: P0010S, Beyotime). Equivalent amounts of protein were resolved on 10% sodium dodecyl sulfate (SDS)-polyacrylamide gels and then electrotransferred to polyvinylidene difluoride (PVDF) membranes. After blocking with QuickBlock™ Western blocking solution (Cat: P0252, Beyotime), the blots were then exposed to the indicated primary antibodies at 4 °C overnight. Following three washes with Tris-buffered saline containing 0.1% Tween 20 (TBST; Cat: G0004, Servicebio), the blots were exposed to horseradish peroxidase (HRP)-conjugated secondary antibodies for one hour at ambient temperature. An enhanced chemiluminescence (ECL) method was used to visualize protein bands, followed by quantification with Fiji software (version 1.53c). Antibody information is summarized in Table S2.

### Immunofluorescence (IF)

Cells grown on coverslips were fixed in 4% PFA at ambient temperature for 10 min. The specimens were subsequently permeabilized with Triton X‑100 (Cat: P0096, Beyotime) for 15 min, and blocked in immunostaining blocking buffer (Cat: P0260, Beyotime) for 15 min. The cells were incubated with primary antibodies overnight at 4 °C, washed with TBST, and then exposed to fluorophore‑labeled secondary antibodies for 1 h at 37 °C with light excluded. Nuclear staining was performed with 4′,6‑diamidino‑2‑phenylindole (DAPI; Cat: P0131, Beyotime) for 8 min. Fluorescence images were captured using a laser confocal microscope. Information on the antibodies is provided in Table S2.

### Limited proteolysis-mass spectrometry (LiP-MS) analysis

LiP-MS is a chemical proteomics approach used to identify potential protein targets of small molecules by detecting ligand-induced changes in protease accessibility. Upon binding, a small molecule may alter the local conformation or protease sensitivity of its target protein, leading to changes in proteolytic peptide patterns that can be quantified by liquid chromatography-tandem mass spectrometry (LC-MS/MS). This enables target identification without a chemical probe.

PANC-1 cells were lysed in PBS. Protein concentration was normalized, and aliquots (100 µg protein per sample) were incubated with either 0.10 µM HF, 0.20 µM HF, or vehicle containing the same final concentration of DMSO, with three biological replicates per condition. Following compound treatment, limited proteolysis was carried out by sequential digestion with Proteinase K and trypsin. The resulting peptide mixtures were separated by liquid chromatography and analyzed by LC-MS/MS using a Thermo Fisher Orbitrap instrument operated in data-independent acquisition (DIA) mode. Drug-dependent peptide abundance changes were quantified and mapped to the corresponding proteins to identify candidate HF-responsive binding proteins.

### Drug affinity responsive target stability (DARTS) assay

DARTS is a probe-free target engagement assay based on the principle that small molecule binding can stabilize a target protein and increase its resistance to protease-mediated degradation. Lysis of PANC-1 cells was performed using Triton X-100 (Cat: R21239, Yuanye). Protein concentration was determined with an Enhanced BCA Protein Assay Kit and adjusted to 5 µg/µL. Aliquots of lysate were incubated with either DMSO (vehicle) or 50 nM HF for 1 h at ambient temperature. Subsequently, pronase E (Cat: HY-114158, MedChemExpress, MCE) was added at a series of dilutions (0, 1:100, 1:300, 1:1000, 1:3000, and 1:10000) and digestion proceeded for 30 min. Reactions were terminated by the addition of SDS loading buffer (Cat: P0015, Beyotime) and incubation at 99 °C for 5 min, after which the samples were analyzed via Western blotting. The stabilization of ABHD10 by HF was assessed by comparing its band intensity across pronase concentrations, using β-actin as a loading control.

### Cellular thermal shift assay (CETSA)

CETSA is based on ligand-induced thermal stabilization of target proteins and can assess drug–target engagement in intact cells by detecting the remaining soluble target protein after heat treatment [45]. Upon heating, ligand-bound proteins remain relatively more soluble than unbound proteins and can be detected by Western blotting.

CETSA was carried out to assess the thermal stabilization of ABHD10 by HF in intact cells. PANC‑1 cells were incubated with 50 nM HF or vehicle containing the same final concentration of DMSO for 2 h. After two washes with ice-cold PBS, PBS preheated to each indicated temperature ranging from 37 °C to 67 °C was added to separate cell samples. Each sample was maintained at the corresponding temperature for 3 min, cooled at room temperature for 3 min, and immediately snap-frozen in liquid nitrogen. The frozen samples were freeze-thawed three times. Cell lysis was subsequently carried out on ice using Triton X‑100 lysis buffer containing protease inhibitors (Cat: B14001, Selleck). After thawing on ice, the lysates underwent centrifugation at 12,000 × g for 20 min at 4 °C. The resulting soluble fractions were harvested, combined with SDS‑PAGE loading buffer, and examined via Western blotting as described above.

### Plasmid construction and transfection

#### Plasmid constructs

All plasmids used in this study, including ABHD10-FLAG, ACSL4-HA, and the site-specific mutant ACSL4-HA(C157S), were constructed using the pcDNA3.1(+) mammalian expression vector backbone and were commercially synthesized and sequence-verified by Tsingke Biotechnology Co., Ltd. (Beijing, China). Details of plasmid construction are provided in Table S3.

#### Cell transfection

For transient transfection, PANC-1, AsPC-1, or HEK293T cells were seeded into appropriate culture vessels and transfected upon reaching 60–70% confluence with Lipofectamine 6000 Transfection Reagent (Cat: C0526, Beyotime) following the supplier’s recommendations. The cells were harvested 48 h later for downstream assays.

### Lentiviral transduction and stable cell line generation

Lentiviruses expressing short hairpin RNA (shRNA) against ABHD10 or ACSL4, or overexpression cDNA for ABHD10, along with corresponding non-targeting control (NC) sequences, were constructed and packaged by GenePharma (Shanghai, China). The ABHD10 shRNA constructs were generated using the LV2N(U6/Puro) lentiviral vector, the ABHD10 overexpression construct was generated using the LV6(EF-1α/Puro) lentiviral vector, and the ACSL4 shRNA constructs were generated using the LV-BSD-shRNA lentiviral vector. The corresponding NC lentiviruses were generated using the matching control vectors provided by GenePharma. The pancreatic cancer cell lines PANC-1 and AsPC-1 were transduced according to the protocol provided by the vendor. For establishing lines with stable expression, transduced cells were subjected to antibiotic selection: puromycin (30 µg/mL; Cat: ST551, Beyotime) was used for cells transduced with ABHD10‑related constructs, and blasticidin S (2 µg/mL; Cat: ST018, Beyotime) was applied for ACSL4 knockdown constructs. Selection was maintained for at least 7 days until all cells in the non‑transduced control group died. The effectiveness of gene knockdown or overexpression was verified through quantitative real-time polymerase chain reaction (qRT-PCR) and Western blot analysis. The sequences for all shRNA constructs are listed in Table S4.

### RNA isolation and qRT-PCR analysis

RNA was isolated from PANC-1 or AsPC-1 cells with TRIzol (Cat: 9108, Takara) following the supplier’s recommendations. Complementary DNA (cDNA) was then generated from the RNA samples employing the PrimeScript™ RT Reagent Kit with gDNA Eraser (Cat: RR047, Takara). qRT-PCR was performed on a CFX96 Real-Time PCR Detection System (Bio‑Rad, USA) utilizing TB Green Premix Ex Taq II (Cat: RR820, Takara). β-actin served as an endogenous reference, and transcript fold changes were derived via the 2^−ΔΔCt^ method. Table S5 contains the primer sequences.

### Immunoprecipitation (IP), and Co‑immunoprecipitation (Co‑IP)

In standard IP and Co‑IP assays, cells with or without prior transfection were lysed by Western/IP lysis buffer on ice throughout a 30‑min incubation. After clarification by centrifugation, identical quantities of protein samples were rotated gently at 4 °C overnight together with anti-Flag, anti-HA, anti-ACSL4, or rabbit immunoglobulin G (IgG). The samples were subsequently incubated with Protein A/G magnetic beads (Cat: HY-K0202, MCE) for 2 h at 4 °C. Following the washing step, the bead‑bound immune complexes were heated with 2×SDS sample buffer for 10 min at 95 °C, followed by Western blot analysis. For endogenous co-IP, anti-ACSL4 was used to immunoprecipitate endogenous ACSL4 from PANC-1 and AsPC-1 cells. Antibodies for IP/Co‑IP are detailed in Table S2.

### Molecular docking

The structures of ABHD10 (AlphaFold AF-Q9NUJ1-F1; UniProt ID: Q9NUJ1) and ACSL4 (UniProt ID: O60488) were downloaded from the AlphaFold Protein Structure Database. For ABHD10–HF docking, water molecules were removed from the ABHD10 structure, and hydrogen atoms were subsequently added. After energy minimization of the HF molecule (PubChem CID: 119210), molecular docking was performed via AutoDock Vina. A grid box was positioned around the catalytic pocket of ABHD10 for the docking calculations. The pose with the most favorable binding affinity was selected as the optimal docking model. Protein–protein docking of ABHD10 with ACSL4 was conducted via the HDOCK Server. The docking score served as the main selection criterion, and the best model was selected from the 10 generated docking conformations. The MM/GBSA module of the HawkDock Server was additionally used to estimate the binding free energy, and the free energy contributions of individual amino acid residues were ranked. PyMOL 2.4 was used to visualize interactions of the selected docking models.

### RNA sequencing and analysis

TRIzol was employed for total RNA extraction. RNA purity and integrity were evaluated via A260/A280 readings and RNA integrity number (RIN). Qualified RNA was used to construct paired‑end libraries (ABclonal mRNA‑seq kit), followed by sequencing on either an Illumina NovaSeq 6000 or an MGISEQ‑T7 platform. DESeq2 served for identification of differentially expressed genes. Significance was defined as |log₂ FC| > 1 plus adjusted *p* < 0.05. Bioinformatics analysis was performed using a standard pipeline by Shanghai Applied Protein Technology Co., Ltd.

### Data-independent acquisition mass spectrometry for systematic mapping palmitoyl-proteome using acyl-biotinyl exchange

Given that ABHD10 was identified as a candidate HF-binding protein by LiP-MS and previous studies have reported ABHD10 as a mitochondrial depalmitoylase [39, 40], palmitoyl-proteomics was performed to identify potential downstream S-palmitoylated substrates regulated by ABHD10.

Protein S-palmitoylation was analyzed by Oscar DIA palmitoyl-proteomics. Briefly, proteins were extracted, free thiols were blocked with NEM, and palmitoylated cysteines were enriched via acyl-biotinyl exchange (with hydroxylamine (HA+) or in a HA- control). On-bead reduction (TCEP) and alkylation (IAA) were followed by nano-LC-MS/MS analysis on a Thermo Orbitrap Astral mass spectrometer in DIA mode. Data were processed via Spectronaut with Carbamidomethyl (C) set as a fixed modification and a false discovery rate (FDR) < 1%.

### Click-iT detection of ACSL4 palmitoylation

The Click-iT assay detects ACSL4 palmitoylation using a clickable palmitate analog. Cells are metabolically labeled with 15-azido-pentadecanoic acid, which incorporates into palmitoylated proteins. The azide group then reacts with DBCO-PEG4-Biotin via click chemistry, enabling biotin-labeled proteins to be enriched with streptavidin beads and detected by Western blotting for ACSL4.

Prior to lysis, cells received a pre‑incubation with 50 µM 15‑azido‑pentadecanoic acid (Cat: HY‑151656, MCE) either in the presence or absence of 2‑bromopalmitate (2‑BP; Cat: HY‑111770, MCE). After lysate preparation, the samples were incubated for 1 h at ambient temperature with 100 µM DBCO‑PEG4‑Biotin (Cat: HY‑130809, MCE). The reaction was terminated via methanol‑chloroform precipitation, followed by pull-down with 30 µL of streptavidin magnetic beads (Cat: HY‑K0208, MCE).

### Acyl-biotinyl exchange (ABE) assay

The ABE assay detects ACSL4 S-palmitoylation by selectively labeling cysteines that were previously modified by thioester-linked palmitoyl groups. Free cysteines are first blocked with NEM, after which hydroxylamine cleaves palmitoyl-thioester bonds and the newly exposed cysteines are labeled with a thiol-reactive biotin probe.

The ABE assay was conducted following the protocol of the IP‑ABE Palmitoylation Kit For WB (Cat: AM10314, AIMSMASS). After cell lysis, proteins were immunoprecipitated with an anti‑ACSL4 antibody-conjugated beads overnight at 4 °C. Free cysteines were blocked with NEM for 30 min. Beads were subsequently divided into two aliquots: one treated with hydroxylamine (HAM) to cleave thioester‑linked palmitoyl groups (+ HAM), and the other incubated without HAM as a control (‑HAM). After cleavage, the newly exposed cysteine thiols were incubated with a thiol‑reactive biotin probe for 60 min at room temperature. Finally, the beads underwent heating within sample buffer, followed by detection of biotinylated proteins by Western blotting using streptavidin-HRP.

For the HF time-course ABE assay, PANC-1 and AsPC-1 cells were treated with 50 nM HF for 0, 15, 30, 60, 120, and 240 min before cell lysis. For the ABHD10-dependence assay, Lv-shNC and Lv-shABHD10 cells were treated with DMSO or 50 nM HF for 48 h before ABE analysis. ACSL4 palmitoylation was detected using the ABE assay described above.

### Malondialdehyde (MDA) assay

As a lipid peroxidation end product, MDA was measured to evaluate ferroptosis-associated lipid oxidative damage. The concentration of MDA from cell lysate samples was measured with a commercial MDA Assay Kit (Cat: S0131, Beyotime) following the vendor’s protocol. In brief, lysates were incubated with thiobarbituric acid (TBA), and MDA content was determined by measuring absorbance and then adjusted based on total protein levels.

### Measurement of intracellular ferrous iron

Intracellular ferrous iron (Fe^2+^) levels were determined employing the FerroOrange probe (Cat: F374, Dojindo) following the supplier’s recommendations. This probe is designed for selective fluorescence imaging of labile Fe^2+^ in live cells.

### Measurement of intracellular lipid peroxides (lipid ROS)

Quantification of intracellular lipid ROS was carried out with the peroxide-sensitive fluorescent probe Liperfluo (Cat: L248, Dojindo) following the supplier’s recommendations.

### Glutathione assay

Cellular glutathione status was evaluated using measuring reduced (GSH) and oxidized (GSSG) glutathione with a commercial assay kit (Cat: S0053, Beyotime), following the supplier’s protocol. Absorbance at 415 nm was used to determine concentrations of GSH and GSSG via standard curves. The redox balance was expressed as the GSH/GSSG ratio.

### Transmission electron microscopy (TEM)

TEM was used to assess ferroptosis-associated mitochondrial ultrastructural changes, including mitochondrial shrinkage, increased membrane density, and reduced or disrupted cristae. 2.5% glutaraldehyde (Cat: BL911A, Biosharp) was used to fix cells or tissues overnight at 4 °C. The specimens were then washed using 0.1 M phosphate buffer, post-fixed in 1% osmium tetroxide for 1–2 h to enhance membrane contrast, washed again, and then dehydrated in a graded ethanol series followed by acetone. Samples were infiltrated and embedded in epoxy resin (1:1 and 3:1 resin/acetone mixtures, then pure resin) and polymerized at 70 °C overnight. Ultrathin sections (70–90 nm) were prepared with a Leica EM UC7 ultramicrotome, double-stained with uranyl acetate and lead citrate, and observed with a JEOL JEM‑1200EX transmission electron microscope at magnifications of 1,500× and 6,000×.

### Surface plasmon resonance (SPR) analysis

SPR is a label-free technique that monitors molecular interactions in real time. Binding between an immobilized ligand and an analyte alters the local refractive index, generating sensorgrams from which kinetic parameters are derived. The molecular interactions were analyzed by SPR using a Biacore 1 K instrument (Cytiva, USA). A CM5 sensor chip (Cytiva, USA) was activated with a mixture of 1-ethyl-3-(3-dimethylaminopropyl)carbodiimide (EDC; Cytiva) and N-hydroxysuccinimide (NHS; Cytiva). Recombinant human ABHD10 protein (ligand), adjusted to a concentration of 50 µg/mL with sodium acetate buffer (pH 4.5; Cat: BR100349, Cytiva), was chemically attached to flow cell 2 via amine coupling at 10 µL/min. Flow cell 1 underwent activation and blocking steps in the absence of protein and was used as a control. Residual active esters were deactivated. For binding assays, PBS-P+ (pH 7.4; Cat: 28995084, Cytiva), with 5% DMSO (Cat: D8418, Sigma) when required for compound analysis, was used as the running buffer. Serial dilutions of analytes, including HF (Cat: HY-N1584, MCE) or recombinant ACSL4 protein (Cat: IPD-X50097, IPODIX) where indicated, were passed across the chip at a flow rate of 30 µL/min. Each injection included a 60‑second association step, after which running buffer was introduced to allow dissociation. The surface was regenerated for 5 min between consecutive runs. Global fitting of the sensograms was performed with Biacore Insight software (v6.0, Cytiva) employing a 1:1 Langmuir binding model, allowing extraction of the kinetic rate constants.

### Subcellular fractionation

PANC-1 and AsPC-1 cells expressing ACSL4-WT or ACSL4-C157S were treated with DMSO or 50 nM HF for 48 h. Membrane and cytosolic protein fractions were extracted using the Cell Membrane and Cytoplasmic Protein Extraction Kit (Cat: P0033, Beyotime) according to the manufacturer’s instructions. Equal amounts of protein from each fraction were analyzed by Western blotting. Fraction purity was verified using ATP1A1 and β-actin as membrane and cytosolic markers, respectively. ACSL4 distribution was quantified by densitometry and normalized to the corresponding fraction markers.

### Bioinformatic analysis

Public PDAC transcriptomic datasets were analyzed to evaluate ABHD10 expression, its prognostic value, and its associations with ferroptosis-related signatures. TCGA-GTEx transcriptomic data were used for tumor-normal expression comparison. Kaplan–Meier survival analysis and Cox regression were performed to assess the association between ABHD10 expression and overall survival in TCGA and independent GEO cohorts. Pathway scores for ferroptosis, ROS, oxidative stress response, fatty acid metabolism, and lipid oxidation were calculated using gene set–based enrichment analysis, and Spearman correlation analysis was used to evaluate their associations with ABHD10 expression. Additionally, Spearman correlation analysis was used to examine the correlation between ABHD10 expression and individual ferroptosis-related genes.

### Statistical analysis

All statistical analyses were carried out with GraphPad Prism (version 10.0). Differences between two paired groups were evaluated using the paired Student’s t test, whereas comparisons between two unrelated groups were assessed with the unpaired Student’s t test. For analyses involving more than two groups, one-way or two-way ANOVA was applied as appropriate. Data are presented as the mean ± standard deviation (SD). Detailed statistical information for each experiment is provided in the corresponding figure legends. A *p* value below 0.05 was considered statistically significant (*p < 0.05; **p < 0.01; ***p < 0.001; ****p < 0.0001; ns: not significant).

## Results

### Compound screening identifies HF as an inhibitor of PDAC growth

To identify chemical modulators of PDAC cell growth, we performed a differential cytotoxicity screen in PANC-1 cells using a library of 566 natural products and anti-tumor agents (Fig. 1A). The screening results were normalized to vehicle-treated controls and are presented as a ranked distribution of normalized cell viability (%) in Fig. 1B. The dashed line indicates the 20% normalized viability cutoff, and the enlarged panel shows the top 18 hits. Ixazomib citrate, halofuginone, and staurosporine were among the strongest growth-inhibitory compounds, with normalized cell viability values of 0.15%, 1.42%, and 3.73%, respectively (Fig. 1B; Table S6). The pharmacological potency of these three candidates was further assessed by nonlinear regression analysis of dose-response curves, yielding calculated IC₅₀ values of 27 nM for HF, 35 nM for staurosporine, and 72 nM for ixazomib citrate in PANC-1 cells (Fig. 1C). Because of its lowest IC₅₀, HF was selected as a candidate for subsequent mechanistic investigation.

As a naturally derived alkaloid (Fig. 1D), HF exhibited marked anti-proliferative activity in both in vitro and in vivo PDAC models. Colony formation assay showed that HF significantly suppressed the proliferative capacity of both PANC-1 and AsPC-1 cells in a dose-dependent manner (Fig. 1E). In vivo, HF treatment inhibited tumor growth, as reflected by the decrease in tumor volume over time (Figs. 1F, G, J, K) and decreased tumor weight at the endpoint compared with the vehicle control group in both xenograft models (Figs. 1I, M). Meanwhile, HF treatment did not cause significant body weight loss (Figs. 1H, L), indicating good tolerability at this dosage.

To explore whether HF affected epithelial-to-mesenchymal transition (EMT)-associated phenotypes, we analyzed a panel of EMT markers. Preliminary PCR screening highlighted consistent changes in E-cadherin, N-cadherin, and Vimentin (Fig. S1A). Western blot analysis confirmed that HF treatment increased E-cadherin expression while decreasing N-cadherin and Vimentin levels (Fig. S1B). Consistent with this shift toward a more epithelial phenotype, HF attenuated PDAC cell migration and invasion (Figs. S1C, D). These results demonstrate HF as an inhibitor of PDAC growth both in vitro and in vivo, with attenuation of EMT-like, pro-invasive phenotypes.

### LiP-MS identifies ABHD10 as a candidate HF-binding protein

**Fig. 2:**
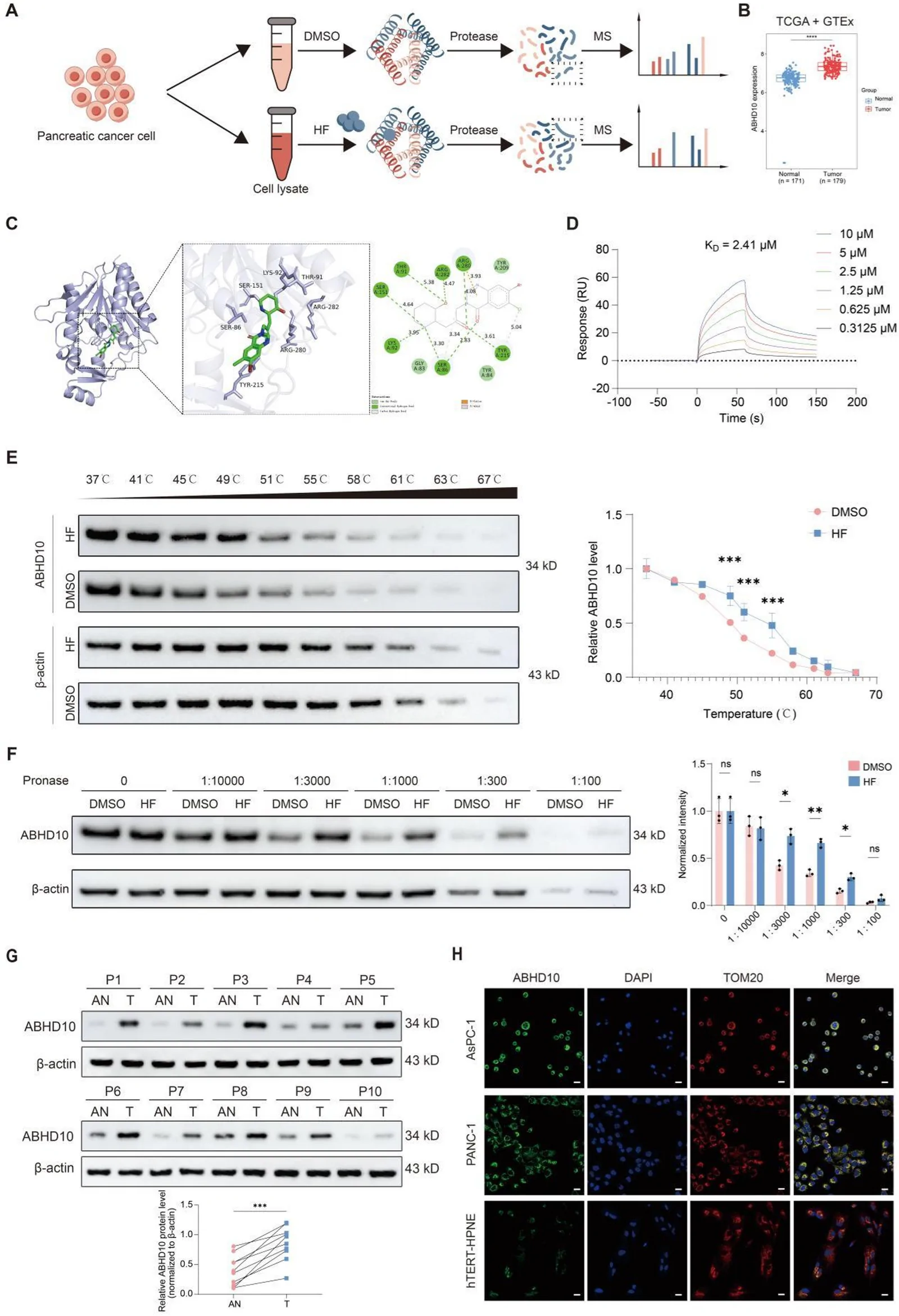
Multimodal validation identifies ABHD10 as a direct binding target of HF. **A** Schematic diagram of the LiP-MS workflow and underlying principle. PANC-1 cell lysates were incubated with HF or vehicle, followed by limited proteolysis, tryptic digestion, LC-MS/MS analysis, and DIA-based peptide quantification. Binding of HF protects specific regions of target proteins from proteolysis, resulting in increased abundance of longer peptides and decreased abundance of corresponding shorter peptides. Drug-dependent changes in proteolytic peptide abundance were then mapped to proteins to identify candidate HF-binding proteins. **B** ABHD10 expression in normal pancreatic tissues (*n* = 171) and PDAC tumor tissues (*n* = 179) based on TCGA-GTEx transcriptomic data. **C** Molecular docking model predicting the interaction between HF and ABHD10; the predicted binding poses and key interacting residues are shown. **D** SPR sensorgrams showing concentration-dependent binding of HF to immobilized recombinant ABHD10. The equilibrium dissociation constant (K_D_) is indicated. **E** CETSA. Western blot showing thermal stabilization of ABHD10 in PANC-1 cells after heat treatment with or without HF. **F** DARTS assay. Western blot showing ABHD10 protein levels in PANC-1 cell lysates after pronase treatment in the presence or absence of HF. **G** Western blot showing ABHD10 protein levels in pancreatic tumor (T) tissues and matched adjacent non-tumor (AN) tissues from patients, with quantification shown. **H** Immunofluorescence staining showing ABHD10 (green) and the mitochondrial marker TOM20 (red) in PANC-1, AsPC-1, and hTERT-HPNE cells. Nuclei were counterstained with DAPI (blue). Scale bar, 20 μm. Data in bar graphs are presented as mean ± SEM. Statistical significance was determined by Wilcoxon rank-sum test, paired two-tailed Student’s t-test or two-way ANOVA, as appropriate. *p < 0.05; **p < 0.01; ***p < 0.001; ****p < 0.0001; ns, not significant

To identify protein targets associated with HF-mediated effects, we employed LiP-MS. This method bases on the principle that compound binding can protect specific protein regions from proteolytic cleavage, resulting in increased abundance of longer peptide and decreased abundance of shorter peptides fragments (Fig. 2A). Although whole-cell lysates were used as the starting proteome, LiP-MS identifies candidate binding proteins through LC-MS/MS-based separation and DIA quantification of drug-dependent proteolytic peptide changes. By statistically analyzing these characteristic peptide patterns across different drug concentration groups and mapping them to the protein level, we identified ABHD10 as a candidate exhibiting consistent binding-related signatures (Figs. S2A, B). Analysis of TCGA-GTEx transcriptomic data showed that ABHD10 expression was significantly higher in PDAC tumor tissues than in normal pancreatic tissues (Fig. 2B). Kaplan–Meier survival analysis and Cox regression analyses across TCGA and independent GEO cohorts showed that ABHD10 expression alone was not significantly associated with overall survival (Fig. S2C–E), suggesting that ABHD10 may not serve as a robust standalone prognostic biomarker in PDAC. We evaluated the interaction between HF and ABHD10 by molecular docking, which predicted a potential binding mode between the two molecules (Fig. 2C). This interaction was then confirmed by SPR, with an equilibrium dissociation constant (K_D_) of 2.41 µM, indicating moderate‑affinity binding (Fig. 2D). Subsequently, CETSA and DARTS assays verified the interaction of HF and ABHD10 in a cellular context (Figs. 2E–F). Together, these data establish ABHD10 as a direct binding target of HF. ABHD10 protein levels were elevated in pancreatic tumor tissues relative to adjacent normal tissues (Fig.2G). Consistent with these results, ABHD10 protein levels in pancreatic cancer cell lines (PANC-1 and AsPC-1) were significantly higher than those in normal pancreatic epithelial cells (hTERT-HPNE), and ABHD10 localized to mitochondria, as indicated by co-localization with TOM20 (Fig. 2H). These findings support the potential functional relevance of ABHD10 in pancreatic cancer.

### Functional validation establishes ABHD10 as an oncogenic driver of PDAC

**Fig. 3:**
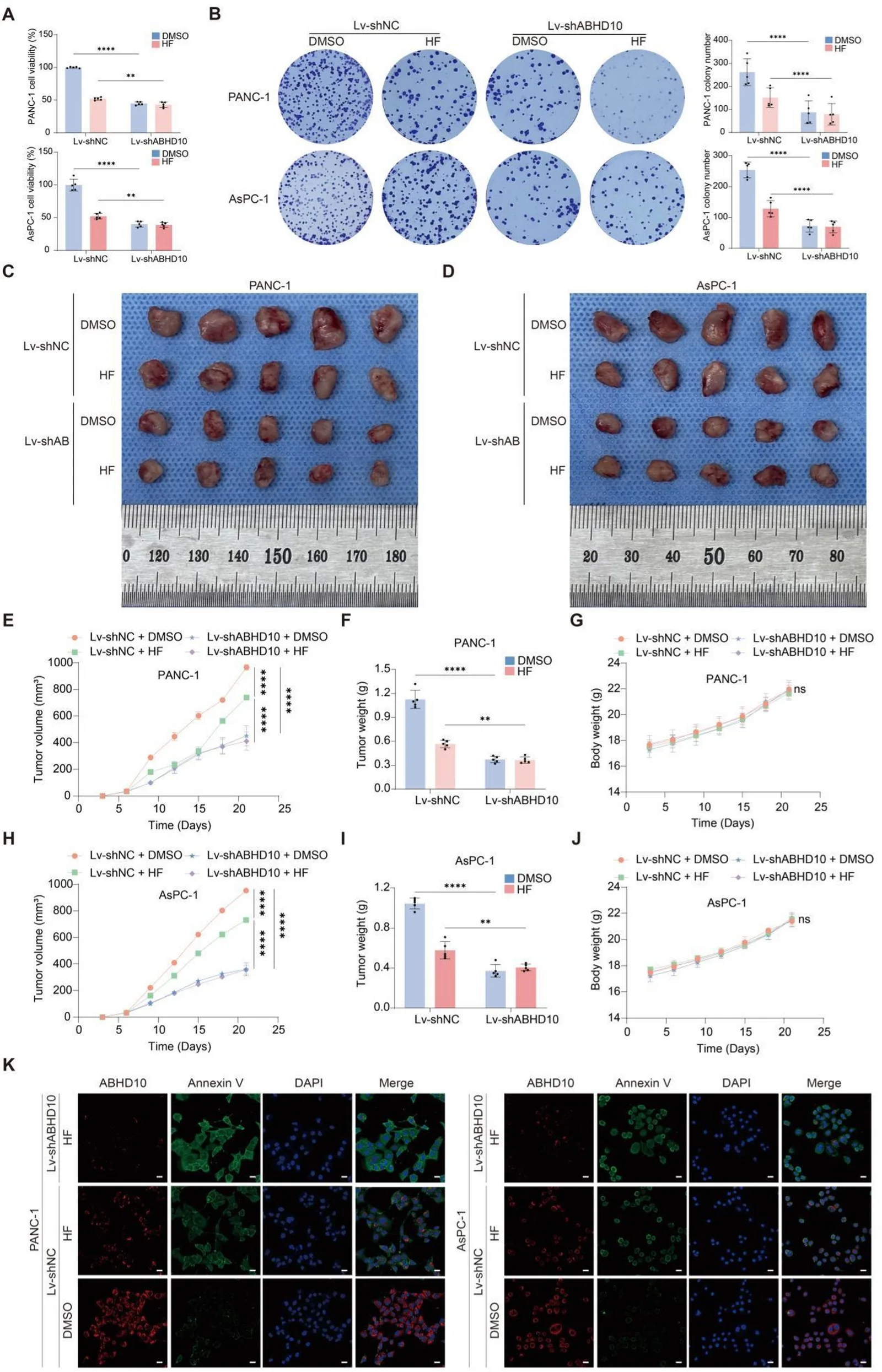
Loss-of-function validation supports ABHD10 as a driver of PDAC growth. **A **CCK-8 assays showing cell viability in PANC-1 and AsPC-1 cells with stable ABHD10 knockdown (Lv-shABHD10) or control (Lv-shNC). Cells were treated with vehicle or HF (50 nM) for 48 h. **B** Colony formation assays showing clonogenic growth of PANC-1 and AsPC-1 cells with stable ABHD10 knockdown (Lv-shABHD10) or control (Lv-shNC). Cells were treated with vehicle or HF (50 nM) for 48 h and then cultured in drug-free medium for a total of 14 days. Representative images and quantification are shown. **C**–**J** In vivo tumor growth of PANC-1 and AsPC-1 xenografts with stable ABHD10 knockdown. Mice were implanted with Lv-shNC or Lv-shABHD10 cells and treated with vehicle or HF (0.75 mg/kg, i.p.) for 2 weeks (*n* = 5 per group). **C**–**D** Representative images of tumors at the endpoint. **E**, **H** Tumor growth curves over time. **F**, **I** Tumor weights at the endpoint. Each point represents an individual tumor. **G**, **J** Body weight curves over time. **K **Immunofluorescence images showing ABHD10 (red), Annexin V (green), and nuclei (DAPI, blue) in PANC-1 and AsPC-1 cells under the indicated conditions. Merged images are shown. Scale bar, 20 μm. Data are presented as mean ± SEM. Statistical significance was determined by two-way ANOVA. *p < 0.05; **p < 0.01; ***p < 0.001; ****p < 0.0001; ns, not significant

**Fig. 4:**
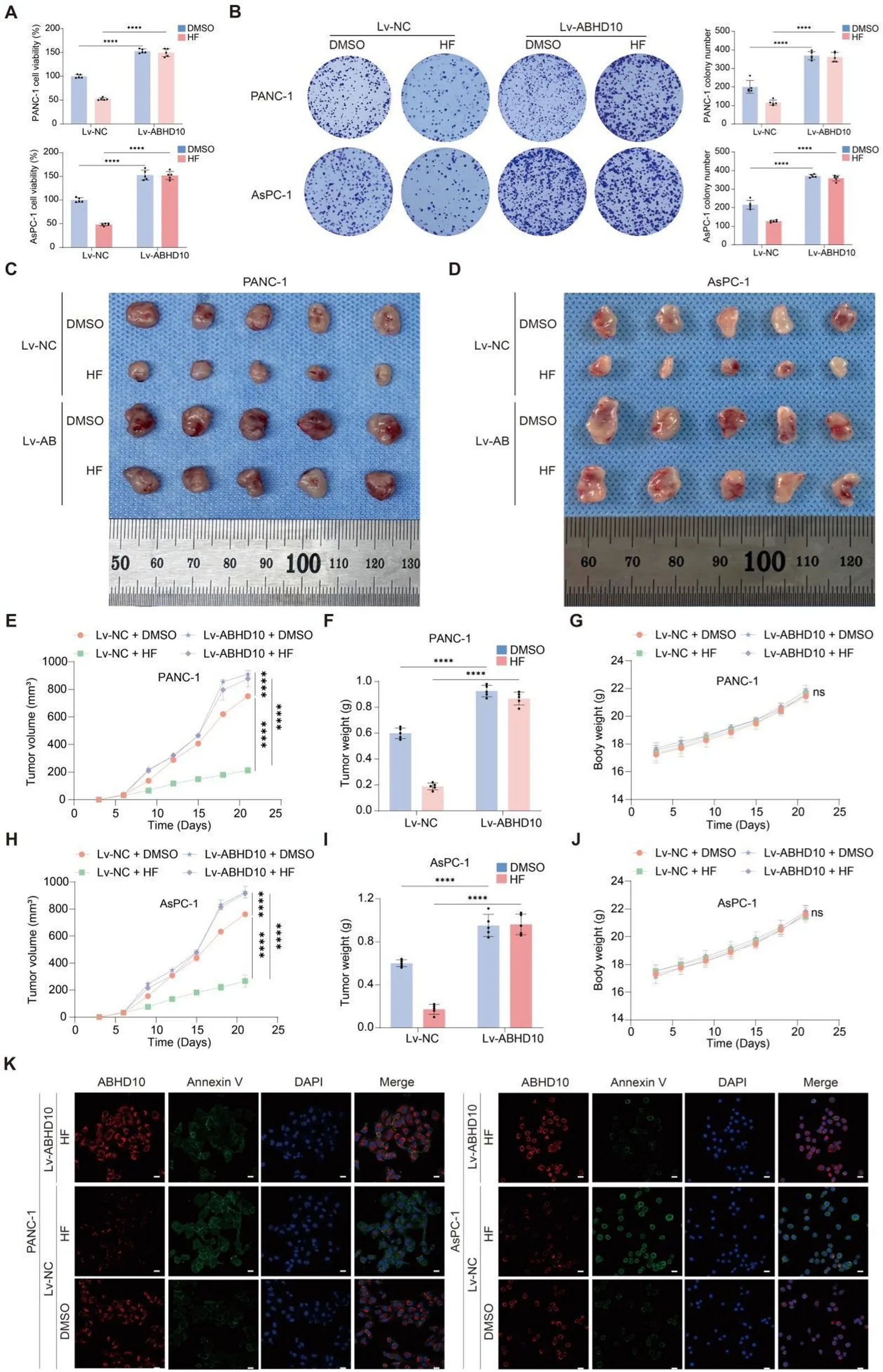
Gain-of-function validation supports ABHD10 as a promoter of PDAC growth. **A** CCK-8 assays showing cell viability in PANC-1 and AsPC-1 cells with stable ABHD10 overexpression (Lv-ABHD10) or control (Lv-NC). Cells were treated with vehicle or HF (50 nM) for 48 h. **B** Colony formation assays showing clonogenic growth of PANC-1 and AsPC-1 cells with stable ABHD10 overexpression (Lv-ABHD10) or control (Lv-NC). Cells were treated with vehicle or HF (50 nM) for 48 h and then cultured in drug-free medium for a total of 14 days. Representative images and quantification are shown. **C**–**J** In vivo tumor growth of PANC-1 and AsPC-1 xenografts with stable ABHD10 overexpression. Mice were implanted with Lv-NC or Lv-ABHD10 cells and treated with vehicle or HF (0.75 mg/kg, i.p.) for 2 weeks (*n* = 5 per group). **C**–**D** Representative images of tumors at the endpoint. **E**, **H** Tumor growth curves over time. **F**, **I** Tumor weights at the endpoint. Each point represents an individual tumor. **G**, **J** Body weight curves over time. **K** Immunofluorescence images showing ABHD10 (red), Annexin V (green), and nuclei (DAPI, blue) in PANC-1 and AsPC-1 cells under the indicated conditions. Merged images are shown. Scale bar, 20 μm. Data are presented as mean ± SEM. Statistical significance was determined by two-way ANOVA. *p < 0.05; **p < 0.01; ***p < 0.001; ****p < 0.0001; ns, not significant

To define the functional role of ABHD10 in PDAC, we established stable ABHD10‑knockdown (LOF) and ABHD10‑overexpression (GOF) cell lines in PANC‑1 and AsPC‑1 backgrounds (Figs. S3A, B; Figs. S5A, B). For subsequent interaction studies, cells were treated with 50 nM HF, approximately twice the IC₅₀ value (27 nM) determined in our initial screen, to ensure robust pharmacological activity. Functional assays consistently showed that ABHD10 positively regulated PDAC cell growth in vitro. CCK‑8 assays demonstrated that ABHD10 knockdown significantly reduced cell viability, whereas ABHD10 overexpression enhanced it (Figs. 3A and 4A). Similarly, colony formation assays showed that ABHD10 depletion suppressed proliferative capacity, whereas ABHD10 overexpression increased colony-forming ability (Figs. 3B and 4B). Notably, HF did not further affect cell viability or colony-forming ability in ABHD10-knockdown or ABHD10-overexpressing cells beyond the effects of ABHD10 manipulation alone (Figs. 3A and B and 4A and B). This growth-promoting role of ABHD10 was confirmed in vivo. In subcutaneous xenograft models, tumors formed by ABHD10-knockdown cells grew more slowly and were smaller, whereas tumors formed by ABHD10-overexpressing cells exhibited accelerated growth and greater tumor burden than those in the control group. Notably, HF treatment did not further suppress tumor growth in the ABHD10-knockdown group, nor did it significantly alter tumor growth in the ABHD10-overexpression group (Figs. 3C–F and H–I and 4C–F and H–I). Meanwhile, no significant difference in body weight was observed across groups during treatment, suggesting good overall tolerability in vivo (Figs. 3G and J and 4G and J). Together, these findings support ABHD10 as a growth-promoting factor in PDAC and as a major functional mediator of the anti-tumor effects of HF. ABHD10 modulation was also associated with changes in cell motility, EMT-like features, and apoptosis. ABHD10 knockdown reduced cell migration and invasion, increased E-cadherin, and decreased N-cadherin and Vimentin, whereas ABHD10 overexpression showed the opposite pattern (Figs. S4A–C; Figs. S6A–C). In addition, ABHD10 depletion promoted apoptosis, while ABHD10 overexpression reduced apoptotic cell death (Figs. 3K and 4K), further suggesting a growth-promoting function of ABHD10 in PDAC.

### Multi-omic profiling identifies ACSL4 as a key downstream effector of ABHD10

**Fig. 5:**
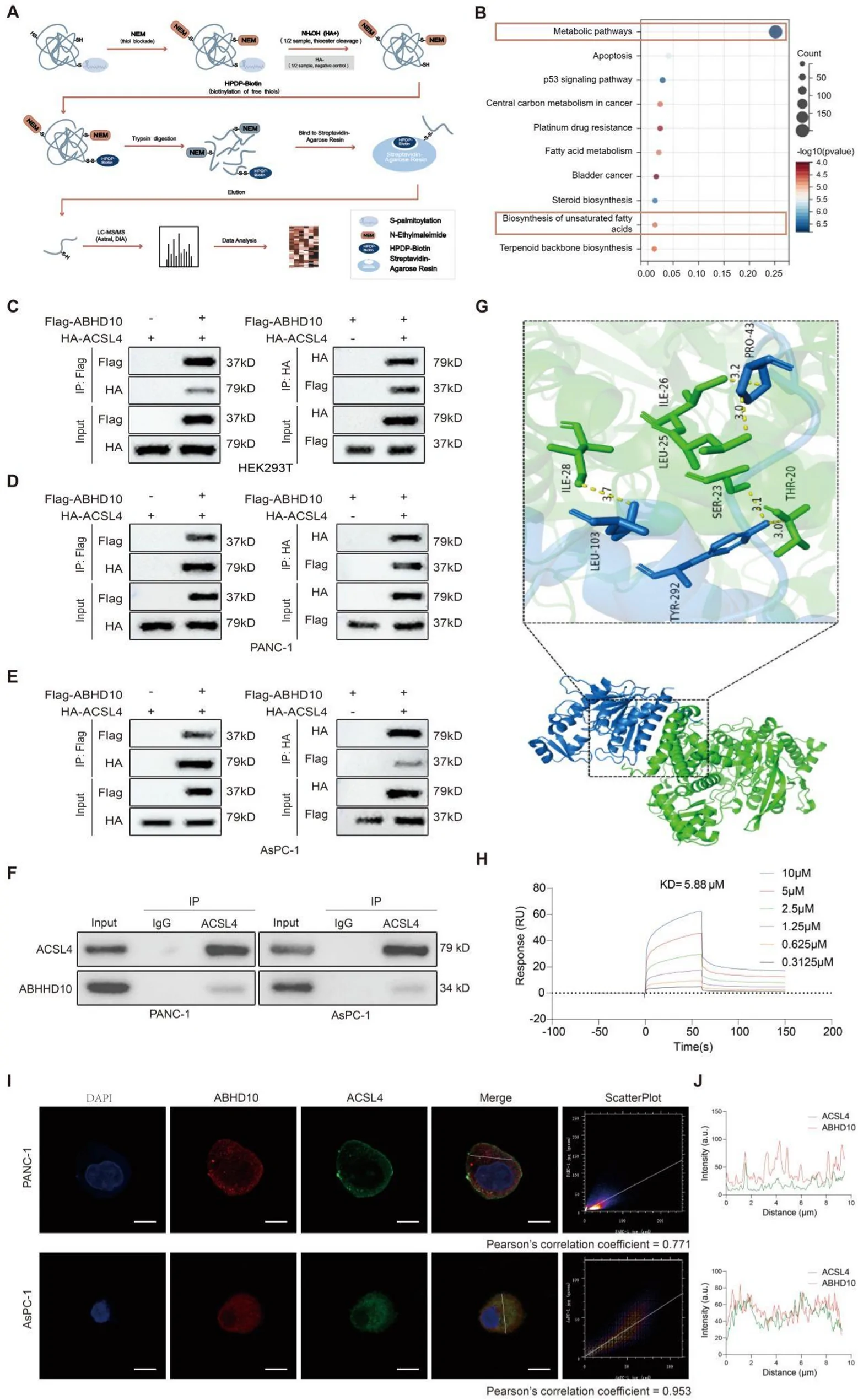
Palmitoylome analysis and biochemical validation of the ABHD10–ACSL4 interaction. **A** Workflow and schema of S-palmitoylome profiling in PANC-1 cells. **B** KEGG pathway enrichment analysis of genes downregulated upon HF treatment. The “Biosynthesis of unsaturated fatty acids” pathway and “Metabolic pathways” are highlighted. **C**–**E** Co-IP assays showing the interaction between ABHD10 and ACSL4 in HEK293T, PANC-1, and AsPC-1 cells. Cells were co-transfected with Flag-ABHD10 and HA-ACSL4 as indicated. Immunoprecipitation was performed using anti-Flag or anti-HA antibodies, followed by immunoblotting with the indicated antibodies. Input lysates are shown as controls. **F** Endogenous co-IP analysis of the ABHD10–ACSL4 interaction in PANC-1 and AsPC-1 cells. Endogenous ACSL4 was immunoprecipitated using an anti-ACSL4 antibody, followed by immunoblotting for ABHD10 and ACSL4. IgG was used as a negative control. **G** Molecular docking model showing the predicted ABHD10–ACSL4 complex, with key interacting residues and hydrogen-bond interactions highlighted. **H **SPR analysis showing direct binding between purified recombinant ABHD10 and ACSL4 proteins. The sensorgrams show concentration-dependent binding, with a K_D_ of 5.88 µM. **I**–**J** Confocal immunofluorescence analysis showing co-localization of ABHD10 and ACSL4 in pancreatic cancer cells. (**I**) Representative confocal images of PANC-1 and AsPC-1 cells stained with DAPI (blue), ABHD10 (red), and ACSL4 (green), with merged images shown. Scatter plots of pixel intensities (ABHD10 vs. ACSL4) are displayed to visualize co-localization, with Pearson’s correlation coefficients indicated. **J** Line-scan fluorescence intensity profiles across the indicated regions showing overlapping distribution of ABHD10 (red) and ACSL4 (green) signals relative to DAPI (blue), supporting their intracellular co-localization. Scale bar, 5 μm

To systematically identify the functional substrates underlying the oncogenic role of ABHD10, we performed S‑palmitoylome profiling in PANC‑1 cells (Fig. 5A). Comparative analysis revealed several proteins with significantly altered palmitoylation levels. Among these candidates, ACSL4, a key enzyme involved in fatty acid activation, was identified as a potential substrate of particular interest. To further prioritize functionally relevant candidates, we integrated the palmitoylome data with transcriptomic profiles. KEGG pathway enrichment analysis of downregulated genes following HF treatment revealed significant suppression of unsaturated fatty acid metabolism (Fig. 5B), a pathway in which ACSL4 plays a central role. These results support ACSL4 as a major candidate molecule regulated by ABHD10-mediated depalmitoylation. In public PDAC datasets, ABHD10 expression showed significant associations with WP ferroptosis, ROS pathway, fatty acid metabolism, oxidative stress response, and lipid oxidation scores, while GOBP ferroptosis showed a negative trend (Fig. S7A). In addition, ABHD10 expression correlated with multiple ferroptosis-related genes, including ACSL4 and other redox- and lipid metabolism-related regulators (Fig. S7B). These results support an association between ABHD10 and ferroptosis-related transcriptional programs in PDAC patient datasets. ACSL4 physically associated with ABHD10, as shown by reciprocal co-IP in HEK293T, PANC-1, and AsPC-1 cells co-expressing both proteins (Figs. 5C–E). Endogenous co-IP further showed that ABHD10 was detected in ACSL4 immunoprecipitates but not in IgG controls in both PANC-1 and AsPC-1 cells, supporting their endogenous association (Fig. 5F). To further characterize the molecular basis of this interaction, we carried out molecular docking simulation, which predicted a ABHD10‑ACSL4 complex. Key interaction analysis identified hotspot residues (including Pro43 and Leu103 on ABHD10) that contributed substantially to binding with ACSL4 (Fig. 5G; Table S7). SPR analysis showed concentration-dependent binding of recombinant ACSL4 to immobilized recombinant ABHD10, with a K_D_ of 5.88 µM (Fig. 5H). In addition, immunofluorescence in PANC-1 and AsPC-1 cells revealed co-localization of ABHD10 and ACSL4, predominantly within mitochondria, supporting their association within a shared subcellular compartment (Figs. 5I–J). Collectively, these multi‑omic and biochemical findings establish ACSL4 as a direct interaction partner and a key functional downstream substrate of ABHD10.

### ABHD10 depalmitoylates ACSL4 at C157 to suppress ferroptosis and promote tumorigenesis

**Fig. 6:**
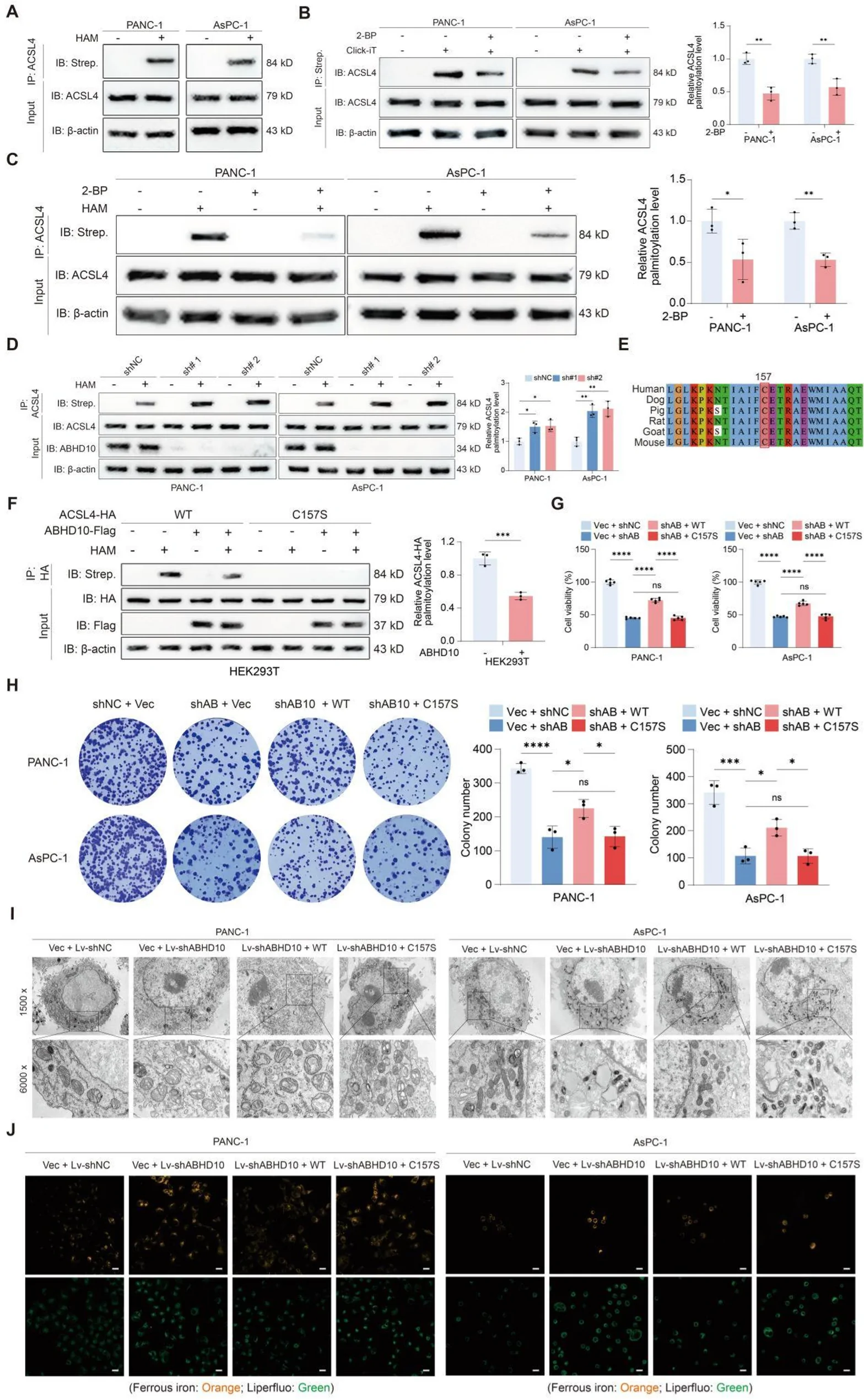
ACSL4 C157 palmitoylation is required for ABHD10-dependent suppression of ferroptosis and promotion of PDAC growth. **A** ABE assay showing ACSL4 palmitoylation in PANC-1 and AsPC-1 cells with or without hydroxylamine (HAM) treatment. **B** Click-iT palmitoylation assay showing ACSL4 palmitoylation and its inhibition by 2-BP in PANC-1 and AsPC-1 cells. **C** ABE assay showing ACSL4 palmitoylation in PANC-1 and AsPC-1 cells with or without 2-BP treatment. **D** ABE assay showing increased ACSL4 palmitoylation in PANC-1 and AsPC-1 cells with stable ABHD10 knockdown compared with Lv-shNC controls. **E** Palmitoyl-proteomic site mapping identifying Cys157 (C157) of ACSL4 as a candidate palmitoylation site. Multiple sequence alignment shows that C157 is highly conserved across species. **F** ABE assay showing ACSL4 palmitoylation in HEK293T cells transfected with plasmids encoding ABHD10-Flag together with ACSL4-HA (WT) or its C157S mutant for 48 h. **G**–**H** Functional rescue assays in ABHD10-knockdown cells (Lv-shABHD10) reconstituted with ACSL4-HA (WT) or the C157S mutant. **G** CCK-8 assays showing cell viability under the indicated conditions. **H** Colony formation assays showing clonogenic capacity; representative images and quantification are shown. **I **TEM analysis showing mitochondrial ultrastructure under the indicated conditions in ABHD10-knockdown PANC-1 cells and AsPC-1 cells reconstituted with ACSL4-HA (WT) or C157S. Scale bars, 2 μm and 500 nm. **J** Fluorescence images showing labile ferrous iron (Fe²⁺; orange) and lipid peroxidation (Liperfluo; green) in PANC-1 (left) and AsPC-1 (right) cells under the indicated conditions. Scale bar, 20 μm. Data are presented as mean ± SEM. Statistical analyses were performed using unpaired two-tailed Student’s t-test or one-way ANOVA, as appropriate. *p < 0.05; **p < 0.01; ***p < 0.001; ****p < 0.0001; ns, not significant

ABE assay and Click‑iT confirmed that ACSL4 is S‑palmitoylated in pancreatic cancer cells, a modification that was abolished by 2‑bromopalmitate (2‑BP), which broadly inhibits palmitoylation (Figs. 6A–C). Modulating ABHD10 expression influenced this modification: ABHD10 knockdown increased, whereas its overexpression decreased, ACSL4 palmitoylation levels (Fig. 6D; Fig. S8A). Concordantly, HF treatment enhanced ACSL4 palmitoylation, consistent with functional interference with ABHD10-dependent ACSL4 depalmitoylation in cells (Fig. S8B). Short-term HF treatment rapidly enhanced ACSL4 palmitoylation before obvious ABHD10 protein reduction, while total ACSL4 remained unchanged (Fig. S8C). Furthermore, HF increased ACSL4 palmitoylation in control cells but did not further elevate it in ABHD10-knockdown cells, where basal ACSL4 palmitoylation was already increased (Fig. S8D). These findings support that HF functionally suppresses ABHD10-mediated ACSL4 depalmitoylation in PDAC cells. Palmitoyl-proteomics-based site mapping following ABHD10 knockdown identified Cys157 (C157) of ACSL4 as a candidate palmitoylation site. Multiple sequence alignment showed that C157 is highly conserved across species, supporting its potential functional importance (Fig. 6E). Notably, this putative regulatory residue appeared to be spatially distinct from the primary protein-protein interaction interface predicted by molecular docking. To examine its functional relevance, we generated ACSL4‑HA wild‑type (WT) and C157S mutant constructs. ABE and Click‑iT assays in HEK293T cells revealed that the C157S mutation markedly attenuated ACSL4 palmitoylation. Moreover, co-expression of ABHD10-Flag decreased palmitoylation of ACSL4-WT, whereas it had no discernible effect on the C157S mutant, indicating that C157 is the major residue targeted by ABHD10 for depalmitoylation (Fig. 6F; Fig. S8E). Subcellular fractionation further showed that ACSL4-WT was more enriched than the palmitoylation-defective C157S mutant in the membrane fractions of both PANC-1 and AsPC-1 cells. HF increased the abundance of ACSL4-WT in the membrane fraction but had little effect on ACSL4-C157S, whereas the cytosolic fraction showed the opposite trend (Fig. S8F). These findings suggest that C157 palmitoylation promotes ACSL4 membrane association, thereby supporting its pro-ferroptotic function. We next investigated the functional requirement of C157 palmitoylation. In cells with stable ABHD10 knockdown, re‑expression of ACSL4-WT, but not the C157S mutant, restored the cell viability and clonogenic capacity that were suppressed upon ABHD10 loss (Figs. 6G–H). Strikingly, transmission electron microscopy showed mitochondrial changes related to ferroptosis in ABHD10‑deficient cells, including condensation and increased membrane density. These phenotypes were rescued by ACSL4-WT but not by the C157S mutant (Fig. 6I). At the biochemical level, re-expression of ACSL4-WT (not C157S) reversed the elevation of lipid peroxidation caused by ABHD10 knockdown (measured by MDA and Liperfluo staining), restored the reduced GSH/GSSG ratio, and shifted labile Fe²⁺ levels toward baseline (Fig. 6J; Figs. S8G–H). Taken together, these data show that ABHD10 directly depalmitoylates ACSL4 at C157, thereby limiting lipid peroxidation and ferroptosis.

### The oncogenic function of ABHD10 is dependent on ACSL4

**Fig. 7:**
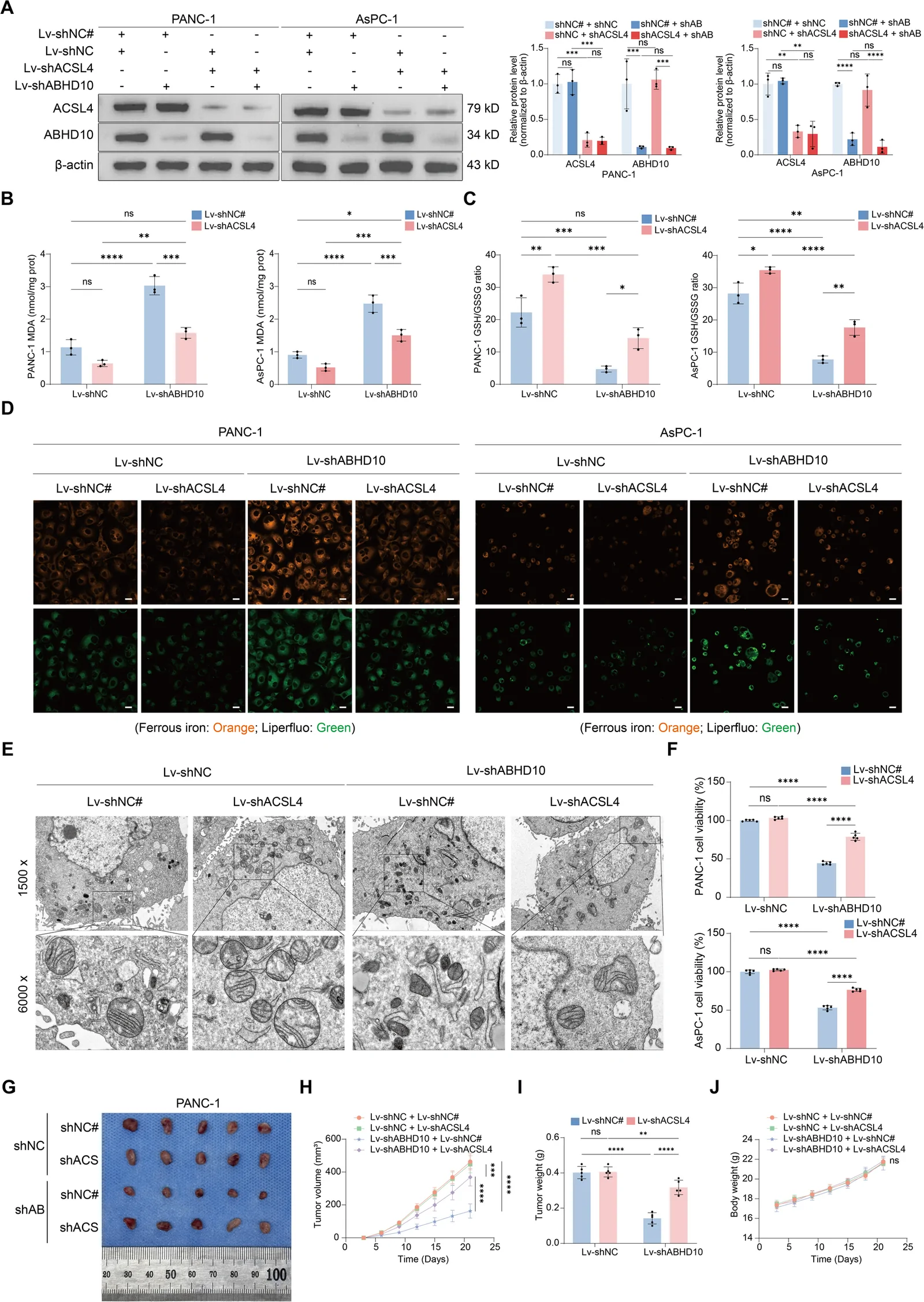
ACSL4 knockdown rescues ABHD10 loss-induced ferroptosis and growth suppression in PDAC cells. **A** Western blot showing ABHD10 and ACSL4 knockdown in PANC-1 and AsPC-1 cells under the indicated genetic combinations (Lv-shNC, Lv-shACSL4, Lv-shABHD10, and Lv-shABHD10 + Lv-shACSL4). **B** MDA levels showing lipid peroxidation in PANC-1 (left) and AsPC-1 (right) cells under the indicated genetic conditions. **C** GSH/GSSG ratios showing redox status in PANC-1 (left) and AsPC-1 (right) cells under the indicated genetic conditions. **D** Representative fluorescence images showing labile ferrous iron (Fe²⁺; orange) and lipid peroxidation (Liperfluo; green) in PANC-1 (left) and AsPC-1 (right) cells with the indicated genotypes. Scale bar, 20 μm. **E** TEM images showing mitochondrial ultrastructure in PANC-1 cells with the indicated genotypes. Scale bars, 2 μm and 500 nm. **F** CCK-8 assays showing cell viability in PANC-1 (top) and AsPC-1 (bottom) cells with the indicated genotypes. **G** Representative images showing tumors at the experimental endpoint from PANC-1 xenografts with the indicated genotypes. **H **Tumor growth curves showing xenograft growth over time. **I** Tumor weights showing endpoint tumor burden. **J** Body weight curves showing mouse body weight over time. Lv-shNC and Lv-shNC# denote the non-targeting negative-control lentiviruses corresponding to the ABHD10-shRNA and ACSL4-shRNA systems, respectively; Lv-shNC + Lv-shNC# denotes the double-negative control group, in which cells were transduced with both negative-control lentiviruses. Data are presented as mean ± SEM. Statistical analyses were performed using two-way ANOVA. For biochemical endpoints (**B**–**C**), the selected pairwise comparisons included the comparison between the double-negative control group (Lv-shNC + Lv-shNC#) and the double-knockdown group (Lv-shABHD10 + Lv-shACSL4) to directly assess the extent of rescue. *p < 0.05; **p < 0.01; ***p < 0.001; ****p < 0.0001; ns, not significant

To determine whether ACSL4 mediates ABHD10-regulated PDAC growth, we performed combined genetic ablation of both genes. We engineered PANC-1 and AsPC-1 cells to establish four distinct genotypes: control (Lv-shNC + Lv-shNC#), ACSL4-knockdown (Lv-shNC + Lv-shACSL4), ABHD10-knockdown (Lv-shNC# + Lv-shABHD10), and ACSL4/ABHD10 double-knockdown (Lv-shACSL4 + Lv-shABHD10) (Fig. 7A). As shown in Figs. 7B–D, ACSL4 knockdown, consistent with its role as a key ferroptosis effector, caused modest changes in basal ferroptosis-related biochemical markers, including lipid peroxidation products (MDA and lipid ROS) and the labile iron pool (Fe²⁺), while increasing the GSH/GSSG ratio. Conversely, ABHD10 knockdown alone produced the opposite biochemical profile, elevating oxidative stress and depleting antioxidant capacity. Critically, concurrent knockdown of ACSL4 in ABHD10‑deficient cells substantially, though incompletely, rescued the pro‑ferroptotic state induced by ABHD10 loss. Specifically, it significantly attenuated the elevation of lipid peroxidation (measured by LPO and MDA) and the dysregulated labile iron pool, and partially restored the glutathione redox balance (GSH/GSSG ratio). Direct statistical comparison between the double-negative control and ACSL4/ABHD10 double-knockdown groups showed that MDA levels and GSH/GSSG ratios were restored close to baseline in PANC-1 cells, whereas significant differences remained in AsPC-1 cells. These findings indicate a substantial but incomplete and cell line-dependent biochemical rescue. As shown in Fig. 7E, this genetic rescue was also evident at the ultrastructural level: transmission electron microscopy showed that ferroptosis-related mitochondrial damage (membrane condensation, increased membrane density, and cristae loss) triggered by ABHD10 knockdown was substantially mitigated by co‑knockdown with ACSL4. Consistently, the severe deficits in cell viability (CCK‑8), clonogenic capacity, and in vivo tumor growth caused by ABHD10 loss were significantly reversed by concomitant ACSL4 ablation, though incompletely (Fig. 7F–J; Figs. S9A). Notably, ACSL4 knockdown alone caused modest changes in ferroptosis-related biochemical markers, but these basal biochemical shifts did not translate into detectable growth inhibition, mitochondrial ultrastructural damage, or xenograft suppression in the absence of ABHD10 loss (Figs. 7E–I). In xenograft tumors, ABHD10 knockdown increased ACSL4 palmitoylation and elevated the lipid peroxidation marker 4-Hydroxynonenal (4-HNE), whereas ACSL4 knockdown attenuated the increase in 4-HNE (Fig. S9B, C). These data support in vivo activation of ACSL4-dependent lipid peroxidation upon ABHD10 depletion. This incomplete rescue suggests that, although ACSL4 is the dominant downstream effector through which ABHD10 drives lipid peroxidation and ferroptosis resistance, ABHD10 may also regulate additional ACSL4-independent molecules that contribute to its oncogenic function. In summary, these genetic epistasis experiments establish that the pro‑tumorigenic activity of ABHD10 is largely mediated through ACSL4. Depalmitoylation of ACSL4 by ABHD10 therefore represents a central mechanism through which ABHD10 suppresses ferroptosis.

### ACSL4 mediates the anti-tumor effects of HF

**Fig. 8:**
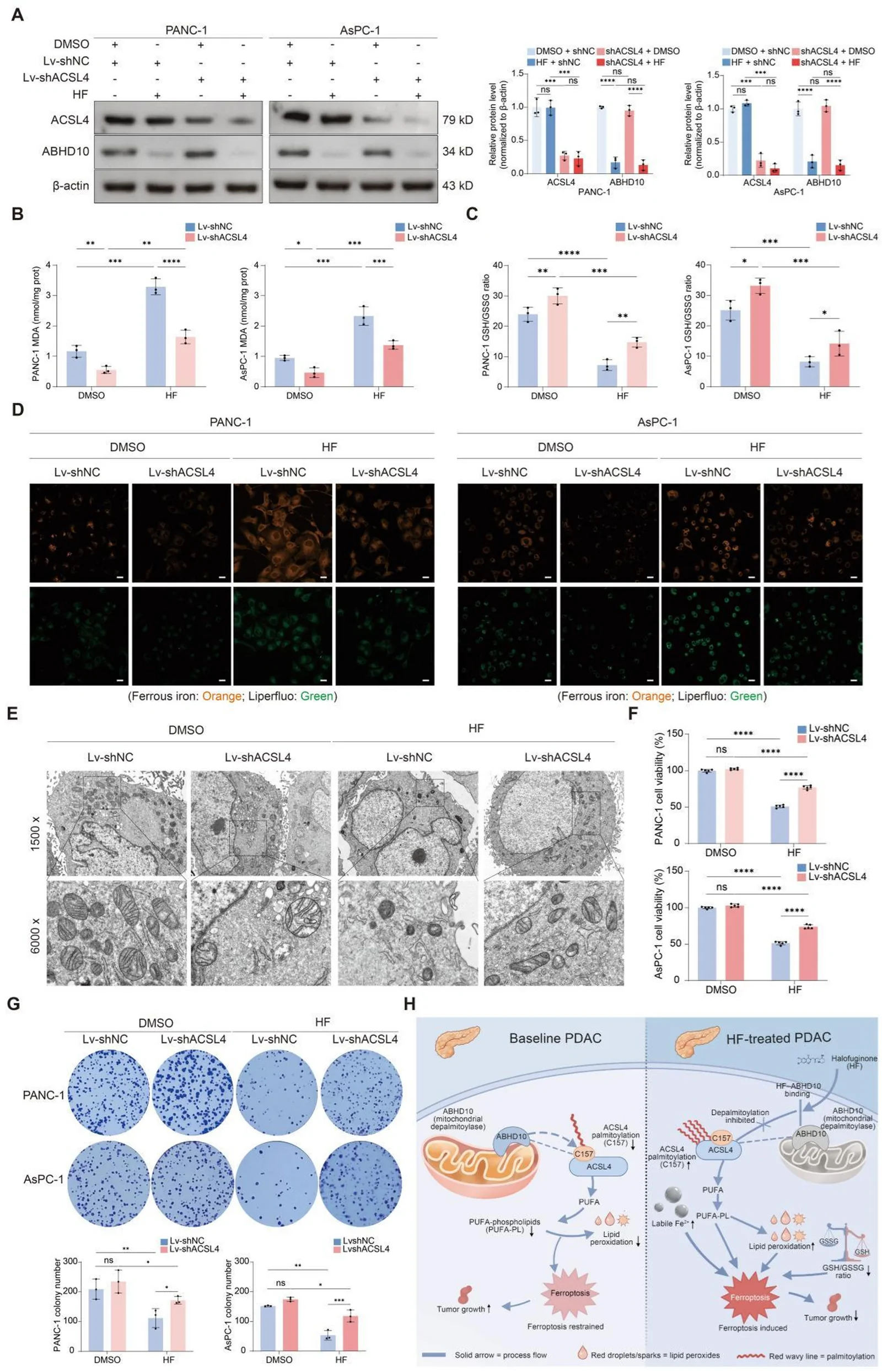
Genetic rescue of HF-induced phenotypes by ACSL4 depletion. **A** Western blot showing ACSL4 knockdown and ABHD10 expression in PANC-1 and AsPC-1 cells under the indicated conditions (Lv-shNC or Lv-shACSL4, treated with DMSO or HF). **B** MDA levels showing lipid peroxidation in PANC-1 (left) and AsPC-1 (right) cells under the indicated conditions. **C** GSH/GSSG ratios showing redox status in PANC-1 (left) and AsPC-1 (right) cells under the indicated conditions. **D** Fluorescence images showing labile ferrous iron (Fe²⁺; orange) and lipid peroxidation (Liperfluo; green) in PANC-1 (left) and AsPC-1 (right) cells under the indicated conditions. Scale bar, 20 μm. **E** TEM images showing mitochondrial ultrastructure in PANC-1 cells under the indicated conditions. Scale bars, 2 μm and 500 nm. **F** CCK-8 assays showing cell viability in PANC-1 (top) and AsPC-1 (bottom) cells under the indicated conditions. **G** Colony formation assays showing clonogenic capacity in PANC‑1 and AsPC‑1 cells under the indicated conditions, with quantification shown. **H** Schematic model illustrating ferroptosis regulation through the ABHD10–ACSL4 axis in PDAC. In PDAC, ABHD10 functions as a mitochondrial depalmitoylase that removes palmitoyl groups from ACSL4 at C157, thereby limiting ACSL4 palmitoylation and restraining lipid peroxidation and ferroptosis. HF binds ABHD10 and functionally interferes with ABHD10-dependent ACSL4 depalmitoylation in cells, resulting in increased ACSL4 palmitoylation at C157, enhanced lipid peroxidation, elevated labile Fe²⁺ levels, disruption of the GSH/GSSG redox balance, and induction of ferroptosis, ultimately suppressing tumor growth. Data in quantitative panels are presented as mean ± SEM. Statistical analyses were performed using two-way ANOVA. *p < 0.05; **p < 0.01; ***p < 0.001; ****p < 0.0001; ns, not significant

To determine whether ACSL4 mediates the effects of HF treatment, we assessed the biochemical, morphological, and functional consequences of HF in pancreatic cancer cells with or without stable ACSL4 knockdown. The experiment comprised four groups: control (Lv-shNC + DMSO), ACSL4-knockdown alone (Lv-shACSL4 + DMSO), HF treatment alone (Lv-shNC + HF), and the combination (Lv-shACSL4 + HF) (Fig. 8A). As shown in Figs. 8B–D, HF treatment alone induced a pronounced pro-ferroptotic state, characterized by significantly elevated lipid peroxidation (LPO and MDA) and labile iron (Fe²⁺) levels, along with impaired glutathione redox balance (a reduced GSH/GSSG ratio) in both PANC-1 and AsPC-1 cells. Conversely, ACSL4 knockdown alone produced an opposite baseline profile. Critically, concurrent knockdown of ACSL4 significantly, though not completely, mitigated the HF-driven biochemical perturbations. ACSL4 knockdown substantially reduced the HF-induced elevations in LPO, MDA, and Fe²⁺ and partially restored the depleted GSH/GSSG ratio. As shown in Fig. 8E, this partial biochemical rescue was mirrored at the ultrastructural level in PANC-1 cells: while HF treatment triggered characteristic ferroptotic mitochondrial damage (condensation, increased membrane density, and loss of cristae), concurrent ACSL4 knockdown substantially ameliorated, but did not completely prevent these morphological alterations. Functionally, the loss of ACSL4 conferred significant, yet partial, resistance to HF, as shown by the cell viability and colony formation assays (Figs. 8F–G). The severe impairment of cell viability (CCK-8) and clonogenic capacity caused by HF treatment was significantly reduced in ACSL4 knockdown cells, but not completely eliminated. Consistently, the inhibitory effects of the combined treatment group (Lv‑shACSL4 + HF) were significantly stronger than the inhibitory effect caused by ACSL4 knockdown alone, indicating that HF retains residual ACSL4-independent anti-tumor activity. In the in vivo setting, the potent tumor‑suppressive effect of HF was significantly blunted, though not completely abrogated, in xenografts derived from ACSL4‑knockdown cells (Figs. S10A–D). In summary, the consistent pattern of partial genetic rescue across all endpoints establishes ACSL4 as the dominant, although not exclusive, effector mediating the anti‑tumor activity of HF.

## Discussion

PDAC remains a therapeutically challenging malignancy with limited effective interventions, demanding novel therapeutic strategies. Here, we identify an ABHD10–ACSL4 palmitoylation axis that promotes PDAC growth by suppressing ferroptosis. Mechanistically, ABHD10 restrains ferroptosis by depalmitoylating ACSL4 at C157, thereby limiting lipid peroxidation and supporting tumor cell growth. Using HF as a pharmacological entry point, we show that HF binds ABHD10 and functionally interferes with ABHD10-dependent ACSL4 depalmitoylation in cells, thereby increasing ACSL4 palmitoylation, enhancing lipid peroxidation, and triggering ferroptosis. Importantly, genetic epistasis and rescue analyses establish ACSL4 as the major downstream effector of ABHD10 in mediating ferroptosis suppression and growth promotion. Together, our findings establish ABHD10 as an oncogenic driver in PDAC and unveil dynamic ACSL4 palmitoylation as a crucial post-translational regulatory layer of ferroptosis.

One of the main contributions of this study is elucidating the ABHD10–ACSL4–ferroptosis pathway as a mechanistic basis for PDAC growth control. HF has shown anti‑cancer activity through diverse mechanisms, primarily via inhibition of prolyl‑tRNA synthetase (ProRS) [37, 46] and modulation of signaling cascades including the PI3K/AKT/MAPK and TGF‑β [47, 48]. However, these observations have not identified the direct molecular target driving its growth-suppressive effect in PDAC. By identifying ABHD10 as a direct binding target of HF, our study uses this small molecule as a mechanistic entry point to uncover a palmitoylation-dependent ferroptosis pathway. Thus, the major conceptual advance of this work is not simply the characterization of HF, but the identification of ABHD10-dependent regulation of ACSL4 palmitoylation as a determinant of ferroptosis sensitivity and PDAC growth. This target-based understanding is particularly important for PDAC, as rational drug optimization, biomarker development, and the design of combination therapy strategies all rely on identifying the upstream target, rather than merely observing downstream growth-inhibitory phenotypes.

Beyond identifying ABHD10 as a target of HF, our data position this mitochondrial depalmitoylase, previously not linked to HF, as a functionally important driver in PDAC [39, 49]. The ABHD enzyme family is defined by a conserved α/β-hydrolase fold, characterized by a central β-sheet of eight strands with the second strand oriented antiparallel [50–52]. In humans, the ABHD family comprises 23 isoforms [53, 54]. Among them, ABHD10 has been recognized as a mitochondrial depalmitoylase involved in maintaining organelle integrity and sperm motility [40, 55]; however, its pathophysiological functions, particularly in cancer, remain poorly understood. Here, we establish ABHD10 as more than a binding partner, but as a functionally important driver of PDAC growth and survival. Consistent with this, pharmacological perturbation showed little additivity with ABHD10 loss- or gain-of-function across growth-related phenotypes, supporting ABHD10 as the major functional mediator in this pathway. In particular, the attenuated response to HF in ABHD10-overexpressing cells and xenografts may reflect a buffering or pathway-rescue effect under the fixed HF exposure conditions used in this study. Because these experiments were not designed to establish saturating ABHD10 target engagement, increasing ABHD10 abundance may enlarge the target pool and maintain residual ABHD10 regulatory capacity, thereby counteracting HF-induced ACSL4 palmitoylation-dependent ferroptotic signaling rather than necessarily increasing HF sensitivity. This oncogenic capacity is likely linked to its depalmitoylase role, which appears to support PDAC growth and survival. This view aligns with emerging evidence positioning other ABHD family members, such as ABHD12 in liver cancer, as key players in tumor biology, further highlighting the therapeutic relevance of this enzyme family in PDAC [56]. Importantly, our findings suggest that ABHD10 may represent a tractable node for therapeutic intervention.

Ferroptosis, an iron-dependent form of cell death driven by lipid peroxidation of phospholipids containing polyunsaturated fatty acids (PUFAs) [57–59], is critically regulated by ACSL4. ACSL4 is widely recognized as a key determinant of ferroptosis by promoting the incorporation of PUFAs into phospholipids [60, 61]. While ACSL4 has been studied at the transcriptional and translational levels, the post-translational mechanisms that regulate its ferroptosis-related role remain poorly understood. Through unbiased multi-omic analyses, we identified ACSL4 as a major downstream effector of the depalmitoylase ABHD10 and pinpointed C157 palmitoylation as a site-specific regulatory event associated with ferroptosis sensitivity in PDAC. Indeed, palmitoylation circuits are increasingly recognized as critical regulators of ferroptosis in cancer [17, 62, 63]; in PDAC, for instance, both ZDHHC2- and ABHD17C-associated pathways have been implicated in ferroptosis regulation [16, 64]. Here, we demonstrate that ABHD10 directly depalmitoylates ACSL4 at C157, thereby restraining ferroptosis. Rescue and epistasis analyses further establish that this regulation operates through a causal hierarchy. Our subcellular fractionation analysis suggests that C157 palmitoylation promotes the membrane association of ACSL4, as HF increased the abundance of ACSL4-WT in the membrane fraction but had little effect on ACSL4-C157S. These findings provide a mechanistic explanation for the pro-ferroptotic role of ACSL4 C157 palmitoylation, suggesting that membrane-associated ACSL4 may participate in lipid remodeling and lipid peroxidation. Whether this modification also affects ACSL4 enzymatic activity or protein stability remains to be determined. Notably, the recent development of ABD957, which selectively inhibits the related ABHD17 depalmitoylases, underscores the druggability of this protein family [65]. Our findings thus reveal a previously unrecognized oncogenic pathway and position the ABHD10-ACSL4 axis as a promising therapeutic target in PDAC.

A consistent observation from our genetic analyses is the partial dependency of the growth-suppressive effects associated with ABHD10 loss on ACSL4. Importantly, the functional contribution of ACSL4 appeared to be context-dependent: ACSL4 knockdown alone caused modest basal biochemical changes without detectable functional effects, whereas its impact became evident following ABHD10 depletion. One possible but untested explanation is that these basal changes did not reach a level sufficient to produce measurable functional consequences. However, the rescue by ACSL4 co-knockdown was not complete across all biochemical markers and cell lines, suggesting that ACSL4 acts as a major downstream effector of ABHD10 while additional ACSL4-independent mechanisms may also contribute to ABHD10-mediated ferroptosis regulation. While ACSL4 deficiency substantially attenuated the response to HF treatment, supporting its role as a major downstream effector of the HF-responsive pathway, the incomplete rescue points to additional regulatory layers. These likely include other ABHD10 substrates and potentially ABHD10-independent activities of HF, which collectively shape the cellular oxidative stress landscape [34, 40]. This layered regulatory model aligns with the complexity of PDAC, where multiple redox and lipid-metabolic programs converge [66–68]. It thus provides a rationale for combination strategies designed to broaden ferroptosis susceptibility beyond a single regulatory node.

To place these findings in a translational context, we analyzed xenograft tumors and public PDAC datasets. ABHD10 depletion increased ACSL4 palmitoylation and 4-HNE accumulation in tumors, whereas ACSL4 knockdown attenuated the increase in 4-HNE, supporting in vivo engagement of the ABHD10–ACSL4–lipid peroxidation axis. Transcriptomic analyses of published data further linked ABHD10 expression to ferroptosis-, oxidative stress-, lipid oxidation-, ROS-, and fatty acid metabolism-related signatures. However, ABHD10 expression alone was not significantly associated with overall survival, suggesting that ABHD10 may represent a pathway-related therapeutic vulnerability rather than a standalone prognostic biomarker. These findings suggest several translational opportunities. ABHD10 expression or pathway dependence may help identify tumors with active ABHD10–ACSL4 depalmitoylation circuitry, while ACSL4 palmitoylation and lipid peroxidation markers may serve as useful mechanistic readouts for pathway engagement. More broadly, our data support therapeutic strategies aimed at disrupting the ABHD10–ACSL4 axis to relieve ferroptosis suppression with combined redox- or lipid-modulating agents.

Nevertheless, several unanswered questions and limitations remain to be addressed before this strategy can advance to clinics. Structurally, our modeling suggests that the predicted ABHD10-binding site on ACSL4 is distant from the C157 regulatory residue, raising the possibility of an unconventional regulatory mechanism. Elucidating how ABHD10 recognizes ACSL4 and controls C157 depalmitoylation requires dedicated structural and biochemical investigation. From a pharmacological perspective, the SPR-derived K_D_ value (2.41 µM) of HF binding to recombinant ABHD10 was higher than its cellular IC₅₀ (27 nM), indicating that biochemical affinity and cellular potency are not directly interchangeable. This difference may reflect cellular exposure, downstream ferroptotic signal amplification, and/or ABHD10-independent activities of HF [37, 46–48]. ABHD10 overexpression may buffer HF activity under the fixed HF exposure conditions used in this study, which may explain the attenuated response to HF in ABHD10-overexpressing models. Future target-occupancy, dose-response, and structure-activity studies are needed to define the relationship between ABHD10 abundance, HF engagement, and cellular potency. Although HF functionally interferes with ABHD10-dependent ACSL4 depalmitoylation in cells, these assays do not directly assess the intrinsic catalytic activity of ABHD10. Importantly, HF should not be viewed as a selective ABHD10 inhibitor, given its reported multi-target activities; therefore, ABHD10-independent effects may contribute to both its anti-tumor activity and potential toxicity. In addition, because ABHD10 has physiological functions as a mitochondrial depalmitoylase [40, 55], the selectivity and safety of ABHD10 inhibition require careful evaluation. Although the HF regimen was literature-based, not intended to represent the MTD, and generally well tolerated in our study, formal dose-ranging and MTD studies were not performed; future studies are needed to define the optimal dose, therapeutic window, off-target profile, and safety of HF or optimized ABHD10-targeting compounds. Moreover, given the profound influence of the PDAC microenvironment on drug exposure and ferroptosis sensitivity, efficacy and safety should be validated in more clinically representative models (e.g., orthotopic and patient-derived systems incorporating stromal and immune contexts), and rational combination regimens should also be evaluated with careful attention to on-target toxicity. Furthermore, the use of only male BALB/c nude mice limited our ability to assess potential sex-dependent effects on PDAC growth and ABHD10–ACSL4-mediated ferroptosis, which should be addressed in future studies using both sexes.

In summary, we identify ABHD10 as a major functional mediator of HF and define an ABHD10-ACSL4 palmitoylation circuit that controls ferroptosis sensitivity in PDAC. By showing that ABHD10 depalmitoylates ACSL4 at C157 to restrain ferroptosis, and that this pathway promotes PDAC growth by maintaining ferroptosis suppression, our work establishes palmitoylation-dependent ferroptosis as a therapeutically targetable vulnerability in PDAC (Fig. 8H). These findings provide a mechanistic rationale for targeting the ABHD10–ACSL4 axis and support palmitoylation-dependent ferroptosis as a therapeutically actionable vulnerability in PDAC.

## Conclusions

ABHD10 promotes PDAC growth by depalmitoylating ACSL4 at C157, suppressing ferroptosis. Inhibition of this pathway increases ferroptotic vulnerability and suppresses tumor growth. These findings identify the ABHD10–ACSL4 axis as a mechanistically relevant driver of PDAC progression and a promising therapeutic target in PDAC.

## Supplementary Information

Supplementary Material 1.

## Data Availability

The data that support the findings of this study are available from the corresponding authors upon reasonable request.

## Abbreviations

PDAC: Pancreatic ductal adenocarcinoma
ABHD10: Alpha/beta hydrolase domain-containing protein 10
HF: Halofuginone
ACSL4: Acyl-CoA synthetase long-chain family member 4
DMSO: Dimethyl sulfoxide
MTD: Maximum tolerated dose
PBS: Phosphate-buffered saline
hTERT-HPNE: Human telomerase reverse transcriptase-immortalized human pancreatic nestin-expressing cells
STR: Short tandem repeat
DMEM: Dulbecco’s modified Eagle’s medium
FBS: Fetal bovine serum
CCK‑8: Cell Counting Kit‑8
IC_50_: Half‑maximal inhibitory concentration
HTS: High-throughput screening
PFA: Paraformaldehyde
WB: Western blot
BCA: Bicinchoninic acid protein assay
SDS: Sodium dodecyl sulfate
PVDF: Polyvinylidene difluoride
TBST: Tris-buffered saline with Tween 20
HRP: Horseradish peroxidase
ECL: Enhanced chemiluminescence
IF: Immunofluorescence
DAPI: 4′,6‑diamidino‑2‑phenylindole
LiP-MS: Limited proteolysis-mass spectrometry
LC-MS/MS: Liquid chromatography-tandem mass spectrometry
DIA: Data-independent acquisition
DARTS: Drug affinity responsive target stability
MCE: MedChemExpress
CETSA: Cellular thermal shift assay
shRNA: Short hairpin RNA
NC: Non-targeting control
qRT-PCR: Quantitative real-time polymerase chain reaction
cDNA: Complementary DNA
Co‑IP: Co‑immunoprecipitation
IgG: Immunoglobulin G
RIN: RNA integrity number
NEM: N-ethylmaleimide
HA/HAM: Hydroxylamine
FDR: False discovery rate
2‑BP: 2-bromopalmitate
ABE: Acyl-biotinyl exchange
MDA: Malondialdehyde
TBA: Thiobarbituric acid
Lipid ROS: Lipid reactive oxygen species
GSH: Reduced glutathione
GSSG: Oxidized glutathione
TEM: Transmission electron microscopy
SPR: Surface plasmon resonance
EDC: 1-ethyl-3-(3-dimethylaminopropyl)carbodiimide
NHS: N-hydroxysuccinimide
EMT: Epithelial-to-mesenchymal transition
WT: Wild‑type
PUFAs: Polyunsaturated fatty acids
TCGA: The Cancer Genome Atlas
K_D_: Equilibrium dissociation constant
LPO: Lipid peroxidation
ProRS: Prolyl-tRNA synthetase
SD: Standard deviation
ANOVA: Analysis of variance
LOF: Loss-of-function
GOF: Gain-of-function
4-HNE: 4-Hydroxynonenal
