# Supplementary material for: The ABHD10-ACSL4 axis promotes pancreatic ductal adenocarcinoma growth via suppressing ferroptosis
Source: J Exp Clin Cancer Res. 2026 Aug 12;45:234. doi: 10.1186/s13046-026-03810-7 (PMC13749656; doi:10.1186/s13046-026-03810-7)
Supplement: Supplementary file 1 [file 13046_2026_3810_MOESM1_ESM.docx]

**Supplementary Figures**

**Figure S1.** Supplementary validation of EMT-related and motility-associated phenotypes following HF treatment in PDAC cells. (A) qRT-PCR analysis showing the expression of EMT-associated markers (E-cadherin, N-cadherin, Vimentin, Claudin-1, Fibronectin, and Snail) in PANC-1 and AsPC-1 cells treated with HF (50 nM) for 48 h. β-actin served as an internal control. (B) Western blot showing EMT-associated protein expression (E-cadherin, N-cadherin, and Vimentin) in PANC-1 and AsPC-1 cells treated with increasing concentrations of HF for 48 h. β-actin served as a loading control. (C) Transwell invasion assays showing invasive capacity of PANC-1 and AsPC-1 cells treated with the indicated concentrations of HF for 24 h. Quantification of invaded cells is shown. Scale bar, 100 µm. (D) Wound-healing assays showing migratory capacity of PANC‑1 and AsPC‑1 cells treated with the indicated concentrations of HF. Representative images at 0 h and 48 h are shown, with quantification of wound closure. Scale bar, 200 µm. Data are presented as mean ± SEM. Statistical analyses were performed using unpaired two-tailed Student’s t-test or one-way ANOVA, as appropriate. **p* < 0.05; ***p* < 0.01; ****p* < 0.001; *****p* < 0.0001; ns, not significant.

**Figure S2.** LiP-MS identification of ABHD10 as an HF-associated target and public-dataset assessment of its prognostic relevance. (A) Venn diagram showing the overlap of candidate proteins identified from LiP‑MS analysis across different HF concentration groups. ABHD10 is indicated as the top overlapping hit. (B) Mapping of LiP-MS peptide signatures onto the ABHD10 sequence. The theoretical sequence is shown in black; grey areas indicate undetected or unchanged regions. Peptides significantly upregulated (orange) or downregulated (purple) upon HF treatment are highlighted, identifying protected and exposed regions. Amino-acid positions are numbered. (C) Kaplan–Meier overall survival analysis of TCGA-PAAD patients stratified into ABHD10-high and ABHD10-low groups according to the median ABHD10 expression level, together with a summary forest plot of univariate and multivariate Cox regression results for ABHD10 in the TCGA, GSE183795, and GSE62452 cohorts. The x-axis indicates survival time in years, and the y-axis indicates the estimated overall survival probability. The number-at-risk table below the curve shows the number of patients remaining under follow-up at each indicated time point. The survival difference was assessed using the log-rank test. Diamonds indicate hazard ratios (HRs), horizontal lines indicate 95% confidence intervals (CIs), and the dashed vertical line indicates HR = 1. (D) Kaplan–Meier overall survival curves for patients in the GSE183795, GSE62452, and GSE28735 cohorts stratified according to the median ABHD10 expression level. Red and blue curves represent the high- and low-expression groups, respectively. Numbers at risk are shown below each plot, and log-rank *p* values are indicated. (E) Univariate (left) and multivariate (right) Cox regression analyses of ABHD10 expression and clinicopathological variables in the TCGA, GSE183795, and GSE62452 cohorts. Diamonds represent HRs, horizontal lines represent 95% CIs, and the dashed vertical line indicates HR = 1. Corresponding *p* values and HRs with 95% CIs are listed alongside each forest plot.

**Figure S3.** Validation of ABHD10 knockdown in PDAC cells. (A) qRT-PCR analysis showing ABHD10 mRNA levels in PANC-1 and AsPC-1 cells with stable ABHD10 knockdown. (B) Western blot showing ABHD10 protein levels in PANC-1 and AsPC-1 cells with stable ABHD10 knockdown. Data are presented as mean ± SEM. Statistical analyses were performed using one-way ANOVA. **p* < 0.05; ***p* < 0.01; ****p* < 0.001; *****p* < 0.0001.

**Figure S4.** Supplementary characterization of phenotypic changes in ABHD10-knockdown cells. (A) Transwell invasion assays showing invasive capacity of PANC-1 and AsPC-1 cells with stable ABHD10 knockdown (Lv-shABHD10) or control (Lv-shNC), treated with vehicle or HF (50 nM) for 24 h, with quantification shown. Scale bar, 100 µm. (B) Wound-healing assays showing migratory capacity of PANC-1 and AsPC-1 cells with stable ABHD10 knockdown (Lv-shABHD10) or control (Lv-shNC), treated with vehicle or HF (50 nM). Representative images at 0 h and 48 h are shown, with quantification of wound closure. Scale bar, 200 µm. (C) Western blot showing EMT-associated protein expression (E-cadherin, N-cadherin, and Vimentin) in PANC-1 and AsPC-1 cells with stable ABHD10 knockdown (Lv-shABHD10) or control (Lv-shNC), treated with vehicle or HF (50 nM) for 48 h, with quantification shown. Data are presented as mean ± SEM. Statistical analyses were performed using two-way ANOVA. **p* < 0.05; ***p* < 0.01; ****p* < 0.001; *****p* < 0.0001; ns, not significant.

**Figure S5.** Validation of ABHD10 overexpression in PDAC cells. (A) qRT-PCR analysis showing ABHD10 mRNA levels in PANC-1 and AsPC-1 cells with ABHD10 overexpression. (B) Western blot showing ABHD10 protein levels in PANC-1 and AsPC-1 cells with ABHD10 overexpression. Data are presented as mean ± SEM. Statistical analyses were performed using unpaired two-tailed Student’s t-test. **p* < 0.05; ***p* < 0.01; ****p* < 0.001; *****p* < 0.0001.

**Figure S6.** Supplementary characterization of phenotypic changes in ABHD10-overexpressing cells. (A) Transwell invasion assays showing invasive capacity of PANC-1 and AsPC-1 cells with ABHD10 overexpression (Lv-ABHD10) or control (Lv-NC), treated with vehicle or HF (50 nM) for 24 h, with quantification shown. Scale bar, 100 µm. (B) Wound-healing assays showing migratory capacity of PANC-1 and AsPC-1 cells with ABHD10 overexpression (Lv-ABHD10) or control (Lv-NC), treated with vehicle or HF (50 nM). Representative images at 0 h and 48 h are shown, with quantification of wound closure. Scale bar, 200 µm. (C) Western blot showing EMT-associated protein expression (E-cadherin, N-cadherin, and Vimentin) in PANC-1 and AsPC-1 cells with ABHD10 overexpression (Lv-ABHD10) or control (Lv-NC), treated with vehicle or HF (50 nM) for 48 h, with quantification shown. Data are presented as mean ± SEM. Statistical analyses were performed using two-way ANOVA. **p* < 0.05; ***p* < 0.01; ****p* < 0.001; *****p* < 0.0001; ns, not significant.

**Figure S7.** Association between ABHD10 expression and ferroptosis-related transcriptional programs in public PDAC datasets. (A) Spearman correlation analyses between ABHD10 expression and enrichment scores for GOBP ferroptosis, WP ferroptosis, the reactive oxygen species pathway, fatty acid metabolism, response to oxidative stress, and lipid oxidation. The x-axis represents ABHD10 expression, and the y-axis represents the corresponding pathway enrichment score. The red fitted line indicates the correlation trend. Marginal histograms show the distributions of ABHD10 expression and pathway scores. Spearman correlation coefficients (r) and corresponding *p* values are displayed in each panel. (B) Heatmap showing Spearman correlations between ABHD10 expression and ferroptosis- and lipid metabolism-related genes. The color scale ranges from blue, indicating a negative correlation, to red, indicating a positive correlation, according to the Spearman correlation coefficient (r). Numbers within the cells indicate correlation coefficients, and asterisks indicate statistical significance. **p* < 0.05; ***p* < 0.01; ****p* < 0.001.

**Figure S8.** Supporting data for ABHD10-dependent regulation of ACSL4 palmitoylation and ferroptosis-related phenotypes. (A) ABE assay showing ACSL4 palmitoylation (±HAM) in PANC-1 and AsPC-1 cells with ABHD10 overexpression (Lv-ABHD10) or control (Lv-NC). (B) ABE assay showing ACSL4 palmitoylation in PANC-1 and AsPC-1 cells treated with DMSO or HF. (C) Short-term HF time-course ABE assay showing ACSL4 palmitoylation in PANC-1 and AsPC-1 cells treated with 50 nM HF for the indicated times. (D) ABE assay showing ACSL4 palmitoylation in Lv-shNC and Lv-shABHD10 PANC-1 and AsPC-1 cells treated with DMSO or HF. (E) Click-iT assay showing ACSL4 palmitoylation in HEK293T cells transfected with plasmids encoding ABHD10-Flag, together with ACSL4-HA (WT) or its C157S mutant for 48 h. (F) Subcellular fractionation analysis of ACSL4-WT and ACSL4-C157S distribution in PANC-1 and AsPC-1 cells treated with DMSO or HF. Membrane and cytosolic fractions were analyzed by Western blotting, with ATP1A1 and β-actin used as fraction markers. The membrane/cytoplasm ratio was calculated as (ACSL4_membrane_/ATP1A1)/(ACSL4_cytoplasm_/β-actin) and normalized to the WT + DMSO group. (G) GSH/GSSG ratios showing redox status in PANC-1 and AsPC-1 cells under the indicated conditions (Vec + Lv-shNC, Vec + Lv-shABHD10, Lv-shABHD10 + ACSL4-WT, and Lv-shABHD10 + ACSL4-C157S). (H) MDA levels showing lipid peroxidation in PANC-1 and AsPC-1 cells under the indicated conditions (Vec + Lv-shNC, Vec + Lv-shABHD10, Lv-shABHD10 + ACSL4-WT, and Lv-shABHD10 + ACSL4-C157S). Data are presented as mean ± SEM. Statistical analyses were performed using unpaired two-tailed Student’s t-test, two-way ANOVA, or one-way ANOVA, as appropriate. **p* < 0.05; ***p* < 0.01; ****p* < 0.001; *****p* < 0.0001; ns, not significant.

**Figure S9.** Additional validation of the ABHD10–ACSL4 genetic interaction and *in vivo* ferroptosis-related changes. (A) Colony formation assays showing clonogenic capacity of PANC-1 and AsPC-1 cells transduced with control (shNC), shACSL4, shABHD10, or shACSL4 + shABHD10 lentiviruses, with quantification shown. (B) ABE assay showing ACSL4 palmitoylation in xenograft tumor tissues derived from the indicated PANC-1 cell lines. Total palmitoylated ACSL4 signal was quantified from the +HAM streptavidin signal after subtraction of the -HAM background. Relative ACSL4 palmitoylation level was calculated as palmitoylated ACSL4 signal normalized to total ACSL4 protein. (C) Western blot analysis of 4-HNE levels in xenograft tumor tissues from the indicated groups. 4-HNE signals were quantified as background-corrected total lane intensity within the 25–100 kDa range and normalized to the Lv-shNC + Lv-shNC# group. Equal protein loading was verified by β-actin immunoblotting performed in parallel. Data are presented as mean ± SEM. Statistical analyses were performed using unpaired two-tailed Student’s t-test or two-way ANOVA. **p* < 0.05; ***p* < 0.01; ****p* < 0.001; *****p* < 0.0001; ns, not significant.

**Figure S10.** Additional validation that ACSL4 depletion rescues the growth-suppressive effects of HF *in vivo*. (A–D) *In vivo* tumor growth of PANC-1 xenografts under the indicated conditions. (A) Representative images showing tumors at the endpoint. (B) Tumor growth curves showing xenograft growth over time. (C) Tumor weights showing endpoint tumor burden. (D) Body weight curves showing mouse body weight over time. Data are presented as mean ± SEM. Statistical analyses were performed using two-way ANOVA. **p* < 0.05; ***p* < 0.01; ****p* < 0.001; *****p* < 0.0001; ns, not significan.

**Supplementary Tables**

Table S1. Clinical information of pancreatic cancer patients

| Gender | Age | Diagnosis |
| --- | --- | --- |
| Female | 71 | Moderately differentiated pancreatic ductal adenocarcinoma |
| Male | 53 | Moderately differentiated pancreatic adenocarcinoma |
| Male | 68 | Poorly differentiated pancreatic ductal adenocarcinoma |
| Female | 67 | Moderately to poorly differentiated pancreatic adenocarcinoma |
| Male | 68 | Moderately to poorly differentiated pancreatic adenocarcinoma |
| Female | 68 | Moderately differentiated pancreatic adenocarcinoma |
| Male | 47 | Poorly differentiated pancreatic adenocarcinoma |
| Male | 53 | Moderately differentiated pancreatic adenocarcinoma |
| Female | 59 | Pancreatic ductal adenocarcinoma |
| Female | 70 | Moderately to poorly differentiated pancreatic ductal adenocarcinoma |

Table S2. Antibody information

| Name | Catalog Number | Supplier company | Dilution ratio |
| --- | --- | --- | --- |
| N Cadherin Rabbit pAb | 380671 | ZenBio | 1:1000 |
| E Cadherin Rabbit pAb | 340341 | ZenBio | 1:1000 |
| Vimentin Rabbit mAb | R22775 | ZenBio | 1:10000 |
| beta Actin Mouse mAb (HRP) | 700068 | ZenBio | 1:5000 |
| Recombinant human ABHD10 protein | Ag18413 | Proteintech | 50 µg/mL for SPR |
| Recombinant Human ACSL4 Protein | IPD-X50097 | IPODIX | 50 µg/mL for SPR |
| 4-Hydroxynonenal Monoclonal antibody | 68538-1-1g | Proteintech | 1：500 for WB |
| ATP1A1 Polyclonal antibody | 14418-1-AP | Proteintech | 1:20000 for WB |
| ABHD10 Polyclonal antibody | 22558-1-AP | Proteintech | 1:1000 for WB |
| ABHD10 Polyclonal Antibody | PA5-103553 | Thermo Scientific | 1:200 for IHC, IF |
| TOM20 Polyclonal antibody | 11802-1-AP | Proteintech | 1:200 for IF |
| Annexin V Monoclonal antibody | 66245-1-Ig | Proteintech | 1:400 for IF |
| ANTI-FLAG® M2 antibody, M | F1804 | Sigma-Aldrich | 1:50 for IP, 1:1000 for WB |
| Goat Anti-Rabbit IgG H&L（HRP） | 511023 | ZenBio | 1:5000 |
| Anti-HA tag | 81290-1RR | Proteintech | 1:200 For IP |
| ACSL4/FACL4 Monoclonal antibody | 66617-1-Ig | Proteintech | 1:3000 for WB, 1:200 for IF |
| FACL4 Antibody | T510198 | Abmart | 1:50 for IP |
| Universal anti-heavy and light chain secondary antibody | M21008 | Abmart | 1:1000 for WB |
| HRP-conjugated Streptavidin | SA00001-0 | Proteintech | 1:2000 for WB |
| HRP-conjugated Goat Anti-Rabbit IgG (H+L) | ZB-2301 | Zhongshan Jinqiao | 1:2500 for WB |
| HRP-conjugated Goat Anti-Mouse IgG (H+L) | ZB-2305 | Zhongshan Jinqiao | 1:2500 for WB |
| DyLight 488, Goat Anti-Mouse IgG | A23210 | Abbkine | 1:200 for IF |
| Dylight 594, Goat Anti-Rabbit IgG | A23420 | Abbkine | 1:200 for IF |

Table S3. Details of plasmid construction

| Plasmid Name | Backbone | Sequence |
| --- | --- | --- |
| 3Xflag-ABHD10 | pcDNA3.1(+) | GATTACAAAGACCATGACGGCGACTATAAAGATCACGACATCGACTACAAAGACGACGATGACAAG |
| HA-ACSL4 | pcDNA3.1(+) | CTGAAGCCTAAGAACACCATTGCCATCTTC**TGC**GAGACGAGAGCCGAA |
| HA-ACSL4-C157S | pcDNA3.1(+) | CTGAAGCCTAAGAACACCATTGCCATCTTC**TCG**GAGACGAGAGCCGAA |

Table S4. The sequence of shRNAs

| shRNAs | Sequence |
| --- | --- |
| ShABHD10#1 | ACCTTAGTGACAAAGTTTAAT |
| ShABHD10#2 | CTCTAGGTCACGCCTGCATAA |
| ShACSL4 | CTGTAATAGACATCCCTGGAG |

Table S5. Primer sequences of qRT-PCR

| ABHD10-Forward | CAAAAGCGTTGGCGATTGAGGAG |
| --- | --- |
| ABHD10-Reverse | CCCAGTGTGCTTTCCTCTGAGT |
| β-actin-Forward | AGAGCTACGAGCTGCCTGAC |
| β-actin-Reverse | AGCACTGTGTTGGCGTACAG |
| E-cadherin-Forward | GCCTCCTGAAAAGAGAGTGGAAG |
| E-cadherin-Reverse | TGGCAGTGTCTCTCCAAATCCG |
| N-cadherin-Forward | CCTCCAGAGTTTACTGCCATGAC |
| N-cadherin-Reverse | GTAGGATCTCCGCCACTGATTC |
| Vimentin-Forward | AGGCAAAGCAGGAGTCCACTGA |
| Vimentin-Reverse | ATCTGGCGTTCCAGGGACTCAT |
| Claudin-1-Forward | GTCTTTGACTCCTTGCTGAATCTG |
| Claudin-1-Reverse | CACCTCATCGTCTTCCAAGCAC |
| Fibronectin-Forward | ACAACACCGAGGTGACTGAGAC |
| Fibronectin-Reverse | GGACACAACGATGCTTCCTGAG |
| Snail-Forward | TCGGAAGCCTAACTACAGCGA |
| Snail-Reverse | AGATGAGCATTGGCAGCGAG |

Table S6. Normalized cell viability of PANC-1 cells after treatment with 566 compounds

| Drug | OD1 | OD2 | OD3 | cell viability1 | cell viability2 | cell viability3 | average | DMSO | H_2_O | Blank |
| --- | --- | --- | --- | --- | --- | --- | --- | --- | --- | --- |
| Vemurafenib (PLX4032) | 0.8766 | 0.8900 | 0.8628 | 1.4898 | 1.5236 | 1.4550 | 1.4895 | 0.6823 | 0.6508 | 0.2856 |
| Salmon Calcitonin Acetate | 0.8329 | 0.7851 | 0.8190 | 1.4986 | 1.3677 | 1.4607 | 1.4423 | 0.6823 | 0.6508 | 0.2856 |
| Enzalutamide (MDV3100) | 0.8897 | 0.8363 | 0.8177 | 1.5228 | 1.3882 | 1.3414 | 1.4175 | 0.6823 | 0.6508 | 0.2856 |
| Calcium folinate | 0.8020 | 0.7902 | 0.8158 | 1.4140 | 1.3817 | 1.4518 | 1.4158 | 0.6823 | 0.6508 | 0.2856 |
| Altretamine | 0.8869 | 0.8358 | 0.8184 | 1.5158 | 1.3869 | 1.3431 | 1.4153 | 0.6823 | 0.6508 | 0.2856 |
| Artemisinin | 0.8860 | 0.8213 | 0.8285 | 1.5135 | 1.3504 | 1.3684 | 1.4108 | 0.6823 | 0.6508 | 0.2856 |
| Floxuridine (NSC 27640) | 0.8446 | 0.8726 | 0.8173 | 1.4091 | 1.4797 | 1.3403 | 1.4097 | 0.6823 | 0.6508 | 0.2856 |
| Lappaconitine | 0.8655 | 0.8468 | 0.8153 | 1.4618 | 1.4147 | 1.3353 | 1.4039 | 0.6823 | 0.6508 | 0.2856 |
| Trigonelline | 0.8504 | 0.8570 | 0.8170 | 1.4237 | 1.4404 | 1.3395 | 1.4012 | 0.6823 | 0.6508 | 0.2856 |
| Amifostine trihydrate | 0.8094 | 0.7749 | 0.8017 | 1.4343 | 1.3398 | 1.4132 | 1.3958 | 0.6823 | 0.6508 | 0.2856 |
| Drospirenone | 0.8762 | 0.8272 | 0.8117 | 1.4888 | 1.3653 | 1.3261 | 1.3934 | 0.6823 | 0.6508 | 0.2856 |
| Pemigatinib (INCB054828) | 0.8710 | 0.8520 | 0.7894 | 1.4757 | 1.4278 | 1.2699 | 1.3911 | 0.6823 | 0.6508 | 0.2856 |
| Raltitrexed | 0.8306 | 0.8800 | 0.8014 | 1.3738 | 1.4984 | 1.3002 | 1.3908 | 0.6823 | 0.6508 | 0.2856 |
| Irinotecan (CPT-11) | 0.8471 | 0.8575 | 0.8068 | 1.4154 | 1.4416 | 1.3138 | 1.3903 | 0.6823 | 0.6508 | 0.2856 |
| Naringin | 0.8603 | 0.8465 | 0.8018 | 1.4487 | 1.4139 | 1.3013 | 1.3880 | 0.6823 | 0.6508 | 0.2856 |
| Capecitabine (RO 09-1978) | 0.8036 | 0.8864 | 0.8131 | 1.3058 | 1.5145 | 1.3297 | 1.3833 | 0.6823 | 0.6508 | 0.2856 |
| Nilotinib (AMN-107) | 0.7527 | 0.9344 | 0.8134 | 1.1775 | 1.6355 | 1.3304 | 1.3811 | 0.6823 | 0.6508 | 0.2856 |
| Quinestrol | 0.8259 | 0.8675 | 0.8013 | 1.3620 | 1.4669 | 1.3001 | 1.3763 | 0.6823 | 0.6508 | 0.2856 |
| Cyclosporine | 0.8330 | 0.8166 | 0.8362 | 1.3799 | 1.3385 | 1.3880 | 1.3688 | 0.6823 | 0.6508 | 0.2856 |
| Nalidixic acid | 0.8240 | 0.8518 | 0.8083 | 1.3572 | 1.4273 | 1.3177 | 1.3674 | 0.6823 | 0.6508 | 0.2856 |
| Roquinimex | 0.8445 | 0.8296 | 0.8016 | 1.4089 | 1.3713 | 1.3008 | 1.3603 | 0.6823 | 0.6508 | 0.2856 |
| Isopropyl myristate | 0.8300 | 0.8285 | 0.8129 | 1.3723 | 1.3685 | 1.3293 | 1.3567 | 0.6823 | 0.6508 | 0.2856 |
| Oxymatrine | 0.8329 | 0.8358 | 0.8014 | 1.3796 | 1.3869 | 1.3004 | 1.3556 | 0.6823 | 0.6508 | 0.2856 |
| Lumefantrine | 0.8786 | 0.8007 | 0.7873 | 1.4948 | 1.2985 | 1.2647 | 1.3527 | 0.6823 | 0.6508 | 0.2856 |
| Ginsenoside Ro | 0.8304 | 0.8168 | 0.8127 | 1.3733 | 1.3390 | 1.3287 | 1.3470 | 0.6823 | 0.6508 | 0.2856 |
| Pemetrexed Disodium Hydrate | 0.7419 | 0.8037 | 0.7853 | 1.2495 | 1.4187 | 1.3682 | 1.3455 | 0.6823 | 0.6508 | 0.2856 |
| Clascoterone | 0.8456 | 0.8036 | 0.8075 | 1.4116 | 1.3058 | 1.3156 | 1.3443 | 0.6823 | 0.6508 | 0.2856 |
| Hypericin | 0.8203 | 0.8123 | 0.8226 | 1.3479 | 1.3277 | 1.3536 | 1.3431 | 0.6823 | 0.6508 | 0.2856 |
| Amonafide | 0.8160 | 0.8280 | 0.8018 | 1.3370 | 1.3673 | 1.3012 | 1.3352 | 0.6823 | 0.6508 | 0.2856 |
| Luteolin | 0.8031 | 0.8328 | 0.8093 | 1.3045 | 1.3794 | 1.3202 | 1.3347 | 0.6823 | 0.6508 | 0.2856 |
| Neohesperidin Dihydrochalcone (Nhdc) | 0.8407 | 0.8052 | 0.7973 | 1.3993 | 1.3098 | 1.2899 | 1.3330 | 0.6823 | 0.6508 | 0.2856 |
| Calcium Levofolinate | 0.7306 | 0.8019 | 0.7841 | 1.2185 | 1.4137 | 1.3651 | 1.3325 | 0.6823 | 0.6508 | 0.2856 |
| Gossypol | 0.8196 | 0.8163 | 0.8058 | 1.3461 | 1.3378 | 1.3114 | 1.3318 | 0.6823 | 0.6508 | 0.2856 |
| Vitexin | 0.8012 | 0.8511 | 0.7847 | 1.2997 | 1.4255 | 1.2582 | 1.3278 | 0.6823 | 0.6508 | 0.2856 |
| Fangchinoline | 0.8291 | 0.8106 | 0.7938 | 1.3701 | 1.3234 | 1.2812 | 1.3249 | 0.6823 | 0.6508 | 0.2856 |
| Harpagoside | 0.8169 | 0.8004 | 0.8106 | 1.3393 | 1.2977 | 1.3234 | 1.3201 | 0.6823 | 0.6508 | 0.2856 |
| Carmofur | 0.7952 | 0.8292 | 0.8033 | 1.2846 | 1.3703 | 1.3049 | 1.3199 | 0.6823 | 0.6508 | 0.2856 |
| Dihydrocapsaicin | 0.8313 | 0.7934 | 0.8017 | 1.3756 | 1.2801 | 1.3011 | 1.3189 | 0.6823 | 0.6508 | 0.2856 |
| Hesperidin | 0.8234 | 0.8060 | 0.7969 | 1.3557 | 1.3118 | 1.2888 | 1.3188 | 0.6823 | 0.6508 | 0.2856 |
| Antineoplaston A10 | 0.8455 | 0.7903 | 0.7893 | 1.4114 | 1.2722 | 1.2698 | 1.3178 | 0.6823 | 0.6508 | 0.2856 |
| Glycyrrhizin (NSC 167409) | 0.8069 | 0.7943 | 0.8174 | 1.3141 | 1.2823 | 1.3406 | 1.3124 | 0.6823 | 0.6508 | 0.2856 |
| Belinostat (PXD101) | 0.8553 | 0.7233 | 0.8392 | 1.4361 | 1.1034 | 1.3955 | 1.3116 | 0.6823 | 0.6508 | 0.2856 |
| Kaempferol (NSC 407289) | 0.8484 | 0.7898 | 0.7767 | 1.4187 | 1.2710 | 1.2381 | 1.3093 | 0.6823 | 0.6508 | 0.2856 |
| Alpelisib (BYL719) | 0.8009 | 0.8187 | 0.7952 | 1.2990 | 1.3438 | 1.2847 | 1.3092 | 0.6823 | 0.6508 | 0.2856 |
| Fargesin | 0.7785 | 0.8450 | 0.7858 | 1.2425 | 1.4101 | 1.2610 | 1.3045 | 0.6823 | 0.6508 | 0.2856 |
| Pipobroman | 0.8029 | 0.8094 | 0.7958 | 1.3040 | 1.3204 | 1.2862 | 1.3035 | 0.6823 | 0.6508 | 0.2856 |
| Rosmarinic acid | 0.7570 | 0.8557 | 0.7871 | 1.1883 | 1.4371 | 1.2642 | 1.2965 | 0.6823 | 0.6508 | 0.2856 |
| Masitinib (AB1010) | 0.8019 | 0.8116 | 0.7862 | 1.3015 | 1.3259 | 1.2618 | 1.2964 | 0.6823 | 0.6508 | 0.2856 |
| Tirapazamine | 0.8225 | 0.7925 | 0.7837 | 1.3534 | 1.2778 | 1.2557 | 1.2956 | 0.6823 | 0.6508 | 0.2856 |
| Betaine | 0.7875 | 0.8459 | 0.7648 | 1.2652 | 1.4124 | 1.2080 | 1.2952 | 0.6823 | 0.6508 | 0.2856 |
| Sodium ferulate | 0.8369 | 0.6629 | 0.7755 | 1.5096 | 1.0331 | 1.3415 | 1.2947 | 0.6823 | 0.6508 | 0.2856 |
| Methylprednisolone (NSC-19987) | 0.7794 | 0.8009 | 0.8121 | 1.2448 | 1.2990 | 1.3271 | 1.2903 | 0.6823 | 0.6508 | 0.2856 |
| Tanshinone I | 0.8302 | 0.7815 | 0.7784 | 1.3728 | 1.2501 | 1.2421 | 1.2883 | 0.6823 | 0.6508 | 0.2856 |
| Fluzoparib (SHR-3162) | 0.7986 | 0.7837 | 0.8061 | 1.2932 | 1.2556 | 1.3121 | 1.2870 | 0.6823 | 0.6508 | 0.2856 |
| Gossypol Acetate | 0.7913 | 0.8220 | 0.7735 | 1.2748 | 1.3522 | 1.2299 | 1.2856 | 0.6823 | 0.6508 | 0.2856 |
| Megestrol Acetate | 0.7955 | 0.8088 | 0.7823 | 1.2854 | 1.3189 | 1.2522 | 1.2855 | 0.6823 | 0.6508 | 0.2856 |
| Bazedoxifene (TSE-424) acetate | 0.7927 | 0.8047 | 0.7864 | 1.2783 | 1.3086 | 1.2624 | 1.2831 | 0.6823 | 0.6508 | 0.2856 |
| Tamoxifen (ICI 46474) Citrate | 0.7822 | 0.7967 | 0.8020 | 1.2518 | 1.2884 | 1.3016 | 1.2806 | 0.6823 | 0.6508 | 0.2856 |
| Olaparib (AZD2281) | 0.7397 | 0.8368 | 0.7996 | 1.1447 | 1.3895 | 1.2958 | 1.2767 | 0.6823 | 0.6508 | 0.2856 |
| Quercetin Dihydrate | 0.8363 | 0.7368 | 0.8002 | 1.3882 | 1.1374 | 1.2973 | 1.2743 | 0.6823 | 0.6508 | 0.2856 |
| Pimethixene maleate | 0.8021 | 0.7764 | 0.7940 | 1.3020 | 1.2372 | 1.2814 | 1.2735 | 0.6823 | 0.6508 | 0.2856 |
| Resveratrol (SRT501) | 0.8082 | 0.7531 | 0.8103 | 1.3174 | 1.1785 | 1.3228 | 1.2729 | 0.6823 | 0.6508 | 0.2856 |
| Vismodegib (GDC-0449) | 0.8149 | 0.7156 | 0.8382 | 1.3343 | 1.0839 | 1.3929 | 1.2704 | 0.6823 | 0.6508 | 0.2856 |
| Tanshinone IIA | 0.7679 | 0.8293 | 0.7714 | 1.2158 | 1.3706 | 1.2246 | 1.2703 | 0.6823 | 0.6508 | 0.2856 |
| Enasidenib Mesylate | 0.8024 | 0.7704 | 0.7937 | 1.3027 | 1.2221 | 1.2808 | 1.2686 | 0.6823 | 0.6508 | 0.2856 |
| Hydroxyurea (NSC-32065) | 0.7749 | 0.7876 | 0.8021 | 1.2334 | 1.2654 | 1.3021 | 1.2670 | 0.6823 | 0.6508 | 0.2856 |
| Capsaicin(Vanilloid) | 0.7721 | 0.8083 | 0.7826 | 1.2264 | 1.3176 | 1.2529 | 1.2656 | 0.6823 | 0.6508 | 0.2856 |
| Verteporfin (CL 318952) | 0.7590 | 0.8086 | 0.7942 | 1.1933 | 1.3184 | 1.2821 | 1.2646 | 0.6823 | 0.6508 | 0.2856 |
| Alectinib (CH5424802) | 0.7777 | 0.8108 | 0.7696 | 1.2405 | 1.3239 | 1.2200 | 1.2615 | 0.6823 | 0.6508 | 0.2856 |
| Sinomenine | 0.7867 | 0.8025 | 0.7674 | 1.2632 | 1.3030 | 1.2144 | 1.2602 | 0.6823 | 0.6508 | 0.2856 |
| Magnolol | 0.8027 | 0.7879 | 0.7635 | 1.3035 | 1.2662 | 1.2047 | 1.2581 | 0.6823 | 0.6508 | 0.2856 |
| Estramustine phosphate sodium | 0.7657 | 0.7615 | 0.7057 | 1.3146 | 1.3031 | 1.1504 | 1.2560 | 0.6823 | 0.6508 | 0.2856 |
| Asiaticoside | 0.8034 | 0.7845 | 0.7629 | 1.3053 | 1.2576 | 1.2031 | 1.2553 | 0.6823 | 0.6508 | 0.2856 |
| Bismuth Subcitrate Potassium | 0.7323 | 0.7685 | 0.7274 | 1.2232 | 1.3223 | 1.2097 | 1.2517 | 0.6823 | 0.6508 | 0.2856 |
| Daidzin | 0.7580 | 0.8471 | 0.7399 | 1.1908 | 1.4154 | 1.1451 | 1.2504 | 0.6823 | 0.6508 | 0.2856 |
| Mercaptopurine (6-MP) | 0.8680 | 0.6804 | 0.7957 | 1.4681 | 0.9952 | 1.2859 | 1.2498 | 0.6823 | 0.6508 | 0.2856 |
| Phenethyl alcohol | 0.8271 | 0.7679 | 0.7490 | 1.3650 | 1.2158 | 1.1682 | 1.2497 | 0.6823 | 0.6508 | 0.2856 |
| Dehydroandrographolide Succinate Potasium Salt | 0.8057 | 0.7588 | 0.7782 | 1.3111 | 1.1928 | 1.2417 | 1.2485 | 0.6823 | 0.6508 | 0.2856 |
| Ferulic Acid | 0.8070 | 0.7666 | 0.7576 | 1.3143 | 1.2125 | 1.1899 | 1.2389 | 0.6823 | 0.6508 | 0.2856 |
| Pazopanib | 0.8250 | 0.7031 | 0.7969 | 1.3597 | 1.0524 | 1.2890 | 1.2337 | 0.6823 | 0.6508 | 0.2856 |
| Mitotane | 0.7906 | 0.7462 | 0.7881 | 1.2730 | 1.1611 | 1.2667 | 1.2336 | 0.6823 | 0.6508 | 0.2856 |
| Bicalutamide (ICI-176334) | 0.7758 | 0.8099 | 0.7390 | 1.2357 | 1.3217 | 1.1431 | 1.2335 | 0.6823 | 0.6508 | 0.2856 |
| Curcumin | 0.7751 | 0.7637 | 0.7759 | 1.2339 | 1.2052 | 1.2359 | 1.2250 | 0.6823 | 0.6508 | 0.2856 |
| 2-Methoxyestradiol (2-MeOE2) | 0.7139 | 0.8153 | 0.7851 | 1.0797 | 1.3353 | 1.2591 | 1.2247 | 0.6823 | 0.6508 | 0.2856 |
| Metyrosine | 0.8037 | 0.6869 | 0.7064 | 1.4187 | 1.0988 | 1.1524 | 1.2233 | 0.6823 | 0.6508 | 0.2856 |
| Thiotepa | 0.7628 | 0.7699 | 0.7799 | 1.2029 | 1.2208 | 1.2459 | 1.2232 | 0.6823 | 0.6508 | 0.2856 |
| Cytidine | 0.7666 | 0.7691 | 0.7761 | 1.2125 | 1.2188 | 1.2365 | 1.2226 | 0.6823 | 0.6508 | 0.2856 |
| Tegafur (FT-207) | 0.7809 | 0.7588 | 0.7721 | 1.2486 | 1.1928 | 1.2264 | 1.2226 | 0.6823 | 0.6508 | 0.2856 |
| Varenicline Tartrate (CP 526555-18) | 0.7119 | 0.7682 | 0.7145 | 1.1673 | 1.3215 | 1.1744 | 1.2211 | 0.6823 | 0.6508 | 0.2856 |
| Phenoxybenzamine HCl | 0.7564 | 0.8365 | 0.7159 | 1.1868 | 1.3887 | 1.0848 | 1.2201 | 0.6823 | 0.6508 | 0.2856 |
| Treosulfan | 0.7593 | 0.8123 | 0.7367 | 1.1941 | 1.3277 | 1.1372 | 1.2197 | 0.6823 | 0.6508 | 0.2856 |
| Corydaline | 0.7103 | 0.8129 | 0.7847 | 1.0706 | 1.3292 | 1.2582 | 1.2193 | 0.6823 | 0.6508 | 0.2856 |
| Salicin | 0.7570 | 0.8086 | 0.7419 | 1.1883 | 1.3184 | 1.1503 | 1.2190 | 0.6823 | 0.6508 | 0.2856 |
| Sorafenib (BAY 43-9006) tosylate | 0.6979 | 0.8782 | 0.7280 | 1.0393 | 1.4938 | 1.1153 | 1.2162 | 0.6823 | 0.6508 | 0.2856 |
| Nintedanib (BIBF 1120) | 0.8207 | 0.7069 | 0.7748 | 1.3489 | 1.0620 | 1.2332 | 1.2147 | 0.6823 | 0.6508 | 0.2856 |
| (+)-Fangchinoline | 0.8856 | 0.6964 | 0.7182 | 1.5125 | 1.0355 | 1.0906 | 1.2129 | 0.6823 | 0.6508 | 0.2856 |
| Vanillin | 0.7580 | 0.7862 | 0.7494 | 1.1908 | 1.2619 | 1.1691 | 1.2073 | 0.6823 | 0.6508 | 0.2856 |
| Cinnamaldehyde | 0.7010 | 0.8459 | 0.7449 | 1.0471 | 1.4124 | 1.1577 | 1.2058 | 0.6823 | 0.6508 | 0.2856 |
| Toremifene Citrate (NK 622) | 0.7578 | 0.7616 | 0.7723 | 1.1903 | 1.1999 | 1.2269 | 1.2057 | 0.6823 | 0.6508 | 0.2856 |
| Apatinib | 0.7653 | 0.7461 | 0.7726 | 1.2092 | 1.1608 | 1.2276 | 1.1992 | 0.6823 | 0.6508 | 0.2856 |
| Ifosfamide (NSC109724) | 0.7645 | 0.7759 | 0.7433 | 1.2072 | 1.2359 | 1.1537 | 1.1989 | 0.6823 | 0.6508 | 0.2856 |
| Hydrocortisone sodium succinate | 0.8124 | 0.6978 | 0.7719 | 1.3280 | 1.0391 | 1.2259 | 1.1976 | 0.6823 | 0.6508 | 0.2856 |
| Bexarotene (LGD1069) | 0.7549 | 0.7557 | 0.7712 | 1.1830 | 1.1850 | 1.2241 | 1.1974 | 0.6823 | 0.6508 | 0.2856 |
| Darunavir Ethanolate | 0.6214 | 0.8739 | 0.7852 | 0.8465 | 1.4830 | 1.2595 | 1.1963 | 0.6823 | 0.6508 | 0.2856 |
| Metformin HCl | 0.7873 | 0.7274 | 0.7625 | 1.2647 | 1.1137 | 1.2023 | 1.1936 | 0.6823 | 0.6508 | 0.2856 |
| Sodium Demethylcantharidate | 0.7472 | 0.7124 | 0.7037 | 1.2640 | 1.1687 | 1.1450 | 1.1925 | 0.6823 | 0.6508 | 0.2856 |
| Gimeracil | 0.7804 | 0.7220 | 0.7714 | 1.2473 | 1.1001 | 1.2246 | 1.1906 | 0.6823 | 0.6508 | 0.2856 |
| Clodronate Disodium | 0.6861 | 0.7557 | 0.7184 | 1.0967 | 1.2872 | 1.1850 | 1.1896 | 0.6823 | 0.6508 | 0.2856 |
| Piperine | 0.7594 | 0.7958 | 0.7163 | 1.1944 | 1.2861 | 1.0857 | 1.1887 | 0.6823 | 0.6508 | 0.2856 |
| Leucovorin (NSC -3590) Calcium Pentahydrate | 0.6915 | 0.7592 | 0.7035 | 1.1114 | 1.2968 | 1.1443 | 1.1842 | 0.6823 | 0.6508 | 0.2856 |
| Germacrone | 0.6452 | 0.8203 | 0.7974 | 0.9065 | 1.3479 | 1.2902 | 1.1815 | 0.6823 | 0.6508 | 0.2856 |
| Regorafenib Hydrochloride | 0.7483 | 0.7527 | 0.7595 | 1.1664 | 1.1775 | 1.1947 | 1.1795 | 0.6823 | 0.6508 | 0.2856 |
| Imiquimod (R-837) | 0.7700 | 0.7679 | 0.7172 | 1.2211 | 1.2158 | 1.0881 | 1.1750 | 0.6823 | 0.6508 | 0.2856 |
| Pentostatin | 0.7734 | 0.7972 | 0.6837 | 1.2296 | 1.2896 | 1.0036 | 1.1743 | 0.6823 | 0.6508 | 0.2856 |
| Anagrelide HCl | 0.8128 | 0.7223 | 0.7189 | 1.3290 | 1.1008 | 1.0923 | 1.1740 | 0.6823 | 0.6508 | 0.2856 |
| Puerarin | 0.8146 | 0.7273 | 0.7117 | 1.3335 | 1.1134 | 1.0742 | 1.1737 | 0.6823 | 0.6508 | 0.2856 |
| Honokiol (NSC 293100) | 0.7937 | 0.7204 | 0.7390 | 1.2808 | 1.0960 | 1.1430 | 1.1733 | 0.6823 | 0.6508 | 0.2856 |
| Norcantharidin | 0.7614 | 0.7173 | 0.7738 | 1.1994 | 1.0882 | 1.2306 | 1.1727 | 0.6823 | 0.6508 | 0.2856 |
| Bornyl acetate | 0.7562 | 0.7570 | 0.7384 | 1.1863 | 1.1883 | 1.1413 | 1.1720 | 0.6823 | 0.6508 | 0.2856 |
| Thymopentin | 0.7273 | 0.8148 | 0.7084 | 1.1134 | 1.3340 | 1.0657 | 1.1710 | 0.6823 | 0.6508 | 0.2856 |
| Nilotinib hydrochloride monohydrate | 0.7544 | 0.7512 | 0.7440 | 1.1817 | 1.1737 | 1.1556 | 1.1703 | 0.6823 | 0.6508 | 0.2856 |
| Busulfan (NSC-750) | 0.7159 | 0.7953 | 0.7362 | 1.0847 | 1.2849 | 1.1359 | 1.1685 | 0.6823 | 0.6508 | 0.2856 |
| Lenvatinib (E7080) | 0.7918 | 0.7727 | 0.6823 | 1.2760 | 1.2279 | 1.0000 | 1.1680 | 0.6823 | 0.6508 | 0.2856 |
| Entinostat (MS-275) | 0.6180 | 0.8408 | 0.7853 | 0.8379 | 1.3995 | 1.2596 | 1.1657 | 0.6823 | 0.6508 | 0.2856 |
| MPEP | 0.7237 | 0.8092 | 0.7094 | 1.1044 | 1.3199 | 1.0682 | 1.1642 | 0.6823 | 0.6508 | 0.2856 |
| Niraparib (MK-4827) | 0.7665 | 0.7454 | 0.7264 | 1.2123 | 1.1591 | 1.1111 | 1.1608 | 0.6823 | 0.6508 | 0.2856 |
| Carmustine | 0.7728 | 0.7068 | 0.7578 | 1.2281 | 1.0618 | 1.1904 | 1.1601 | 0.6823 | 0.6508 | 0.2856 |
| Pexidartinib (PLX3397) | 0.6617 | 0.8002 | 0.7738 | 0.9481 | 1.2972 | 1.2306 | 1.1586 | 0.6823 | 0.6508 | 0.2856 |
| Clodronate disodium tetrahydrate | 0.7667 | 0.6136 | 0.7458 | 1.3174 | 0.8981 | 1.2602 | 1.1586 | 0.6823 | 0.6508 | 0.2856 |
| Cyclocytidine HCl | 0.7691 | 0.7364 | 0.7280 | 1.2188 | 1.1364 | 1.1153 | 1.1568 | 0.6823 | 0.6508 | 0.2856 |
| Diethylstilbestrol | 0.7183 | 0.7766 | 0.7385 | 1.0907 | 1.2377 | 1.1417 | 1.1567 | 0.6823 | 0.6508 | 0.2856 |
| Pemetrexed (LY-231514) disodium | 0.6772 | 0.7849 | 0.6606 | 1.0723 | 1.3672 | 1.0267 | 1.1554 | 0.6823 | 0.6508 | 0.2856 |
| Oleanolic Acid | 0.7622 | 0.7088 | 0.7591 | 1.2014 | 1.0668 | 1.1937 | 1.1540 | 0.6823 | 0.6508 | 0.2856 |
| Sophoridine | 0.7702 | 0.7481 | 0.7073 | 1.2216 | 1.1659 | 1.0631 | 1.1502 | 0.6823 | 0.6508 | 0.2856 |
| Isotretinoin | 0.7532 | 0.7256 | 0.7466 | 1.1787 | 1.1092 | 1.1620 | 1.1500 | 0.6823 | 0.6508 | 0.2856 |
| Silymarin | 0.7593 | 0.7438 | 0.7219 | 1.1941 | 1.1550 | 1.0999 | 1.1497 | 0.6823 | 0.6508 | 0.2856 |
| Orotic acid (6-Carboxyuracil) | 0.7806 | 0.7358 | 0.7082 | 1.2478 | 1.1349 | 1.0652 | 1.1493 | 0.6823 | 0.6508 | 0.2856 |
| Danshensu | 0.7540 | 0.6763 | 0.6838 | 1.2826 | 1.0698 | 1.0904 | 1.1476 | 0.6823 | 0.6508 | 0.2856 |
| Fosaprepitant dimeglumine salt | 0.8001 | 0.7017 | 0.7184 | 1.2969 | 1.0489 | 1.0909 | 1.1456 | 0.6823 | 0.6508 | 0.2856 |
| Fenretinide (NSC 374551) | 0.7681 | 0.7081 | 0.7417 | 1.2163 | 1.0650 | 1.1497 | 1.1437 | 0.6823 | 0.6508 | 0.2856 |
| Fumaric acid | 0.7971 | 0.7326 | 0.6859 | 1.2894 | 1.1268 | 1.0092 | 1.1418 | 0.6823 | 0.6508 | 0.2856 |
| Ammonium Glycyrrhizinate | 0.7381 | 0.7676 | 0.7074 | 1.1407 | 1.2150 | 1.0632 | 1.1396 | 0.6823 | 0.6508 | 0.2856 |
| (±)-α-Bisabolol | 0.6898 | 0.7444 | 0.7782 | 1.0189 | 1.1565 | 1.2418 | 1.1391 | 0.6823 | 0.6508 | 0.2856 |
| Rucaparib | 0.7448 | 0.7595 | 0.7064 | 1.1575 | 1.1946 | 1.0607 | 1.1376 | 0.6823 | 0.6508 | 0.2856 |
| Regorafenib (BAY 73-4506) | 0.7850 | 0.7126 | 0.7126 | 1.2589 | 1.0764 | 1.0764 | 1.1372 | 0.6823 | 0.6508 | 0.2856 |
| Tiaprofenic acid | 0.7958 | 0.7295 | 0.6848 | 1.2861 | 1.1190 | 1.0063 | 1.1371 | 0.6823 | 0.6508 | 0.2856 |
| Medroxyprogesterone acetate (NSC-26386) | 0.7287 | 0.7715 | 0.7089 | 1.1170 | 1.2249 | 1.0671 | 1.1363 | 0.6823 | 0.6508 | 0.2856 |
| Palbociclib (PD-0332991) HCl | 0.6611 | 0.7524 | 0.6870 | 1.0282 | 1.2782 | 1.0992 | 1.1352 | 0.6823 | 0.6508 | 0.2856 |
| Morin Hydrate | 0.7893 | 0.7352 | 0.6833 | 1.2697 | 1.1334 | 1.0025 | 1.1352 | 0.6823 | 0.6508 | 0.2856 |
| Allopurinol | 0.7553 | 0.7491 | 0.7032 | 1.1840 | 1.1684 | 1.0528 | 1.1351 | 0.6823 | 0.6508 | 0.2856 |
| Formononetin | 0.7845 | 0.6241 | 0.7975 | 1.2576 | 0.8533 | 1.2905 | 1.1338 | 0.6823 | 0.6508 | 0.2856 |
| Cinnamic acid | 0.7202 | 0.7785 | 0.7068 | 1.0955 | 1.2425 | 1.0618 | 1.1333 | 0.6823 | 0.6508 | 0.2856 |
| Rutaecarpine | 0.8127 | 0.7107 | 0.6820 | 1.3287 | 1.0716 | 0.9992 | 1.1332 | 0.6823 | 0.6508 | 0.2856 |
| Estradiol valerate | 0.7852 | 0.6557 | 0.7639 | 1.2594 | 0.9329 | 1.2056 | 1.1327 | 0.6823 | 0.6508 | 0.2856 |
| Ursolic Acid | 0.7163 | 0.7782 | 0.7085 | 1.0857 | 1.2417 | 1.0661 | 1.1312 | 0.6823 | 0.6508 | 0.2856 |
| Azacitidine (5-Azacytidine) | 0.7242 | 0.7760 | 0.7013 | 1.1056 | 1.2362 | 1.0480 | 1.1299 | 0.6823 | 0.6508 | 0.2856 |
| Lonidamine (AF-1890) | 0.7601 | 0.7292 | 0.7077 | 1.1961 | 1.1182 | 1.0640 | 1.1261 | 0.6823 | 0.6508 | 0.2856 |
| Enzastaurin (LY317615) | 0.7823 | 0.6974 | 0.7161 | 1.2521 | 1.0381 | 1.0853 | 1.1252 | 0.6823 | 0.6508 | 0.2856 |
| Tipiracil hydrochloride | 0.7810 | 0.6971 | 0.7168 | 1.2488 | 1.0373 | 1.0871 | 1.1244 | 0.6823 | 0.6508 | 0.2856 |
| Rucaparib (AG-014699) phosphate | 0.5969 | 0.8390 | 0.7582 | 0.7847 | 1.3950 | 1.1915 | 1.1237 | 0.6823 | 0.6508 | 0.2856 |
| Anastrozole (ZD-1033) | 0.7550 | 0.7229 | 0.7162 | 1.1833 | 1.1023 | 1.0856 | 1.1237 | 0.6823 | 0.6508 | 0.2856 |
| (+)-Matrine | 0.7291 | 0.7587 | 0.7054 | 1.1180 | 1.1926 | 1.0583 | 1.1230 | 0.6823 | 0.6508 | 0.2856 |
| Imatinib (STI571) Mesylate | 0.5863 | 0.8483 | 0.7575 | 0.7580 | 1.4185 | 1.1895 | 1.1220 | 0.6823 | 0.6508 | 0.2856 |
| (S)-(−)-Limonene | 0.7317 | 0.7511 | 0.7090 | 1.1245 | 1.1734 | 1.0674 | 1.1218 | 0.6823 | 0.6508 | 0.2856 |
| Almonertinib (HS-10296) | 0.7174 | 0.7694 | 0.7048 | 1.0885 | 1.2196 | 1.0567 | 1.1216 | 0.6823 | 0.6508 | 0.2856 |
| Tretinoin (NSC 122758) | 0.7390 | 0.7414 | 0.7104 | 1.1429 | 1.1490 | 1.0708 | 1.1209 | 0.6823 | 0.6508 | 0.2856 |
| Flutamide | 0.7242 | 0.7691 | 0.6974 | 1.1056 | 1.2188 | 1.0379 | 1.1208 | 0.6823 | 0.6508 | 0.2856 |
| Osimertinib mesylate | 0.7793 | 0.7182 | 0.6927 | 1.2445 | 1.0905 | 1.0263 | 1.1204 | 0.6823 | 0.6508 | 0.2856 |
| Quercetin (NSC 9221) | 0.6817 | 0.7993 | 0.7075 | 0.9985 | 1.2949 | 1.0634 | 1.1189 | 0.6823 | 0.6508 | 0.2856 |
| Chlorobutanol | 0.7648 | 0.7141 | 0.7067 | 1.2080 | 1.0802 | 1.0616 | 1.1166 | 0.6823 | 0.6508 | 0.2856 |
| Berbamine | 0.7231 | 0.7257 | 0.7348 | 1.1028 | 1.1094 | 1.1323 | 1.1148 | 0.6823 | 0.6508 | 0.2856 |
| Irbesartan | 0.7417 | 0.7280 | 0.7064 | 1.1497 | 1.1152 | 1.0608 | 1.1086 | 0.6823 | 0.6508 | 0.2856 |
| Valrubicin | 0.7032 | 0.7667 | 0.7058 | 1.0527 | 1.2128 | 1.0593 | 1.1083 | 0.6823 | 0.6508 | 0.2856 |
| Bazedoxifene (WAY-140424) HCl | 0.7471 | 0.7161 | 0.7118 | 1.1633 | 1.0852 | 1.0744 | 1.1077 | 0.6823 | 0.6508 | 0.2856 |
| Kojic acid | 0.7789 | 0.6895 | 0.6993 | 1.2435 | 1.0181 | 1.0429 | 1.1015 | 0.6823 | 0.6508 | 0.2856 |
| Epalrestat (ONO-2235) | 0.7339 | 0.7263 | 0.7071 | 1.1301 | 1.1109 | 1.0626 | 1.1012 | 0.6823 | 0.6508 | 0.2856 |
| Gefitinib hydrochloride | 0.6926 | 0.7460 | 0.7258 | 1.0260 | 1.1606 | 1.1096 | 1.0987 | 0.6823 | 0.6508 | 0.2856 |
| Vorinostat (SAHA) | 0.6018 | 0.8481 | 0.7139 | 0.7971 | 1.4179 | 1.0795 | 1.0982 | 0.6823 | 0.6508 | 0.2856 |
| Meprednisone | 0.6992 | 0.7401 | 0.7237 | 1.0426 | 1.1457 | 1.1042 | 1.0975 | 0.6823 | 0.6508 | 0.2856 |
| Rimiducid (AP1903) | 0.7126 | 0.7442 | 0.7047 | 1.0764 | 1.1560 | 1.0565 | 1.0963 | 0.6823 | 0.6508 | 0.2856 |
| Capmatinib (INCB28060) | 0.7368 | 0.7081 | 0.7158 | 1.1374 | 1.0650 | 1.0845 | 1.0956 | 0.6823 | 0.6508 | 0.2856 |
| Valemetostat (DS-3201) | 0.7957 | 0.6261 | 0.7379 | 1.2859 | 0.8583 | 1.1402 | 1.0948 | 0.6823 | 0.6508 | 0.2856 |
| Selpercatinib (LOXO-292) | 0.7227 | 0.7346 | 0.7012 | 1.1018 | 1.1318 | 1.0476 | 1.0938 | 0.6823 | 0.6508 | 0.2856 |
| Lynestrenol | 0.7501 | 0.6832 | 0.7248 | 1.1709 | 1.0023 | 1.1071 | 1.0934 | 0.6823 | 0.6508 | 0.2856 |
| Sunitinib (SU11248) malate | 0.6399 | 0.7789 | 0.7368 | 0.8931 | 1.2435 | 1.1374 | 1.0913 | 0.6823 | 0.6508 | 0.2856 |
| TAS-102 | 0.7782 | 0.6702 | 0.7058 | 1.2417 | 0.9695 | 1.0593 | 1.0902 | 0.6823 | 0.6508 | 0.2856 |
| Gefitinib (ZD1839) | 0.7083 | 0.7199 | 0.7245 | 1.0655 | 1.0948 | 1.1064 | 1.0889 | 0.6823 | 0.6508 | 0.2856 |
| Doxifluridine | 0.7393 | 0.7091 | 0.7042 | 1.1437 | 1.0676 | 1.0552 | 1.0888 | 0.6823 | 0.6508 | 0.2856 |
| Omeprazole | 0.7777 | 0.6346 | 0.7357 | 1.2405 | 0.8798 | 1.1346 | 1.0849 | 0.6823 | 0.6508 | 0.2856 |
| Daidzein | 0.7292 | 0.6951 | 0.7214 | 1.1182 | 1.0323 | 1.0985 | 1.0830 | 0.6823 | 0.6508 | 0.2856 |
| Streptozotocin (STZ) | 0.7311 | 0.7119 | 0.7015 | 1.1230 | 1.0746 | 1.0483 | 1.0820 | 0.6823 | 0.6508 | 0.2856 |
| Lapatinib (GW-572016) | 0.7257 | 0.7171 | 0.7015 | 1.1094 | 1.0877 | 1.0484 | 1.0819 | 0.6823 | 0.6508 | 0.2856 |
| Berberine | 0.7620 | 0.6727 | 0.7058 | 1.2009 | 0.9758 | 1.0593 | 1.0787 | 0.6823 | 0.6508 | 0.2856 |
| Indirubin (NSC 105327) | 0.6768 | 0.8133 | 0.6493 | 0.9861 | 1.3302 | 0.9169 | 1.0777 | 0.6823 | 0.6508 | 0.2856 |
| Carbendazim | 0.6708 | 0.7791 | 0.6893 | 0.9710 | 1.2440 | 1.0176 | 1.0775 | 0.6823 | 0.6508 | 0.2856 |
| Gemcitabine (LY-188011) | 0.6735 | 0.7614 | 0.7033 | 0.9778 | 1.1994 | 1.0530 | 1.0767 | 0.6823 | 0.6508 | 0.2856 |
| Bleomycin (NSC125066) sulfate | 0.7651 | 0.6675 | 0.7039 | 1.2087 | 0.9627 | 1.0544 | 1.0753 | 0.6823 | 0.6508 | 0.2856 |
| Aristolochic acid A | 0.6944 | 0.7595 | 0.6826 | 1.0305 | 1.1946 | 1.0007 | 1.0753 | 0.6823 | 0.6508 | 0.2856 |
| Phytol | 0.7439 | 0.7059 | 0.6838 | 1.1553 | 1.0595 | 1.0038 | 1.0728 | 0.6823 | 0.6508 | 0.2856 |
| Ketorolac | 0.6975 | 0.7187 | 0.7173 | 1.0383 | 1.0918 | 1.0882 | 1.0728 | 0.6823 | 0.6508 | 0.2856 |
| Sodium Aescinate | 0.7144 | 0.7209 | 0.6980 | 1.0809 | 1.0973 | 1.0397 | 1.0726 | 0.6823 | 0.6508 | 0.2856 |
| Isofraxidin | 0.6510 | 0.7920 | 0.6838 | 0.9211 | 1.2765 | 1.0038 | 1.0671 | 0.6823 | 0.6508 | 0.2856 |
| Prednisone (NSC-10023) | 0.7098 | 0.6998 | 0.7164 | 1.0693 | 1.0441 | 1.0859 | 1.0664 | 0.6823 | 0.6508 | 0.2856 |
| Betamethasone | 0.6010 | 0.7920 | 0.7328 | 0.7951 | 1.2765 | 1.1273 | 1.0663 | 0.6823 | 0.6508 | 0.2856 |
| Isorhamnetin | 0.6829 | 0.7800 | 0.6629 | 1.0015 | 1.2463 | 0.9510 | 1.0663 | 0.6823 | 0.6508 | 0.2856 |
| Pazopanib HCl (GW786034 HCl) | 0.6866 | 0.7966 | 0.6425 | 1.0108 | 1.2881 | 0.8997 | 1.0662 | 0.6823 | 0.6508 | 0.2856 |
| Sclareol | 0.6919 | 0.7294 | 0.7020 | 1.0242 | 1.1187 | 1.0496 | 1.0642 | 0.6823 | 0.6508 | 0.2856 |
| Nintedanib Ethanesulfonate Salt | 0.7706 | 0.6891 | 0.6626 | 1.2226 | 1.0171 | 0.9505 | 1.0634 | 0.6823 | 0.6508 | 0.2856 |
| Proanthocyanidins | 0.7098 | 0.6972 | 0.7148 | 1.0693 | 1.0376 | 1.0819 | 1.0629 | 0.6823 | 0.6508 | 0.2856 |
| Sodium Houttuyfonate | 0.6734 | 0.7294 | 0.7176 | 0.9776 | 1.1187 | 1.0891 | 1.0618 | 0.6823 | 0.6508 | 0.2856 |
| Masitinib mesylate | 0.7576 | 0.6460 | 0.7164 | 1.1898 | 0.9085 | 1.0859 | 1.0614 | 0.6823 | 0.6508 | 0.2856 |
| Trametinib DMSO solvate | 0.7017 | 0.6986 | 0.7195 | 1.0489 | 1.0411 | 1.0938 | 1.0613 | 0.6823 | 0.6508 | 0.2856 |
| Fludarabine (NSC 118218) | 0.7117 | 0.6897 | 0.7173 | 1.0741 | 1.0187 | 1.0882 | 1.0603 | 0.6823 | 0.6508 | 0.2856 |
| Cyproterone Acetate | 0.7394 | 0.6595 | 0.7193 | 1.1439 | 0.9425 | 1.0931 | 1.0599 | 0.6823 | 0.6508 | 0.2856 |
| Usnic acid | 0.7456 | 0.6999 | 0.6714 | 1.1596 | 1.0444 | 0.9725 | 1.0588 | 0.6823 | 0.6508 | 0.2856 |
| Tanshinone IIA sulfonate sodium | 0.7383 | 0.6908 | 0.6866 | 1.1412 | 1.0214 | 1.0107 | 1.0578 | 0.6823 | 0.6508 | 0.2856 |
| Gemcitabine (LY-188011) HCl | 0.6195 | 0.7385 | 0.6548 | 0.9143 | 1.2401 | 1.0109 | 1.0551 | 0.6823 | 0.6508 | 0.2856 |
| Loganin | 0.7602 | 0.6620 | 0.6890 | 1.1964 | 0.9488 | 1.0169 | 1.0540 | 0.6823 | 0.6508 | 0.2856 |
| Thioguanine (NSC 752) | 0.7129 | 0.7010 | 0.6972 | 1.0771 | 1.0471 | 1.0377 | 1.0540 | 0.6823 | 0.6508 | 0.2856 |
| Sanguinarine | 0.7142 | 0.7004 | 0.6959 | 1.0804 | 1.0456 | 1.0342 | 1.0534 | 0.6823 | 0.6508 | 0.2856 |
| Trigonelline Hydrochloride | 0.6792 | 0.7425 | 0.6880 | 0.9922 | 1.1518 | 1.0144 | 1.0528 | 0.6823 | 0.6508 | 0.2856 |
| Berbamine dihydrochloride | 0.7027 | 0.7132 | 0.6938 | 1.0514 | 1.0779 | 1.0290 | 1.0528 | 0.6823 | 0.6508 | 0.2856 |
| Oxtriphylline | 0.6108 | 0.8118 | 0.6857 | 0.8198 | 1.3264 | 1.0086 | 1.0516 | 0.6823 | 0.6508 | 0.2856 |
| Paeonol | 0.7233 | 0.6995 | 0.6836 | 1.1034 | 1.0434 | 1.0033 | 1.0500 | 0.6823 | 0.6508 | 0.2856 |
| Temsirolimus (CCI-779) | 0.6399 | 0.7789 | 0.6872 | 0.8931 | 1.2435 | 1.0124 | 1.0497 | 0.6823 | 0.6508 | 0.2856 |
| L-Theanine | 0.6466 | 0.7011 | 0.6589 | 0.9885 | 1.1377 | 1.0223 | 1.0495 | 0.6823 | 0.6508 | 0.2856 |
| Lentinan | 0.7140 | 0.7086 | 0.6827 | 1.0799 | 1.0663 | 1.0011 | 1.0491 | 0.6823 | 0.6508 | 0.2856 |
| Sinomenine hydrochloride | 0.7356 | 0.6902 | 0.6790 | 1.1344 | 1.0199 | 0.9918 | 1.0487 | 0.6823 | 0.6508 | 0.2856 |
| Anamorelin | 0.6772 | 0.7702 | 0.6549 | 0.9871 | 1.2216 | 0.9308 | 1.0465 | 0.6823 | 0.6508 | 0.2856 |
| Geniposide | 0.7983 | 0.5966 | 0.7064 | 1.2924 | 0.7840 | 1.0608 | 1.0457 | 0.6823 | 0.6508 | 0.2856 |
| Esaxerenone (CS-3150) | 0.7535 | 0.6451 | 0.7019 | 1.1795 | 0.9062 | 1.0494 | 1.0450 | 0.6823 | 0.6508 | 0.2856 |
| Bendamustine (SDX105) HCl | 0.7363 | 0.6600 | 0.7019 | 1.1361 | 0.9438 | 1.0493 | 1.0431 | 0.6823 | 0.6508 | 0.2856 |
| Tazemetostat (EPZ-6438) | 0.6750 | 0.7333 | 0.6885 | 0.9816 | 1.1286 | 1.0156 | 1.0419 | 0.6823 | 0.6508 | 0.2856 |
| Tipifarnib (R115777) | 0.7020 | 0.7113 | 0.6832 | 1.0497 | 1.0731 | 1.0023 | 1.0417 | 0.6823 | 0.6508 | 0.2856 |
| Calcitriol (RO215535) | 0.7323 | 0.6791 | 0.6828 | 1.1260 | 0.9919 | 1.0013 | 1.0397 | 0.6823 | 0.6508 | 0.2856 |
| Teniposide | 0.7664 | 0.6540 | 0.6732 | 1.2120 | 0.9287 | 0.9771 | 1.0392 | 0.6823 | 0.6508 | 0.2856 |
| Astragaloside IV | 0.6646 | 0.7082 | 0.7204 | 0.9554 | 1.0653 | 1.0960 | 1.0389 | 0.6823 | 0.6508 | 0.2856 |
| Congo Red | 0.6833 | 0.7465 | 0.6627 | 1.0025 | 1.1618 | 0.9507 | 1.0383 | 0.6823 | 0.6508 | 0.2856 |
| Infigratinib Phosphate | 0.6717 | 0.7390 | 0.6806 | 0.9733 | 1.1429 | 0.9956 | 1.0373 | 0.6823 | 0.6508 | 0.2856 |
| Naringin Dihydrochalcone | 0.6440 | 0.7564 | 0.6894 | 0.9035 | 1.1868 | 1.0179 | 1.0361 | 0.6823 | 0.6508 | 0.2856 |
| Chlorotrianisene | 0.6605 | 0.7223 | 0.7062 | 0.9450 | 1.1008 | 1.0603 | 1.0354 | 0.6823 | 0.6508 | 0.2856 |
| Flumatinib (HH-GV-678) | 0.7302 | 0.6456 | 0.7090 | 1.1207 | 0.9075 | 1.0674 | 1.0319 | 0.6823 | 0.6508 | 0.2856 |
| Crizotinib (PF-02341066) | 0.6590 | 0.7259 | 0.6939 | 0.9413 | 1.1099 | 1.0294 | 1.0268 | 0.6823 | 0.6508 | 0.2856 |
| Everolimus (RAD001) | 0.5608 | 0.8541 | 0.6629 | 0.6937 | 1.4331 | 0.9510 | 1.0259 | 0.6823 | 0.6508 | 0.2856 |
| 4-Methylumbelliferone (4-MU) | 0.6461 | 0.7238 | 0.7054 | 0.9087 | 1.1046 | 1.0583 | 1.0239 | 0.6823 | 0.6508 | 0.2856 |
| Alectinib (CH5424802) hydrochloride | 0.6784 | 0.6997 | 0.6969 | 0.9902 | 1.0439 | 1.0368 | 1.0236 | 0.6823 | 0.6508 | 0.2856 |
| Medroxyprogesterone | 0.6886 | 0.7100 | 0.6757 | 1.0159 | 1.0698 | 0.9834 | 1.0231 | 0.6823 | 0.6508 | 0.2856 |
| Lapatinib ditosylate monohydrate | 0.6832 | 0.7221 | 0.6683 | 1.0023 | 1.1003 | 0.9648 | 1.0225 | 0.6823 | 0.6508 | 0.2856 |
| Elagolix Sodium | 0.7145 | 0.6889 | 0.6694 | 1.0812 | 1.0166 | 0.9674 | 1.0217 | 0.6823 | 0.6508 | 0.2856 |
| γ-Terpinene | 0.7453 | 0.6417 | 0.6850 | 1.1588 | 0.8977 | 1.0069 | 1.0211 | 0.6823 | 0.6508 | 0.2856 |
| Erlotinib (OSI-774) HCl | 0.6843 | 0.7106 | 0.6763 | 1.0050 | 1.0713 | 0.9849 | 1.0204 | 0.6823 | 0.6508 | 0.2856 |
| Lomustine | 0.6723 | 0.7133 | 0.6854 | 0.9748 | 1.0781 | 1.0078 | 1.0203 | 0.6823 | 0.6508 | 0.2856 |
| Fruquintinib (HMPL-013) | 0.7058 | 0.6930 | 0.6704 | 1.0592 | 1.0270 | 0.9699 | 1.0187 | 0.6823 | 0.6508 | 0.2856 |
| Madecassic acid | 0.6377 | 0.7563 | 0.6750 | 0.8876 | 1.1865 | 0.9815 | 1.0185 | 0.6823 | 0.6508 | 0.2856 |
| Hygromycin B | 0.6049 | 0.7193 | 0.6483 | 0.8743 | 1.1876 | 0.9932 | 1.0184 | 0.6823 | 0.6508 | 0.2856 |
| Ethinyl Estradiol | 0.6752 | 0.7018 | 0.6911 | 0.9821 | 1.0492 | 1.0222 | 1.0178 | 0.6823 | 0.6508 | 0.2856 |
| Palbociclib (PD0332991) Isethionate | 0.6464 | 0.6554 | 0.6690 | 0.9880 | 1.0126 | 1.0499 | 1.0168 | 0.6823 | 0.6508 | 0.2856 |
| Ammonium Glycyrrhizate | 0.6792 | 0.7054 | 0.6812 | 0.9922 | 1.0582 | 0.9971 | 1.0159 | 0.6823 | 0.6508 | 0.2856 |
| Myristic Acid | 0.6539 | 0.7286 | 0.6825 | 0.9284 | 1.1167 | 1.0005 | 1.0152 | 0.6823 | 0.6508 | 0.2856 |
| Erlotinib (OSI-774) | 0.6721 | 0.7087 | 0.6831 | 0.9743 | 1.0665 | 1.0020 | 1.0143 | 0.6823 | 0.6508 | 0.2856 |
| Estradiol dipropionate | 0.6904 | 0.6636 | 0.7083 | 1.0204 | 0.9529 | 1.0656 | 1.0129 | 0.6823 | 0.6508 | 0.2856 |
| Procarbazine HCl | 0.6152 | 0.7159 | 0.6347 | 0.9025 | 1.1783 | 0.9560 | 1.0122 | 0.6823 | 0.6508 | 0.2856 |
| Costunolide | 0.6939 | 0.6667 | 0.7003 | 1.0292 | 0.9607 | 1.0452 | 1.0117 | 0.6823 | 0.6508 | 0.2856 |
| Dabrafenib (GSK2118436) | 0.6627 | 0.7239 | 0.6712 | 0.9506 | 1.1049 | 0.9720 | 1.0092 | 0.6823 | 0.6508 | 0.2856 |
| Ibrutinib (PCI-32765) | 0.6829 | 0.7056 | 0.6689 | 1.0015 | 1.0587 | 0.9663 | 1.0089 | 0.6823 | 0.6508 | 0.2856 |
| Taurolidine | 0.7065 | 0.6855 | 0.6653 | 1.0610 | 1.0081 | 0.9572 | 1.0087 | 0.6823 | 0.6508 | 0.2856 |
| Panaxatriol | 0.7231 | 0.6700 | 0.6640 | 1.1028 | 0.9690 | 0.9538 | 1.0085 | 0.6823 | 0.6508 | 0.2856 |
| Farnesol | 0.7238 | 0.6744 | 0.6573 | 1.1046 | 0.9801 | 0.9371 | 1.0073 | 0.6823 | 0.6508 | 0.2856 |
| Fingolimod | 0.6562 | 0.7249 | 0.6738 | 0.9342 | 1.1074 | 0.9785 | 1.0067 | 0.6823 | 0.6508 | 0.2856 |
| (-)-Verbenone | 0.6817 | 0.7057 | 0.6665 | 0.9985 | 1.0590 | 0.9601 | 1.0059 | 0.6823 | 0.6508 | 0.2856 |
| Etretinate | 0.6758 | 0.7124 | 0.6653 | 0.9836 | 1.0759 | 0.9571 | 1.0055 | 0.6823 | 0.6508 | 0.2856 |
| Glycocholic acid | 0.6883 | 0.6965 | 0.6628 | 1.0151 | 1.0358 | 0.9510 | 1.0006 | 0.6823 | 0.6508 | 0.2856 |
| Vitexin-2-O-rhaMnoside | 0.5896 | 0.7525 | 0.7043 | 0.7663 | 1.1770 | 1.0554 | 0.9996 | 0.6823 | 0.6508 | 0.2856 |
| Equol | 0.6540 | 0.7484 | 0.6428 | 0.9287 | 1.1666 | 0.9004 | 0.9986 | 0.6823 | 0.6508 | 0.2856 |
| Genistein (NPI 031L) | 0.6966 | 0.6863 | 0.6615 | 1.0360 | 1.0101 | 0.9476 | 0.9979 | 0.6823 | 0.6508 | 0.2856 |
| Ligustilide | 0.5892 | 0.7970 | 0.6574 | 0.7653 | 1.2891 | 0.9373 | 0.9972 | 0.6823 | 0.6508 | 0.2856 |
| Oleuropein | 0.6419 | 0.7168 | 0.6847 | 0.8982 | 1.0870 | 1.0061 | 0.9971 | 0.6823 | 0.6508 | 0.2856 |
| Octyl gallate | 0.6262 | 0.7243 | 0.6925 | 0.8586 | 1.1059 | 1.0256 | 0.9967 | 0.6823 | 0.6508 | 0.2856 |
| Isoquercitrin | 0.6904 | 0.6835 | 0.6689 | 1.0204 | 1.0030 | 0.9663 | 0.9966 | 0.6823 | 0.6508 | 0.2856 |
| Panobinostat (LBH589) | 0.6820 | 0.6995 | 0.6609 | 0.9992 | 1.0434 | 0.9461 | 0.9962 | 0.6823 | 0.6508 | 0.2856 |
| Tylosin | 0.6920 | 0.6827 | 0.6644 | 1.0245 | 1.0010 | 0.9549 | 0.9934 | 0.6823 | 0.6508 | 0.2856 |
| Apigenin (LY 080400) | 0.6281 | 0.7444 | 0.6658 | 0.8634 | 1.1565 | 0.9584 | 0.9928 | 0.6823 | 0.6508 | 0.2856 |
| Brucine sulfate heptahydrate | 0.6792 | 0.6776 | 0.6810 | 0.9922 | 0.9882 | 0.9968 | 0.9924 | 0.6823 | 0.6508 | 0.2856 |
| Dipotassium glycyrrhizinate | 0.6248 | 0.7594 | 0.6532 | 0.8551 | 1.1944 | 0.9266 | 0.9920 | 0.6823 | 0.6508 | 0.2856 |
| Fulvestrant (ICI-182780) | 0.6762 | 0.6971 | 0.6629 | 0.9846 | 1.0373 | 0.9512 | 0.9911 | 0.6823 | 0.6508 | 0.2856 |
| Marimastat (BB-2516) | 0.6879 | 0.6384 | 0.7058 | 1.0141 | 0.8893 | 1.0593 | 0.9876 | 0.6823 | 0.6508 | 0.2856 |
| Citronellal | 0.6424 | 0.6988 | 0.6850 | 0.8994 | 1.0416 | 1.0068 | 0.9826 | 0.6823 | 0.6508 | 0.2856 |
| Cladribine (RWJ 26251) | 0.6434 | 0.7279 | 0.6539 | 0.9019 | 1.1149 | 0.9284 | 0.9818 | 0.6823 | 0.6508 | 0.2856 |
| Abemaciclib (LY2835219) | 0.6855 | 0.6217 | 0.7175 | 1.0081 | 0.8472 | 1.0887 | 0.9813 | 0.6823 | 0.6508 | 0.2856 |
| Arbutin | 0.7116 | 0.6508 | 0.6601 | 1.0739 | 0.9206 | 0.9441 | 0.9795 | 0.6823 | 0.6508 | 0.2856 |
| Larotrectinib | 0.7020 | 0.6341 | 0.6848 | 1.0497 | 0.8785 | 1.0063 | 0.9782 | 0.6823 | 0.6508 | 0.2856 |
| 2-Deoxy-D-glucose (2-DG) | 0.6214 | 0.7134 | 0.6857 | 0.8465 | 1.0784 | 1.0086 | 0.9778 | 0.6823 | 0.6508 | 0.2856 |
| Apalutamide (ARN-509) | 0.7610 | 0.6273 | 0.6314 | 1.1984 | 0.8614 | 0.8718 | 0.9772 | 0.6823 | 0.6508 | 0.2856 |
| Folinic acid | 0.6634 | 0.6749 | 0.6790 | 0.9524 | 0.9813 | 0.9917 | 0.9751 | 0.6823 | 0.6508 | 0.2856 |
| Irinotecan hydrochloride | 0.6911 | 0.6563 | 0.6697 | 1.0222 | 0.9345 | 0.9682 | 0.9749 | 0.6823 | 0.6508 | 0.2856 |
| Catharanthine hemitartrate | 0.6742 | 0.6882 | 0.6528 | 0.9796 | 1.0149 | 0.9256 | 0.9734 | 0.6823 | 0.6508 | 0.2856 |
| L-3,4-Dihydroxyphenylalanine methyl ester hydrochloride | 0.6167 | 0.7294 | 0.6680 | 0.8346 | 1.1187 | 0.9640 | 0.9725 | 0.6823 | 0.6508 | 0.2856 |
| Megestrol | 0.6720 | 0.6744 | 0.6673 | 0.9740 | 0.9801 | 0.9622 | 0.9721 | 0.6823 | 0.6508 | 0.2856 |
| Evodiamine | 0.7162 | 0.6597 | 0.6375 | 1.0855 | 0.9430 | 0.8869 | 0.9718 | 0.6823 | 0.6508 | 0.2856 |
| D-Mannose | 0.6154 | 0.6812 | 0.7157 | 0.8314 | 0.9972 | 1.0842 | 0.9709 | 0.6823 | 0.6508 | 0.2856 |
| Dabrafenib Mesylate | 0.7193 | 0.6320 | 0.6584 | 1.0933 | 0.8732 | 0.9397 | 0.9687 | 0.6823 | 0.6508 | 0.2856 |
| Troxerutin | 0.6829 | 0.6348 | 0.6920 | 1.0015 | 0.8803 | 1.0244 | 0.9687 | 0.6823 | 0.6508 | 0.2856 |
| Neohesperidin | 0.7359 | 0.6284 | 0.6452 | 1.1351 | 0.8641 | 0.9064 | 0.9685 | 0.6823 | 0.6508 | 0.2856 |
| Icotinib (BPI-2009H) | 0.7114 | 0.6061 | 0.6894 | 1.0734 | 0.8079 | 1.0178 | 0.9664 | 0.6823 | 0.6508 | 0.2856 |
| Glasdegib (PF-04449913) | 0.6224 | 0.6674 | 0.7166 | 0.8490 | 0.9624 | 1.0864 | 0.9660 | 0.6823 | 0.6508 | 0.2856 |
| Doxorubicin (Adriamycin) HCl | 0.6371 | 0.7130 | 0.6549 | 0.8861 | 1.0774 | 0.9311 | 0.9648 | 0.6823 | 0.6508 | 0.2856 |
| Thymol | 0.6705 | 0.6817 | 0.6485 | 0.9703 | 0.9985 | 0.9147 | 0.9612 | 0.6823 | 0.6508 | 0.2856 |
| Lactobionic acid | 0.6826 | 0.6670 | 0.6505 | 1.0008 | 0.9614 | 0.9198 | 0.9607 | 0.6823 | 0.6508 | 0.2856 |
| Perifosine (KRX-0401) | 0.5822 | 0.7260 | 0.6011 | 0.8122 | 1.2059 | 0.8638 | 0.9606 | 0.6823 | 0.6508 | 0.2856 |
| Salidroside | 0.6363 | 0.6765 | 0.6871 | 0.8840 | 0.9854 | 1.0122 | 0.9605 | 0.6823 | 0.6508 | 0.2856 |
| Lathyrol | 0.7333 | 0.6282 | 0.6354 | 1.1286 | 0.8636 | 0.8817 | 0.9580 | 0.6823 | 0.6508 | 0.2856 |
| Cytarabine hydrochloride | 0.6494 | 0.7164 | 0.6304 | 0.9171 | 1.0860 | 0.8691 | 0.9574 | 0.6823 | 0.6508 | 0.2856 |
| Dexamethasone palmitate | 0.6701 | 0.6698 | 0.6559 | 0.9692 | 0.9685 | 0.9334 | 0.9571 | 0.6823 | 0.6508 | 0.2856 |
| Ridaforolimus (Deforolimus, MK-8669) | 0.5741 | 0.7345 | 0.6853 | 0.7272 | 1.1316 | 1.0076 | 0.9555 | 0.6823 | 0.6508 | 0.2856 |
| Bosutinib (SKI-606) | 0.6707 | 0.6669 | 0.6548 | 0.9708 | 0.9612 | 0.9307 | 0.9542 | 0.6823 | 0.6508 | 0.2856 |
| (-)-Epigallocatechin Gallate | 0.6875 | 0.6267 | 0.6768 | 1.0131 | 0.8598 | 0.9862 | 0.9531 | 0.6823 | 0.6508 | 0.2856 |
| p-Anisaldehyde | 0.7339 | 0.6369 | 0.6199 | 1.1301 | 0.8856 | 0.8428 | 0.9528 | 0.6823 | 0.6508 | 0.2856 |
| Amsacrine hydrochloride | 0.6357 | 0.6683 | 0.6864 | 0.8825 | 0.9647 | 1.0105 | 0.9526 | 0.6823 | 0.6508 | 0.2856 |
| Stevioside | 0.6226 | 0.6787 | 0.6891 | 0.8495 | 0.9909 | 1.0170 | 0.9525 | 0.6823 | 0.6508 | 0.2856 |
| Psoralen | 0.6073 | 0.6790 | 0.7017 | 0.8109 | 0.9917 | 1.0488 | 0.9505 | 0.6823 | 0.6508 | 0.2856 |
| Citronellyl acetate | 0.6477 | 0.6556 | 0.6845 | 0.9128 | 0.9327 | 1.0056 | 0.9504 | 0.6823 | 0.6508 | 0.2856 |
| Benzyl cinnamate | 0.6968 | 0.6212 | 0.6673 | 1.0366 | 0.8460 | 0.9623 | 0.9483 | 0.6823 | 0.6508 | 0.2856 |
| Regorafenib (BAY-734506) Monohydrate | 0.6633 | 0.6819 | 0.6393 | 0.9521 | 0.9990 | 0.8916 | 0.9476 | 0.6823 | 0.6508 | 0.2856 |
| Decitabine (NSC 127716) | 0.6658 | 0.6744 | 0.6438 | 0.9584 | 0.9801 | 0.9029 | 0.9471 | 0.6823 | 0.6508 | 0.2856 |
| Chlormadinone acetate | 0.6461 | 0.7005 | 0.6374 | 0.9087 | 1.0459 | 0.8867 | 0.9471 | 0.6823 | 0.6508 | 0.2856 |
| Coluracetam | 0.6990 | 0.6538 | 0.6309 | 1.0421 | 0.9282 | 0.8705 | 0.9469 | 0.6823 | 0.6508 | 0.2856 |
| Caryophyllene oxide | 0.7276 | 0.6366 | 0.6190 | 1.1142 | 0.8848 | 0.8405 | 0.9465 | 0.6823 | 0.6508 | 0.2856 |
| Clofarabine | 0.6004 | 0.7186 | 0.6641 | 0.7935 | 1.0915 | 0.9541 | 0.9464 | 0.6823 | 0.6508 | 0.2856 |
| Entrectinib (RXDX-101) | 0.6046 | 0.6758 | 0.7022 | 0.8041 | 0.9836 | 1.0502 | 0.9460 | 0.6823 | 0.6508 | 0.2856 |
| (-)Epicatechin | 0.6596 | 0.6742 | 0.6479 | 0.9428 | 0.9796 | 0.9134 | 0.9452 | 0.6823 | 0.6508 | 0.2856 |
| Ginsenoside Re | 0.6513 | 0.6815 | 0.6482 | 0.9219 | 0.9980 | 0.9140 | 0.9446 | 0.6823 | 0.6508 | 0.2856 |
| Phenprocoumon | 0.6956 | 0.6297 | 0.6553 | 1.0335 | 0.8674 | 0.9320 | 0.9443 | 0.6823 | 0.6508 | 0.2856 |
| Afatinib (BIBW2992) Dimaleate | 0.6185 | 0.6904 | 0.6713 | 0.8392 | 1.0204 | 0.9723 | 0.9440 | 0.6823 | 0.6508 | 0.2856 |
| Binimetinib (MEK162) | 0.6608 | 0.6131 | 0.7043 | 0.9458 | 0.8256 | 1.0554 | 0.9423 | 0.6823 | 0.6508 | 0.2856 |
| Dacomitinib (PF-00299804) | 0.6968 | 0.6246 | 0.6538 | 1.0366 | 0.8546 | 0.9281 | 0.9397 | 0.6823 | 0.6508 | 0.2856 |
| Lenvatinib (E7080) Mesylate | 0.7168 | 0.6370 | 0.6209 | 1.0870 | 0.8858 | 0.8453 | 0.9394 | 0.6823 | 0.6508 | 0.2856 |
| Squalene | 0.7017 | 0.6441 | 0.6273 | 1.0489 | 0.9037 | 0.8615 | 0.9380 | 0.6823 | 0.6508 | 0.2856 |
| Ginsenoside Rg3 | 0.6358 | 0.6909 | 0.6458 | 0.8828 | 1.0217 | 0.9079 | 0.9375 | 0.6823 | 0.6508 | 0.2856 |
| Sennoside B | 0.6347 | 0.6606 | 0.6754 | 0.8800 | 0.9453 | 0.9826 | 0.9360 | 0.6823 | 0.6508 | 0.2856 |
| Camostat Mesilate | 0.6976 | 0.5629 | 0.7093 | 1.0386 | 0.6990 | 1.0682 | 0.9353 | 0.6823 | 0.6508 | 0.2856 |
| Zanubrutinib (BGB-3111) | 0.6399 | 0.6549 | 0.6738 | 0.8931 | 0.9309 | 0.9786 | 0.9342 | 0.6823 | 0.6508 | 0.2856 |
| (+)-Catechin | 0.6211 | 0.6979 | 0.6479 | 0.8457 | 1.0393 | 0.9134 | 0.9328 | 0.6823 | 0.6508 | 0.2856 |
| Ginsenoside Rb1 | 0.6504 | 0.6473 | 0.6673 | 0.9196 | 0.9118 | 0.9623 | 0.9312 | 0.6823 | 0.6508 | 0.2856 |
| L-Fucose | 0.6427 | 0.6842 | 0.6369 | 0.9002 | 1.0048 | 0.8854 | 0.9301 | 0.6823 | 0.6508 | 0.2856 |
| (S)-10-Hydroxycamptothecin | 0.7197 | 0.6164 | 0.6274 | 1.0943 | 0.8339 | 0.8617 | 0.9300 | 0.6823 | 0.6508 | 0.2856 |
| Rhynchophylline | 0.6026 | 0.7208 | 0.6390 | 0.7991 | 1.0971 | 0.8909 | 0.9290 | 0.6823 | 0.6508 | 0.2856 |
| Arecoline HBr | 0.7313 | 0.6014 | 0.6265 | 1.1235 | 0.7961 | 0.8594 | 0.9263 | 0.6823 | 0.6508 | 0.2856 |
| Tivozanib (AV-951) | 0.6854 | 0.6346 | 0.6377 | 1.0078 | 0.8798 | 0.8877 | 0.9251 | 0.6823 | 0.6508 | 0.2856 |
| Polydatin | 0.6093 | 0.7211 | 0.6268 | 0.8160 | 1.0978 | 0.8601 | 0.9246 | 0.6823 | 0.6508 | 0.2856 |
| Pemetrexed | 0.6720 | 0.6495 | 0.6350 | 0.9740 | 0.9173 | 0.8809 | 0.9241 | 0.6823 | 0.6508 | 0.2856 |
| Imatinib (STI571) | 0.6861 | 0.6439 | 0.6258 | 1.0096 | 0.9032 | 0.8575 | 0.9234 | 0.6823 | 0.6508 | 0.2856 |
| Ceritinib (LDK378) | 0.6883 | 0.5612 | 0.7058 | 1.0151 | 0.6947 | 1.0593 | 0.9231 | 0.6823 | 0.6508 | 0.2856 |
| Fingolimod (FTY720) HCl | 0.6406 | 0.6855 | 0.6290 | 0.8949 | 1.0081 | 0.8657 | 0.9229 | 0.6823 | 0.6508 | 0.2856 |
| Scutellarin | 0.6168 | 0.6584 | 0.6782 | 0.8349 | 0.9398 | 0.9897 | 0.9215 | 0.6823 | 0.6508 | 0.2856 |
| Allylestrenol | 0.6716 | 0.6343 | 0.6469 | 0.9730 | 0.8790 | 0.9109 | 0.9210 | 0.6823 | 0.6508 | 0.2856 |
| Cyclophosphamide (NSC-26271) Monohydrate | 0.6256 | 0.6740 | 0.6531 | 0.8571 | 0.9791 | 0.9264 | 0.9209 | 0.6823 | 0.6508 | 0.2856 |
| Gallic acid | 0.6474 | 0.6371 | 0.6650 | 0.9120 | 0.8861 | 0.9564 | 0.9182 | 0.6823 | 0.6508 | 0.2856 |
| Embelin | 0.6951 | 0.5888 | 0.6648 | 1.0323 | 0.7643 | 0.9559 | 0.9175 | 0.6823 | 0.6508 | 0.2856 |
| Irinotecan (CPT-11) HCl Trihydrate | 0.6870 | 0.6134 | 0.6465 | 1.0118 | 0.8263 | 0.9097 | 0.9160 | 0.6823 | 0.6508 | 0.2856 |
| Larotrectinib (LOXO-101) sulfate | 0.6394 | 0.6932 | 0.6128 | 0.8919 | 1.0275 | 0.8248 | 0.9147 | 0.6823 | 0.6508 | 0.2856 |
| Dehydroandrographolide | 0.5718 | 0.7676 | 0.6057 | 0.7215 | 1.2150 | 0.8069 | 0.9145 | 0.6823 | 0.6508 | 0.2856 |
| Tamoxifen (ICI 46474) | 0.6508 | 0.6245 | 0.6648 | 0.9206 | 0.8543 | 0.9558 | 0.9102 | 0.6823 | 0.6508 | 0.2856 |
| Geraniol | 0.6876 | 0.6440 | 0.6068 | 1.0134 | 0.9035 | 0.8097 | 0.9089 | 0.6823 | 0.6508 | 0.2856 |
| Propyl gallate | 0.6519 | 0.6548 | 0.6305 | 0.9234 | 0.9307 | 0.8694 | 0.9078 | 0.6823 | 0.6508 | 0.2856 |
| Andrographolide | 0.6795 | 0.6232 | 0.6323 | 0.9929 | 0.8510 | 0.8741 | 0.9060 | 0.6823 | 0.6508 | 0.2856 |
| Temozolomide (CCRG 81045) | 0.6296 | 0.6405 | 0.6631 | 0.8672 | 0.8946 | 0.9515 | 0.9044 | 0.6823 | 0.6508 | 0.2856 |
| Daphnetin | 0.6155 | 0.6864 | 0.6306 | 0.8316 | 1.0103 | 0.8696 | 0.9039 | 0.6823 | 0.6508 | 0.2856 |
| Nelarabine | 0.6789 | 0.6276 | 0.6250 | 0.9914 | 0.8621 | 0.8554 | 0.9030 | 0.6823 | 0.6508 | 0.2856 |
| Cobimetinib (GDC-0973) | 0.5994 | 0.6776 | 0.6538 | 0.7910 | 0.9882 | 0.9281 | 0.9024 | 0.6823 | 0.6508 | 0.2856 |
| Cinnamyl acetate | 0.5806 | 0.6820 | 0.6673 | 0.7436 | 0.9992 | 0.9623 | 0.9017 | 0.6823 | 0.6508 | 0.2856 |
| Zoledronic acid (ZOL 446) | 0.6449 | 0.5694 | 0.6286 | 0.9838 | 0.7771 | 0.9393 | 0.9001 | 0.6823 | 0.6508 | 0.2856 |
| Rucaparib Camsylate | 0.6685 | 0.6210 | 0.6379 | 0.9652 | 0.8455 | 0.8882 | 0.8996 | 0.6823 | 0.6508 | 0.2856 |
| Linalyl Acetate | 0.7051 | 0.5642 | 0.6572 | 1.0575 | 0.7023 | 0.9368 | 0.8989 | 0.6823 | 0.6508 | 0.2856 |
| Siramesine HCl | 0.6125 | 0.6639 | 0.6425 | 0.8240 | 0.9536 | 0.8997 | 0.8925 | 0.6823 | 0.6508 | 0.2856 |
| alpha-Arbutin | 0.6723 | 0.6308 | 0.6150 | 0.9748 | 0.8702 | 0.8304 | 0.8918 | 0.6823 | 0.6508 | 0.2856 |
| Exemestane (FCE 24304) | 0.5439 | 0.7117 | 0.6617 | 0.6511 | 1.0741 | 0.9481 | 0.8911 | 0.6823 | 0.6508 | 0.2856 |
| Wedelolactone | 0.6419 | 0.6548 | 0.6204 | 0.8982 | 0.9307 | 0.8439 | 0.8909 | 0.6823 | 0.6508 | 0.2856 |
| Selinexor (KPT-330) | 0.6223 | 0.6183 | 0.6748 | 0.8488 | 0.8387 | 0.9811 | 0.8895 | 0.6823 | 0.6508 | 0.2856 |
| (-)-Epicatechin gallate | 0.6118 | 0.6491 | 0.6538 | 0.8223 | 0.9163 | 0.9281 | 0.8889 | 0.6823 | 0.6508 | 0.2856 |
| Cepharanthine | 0.6010 | 0.6700 | 0.6417 | 0.7951 | 0.9690 | 0.8977 | 0.8873 | 0.6823 | 0.6508 | 0.2856 |
| Harmine | 0.7586 | 0.5396 | 0.6119 | 1.1923 | 0.6403 | 0.8225 | 0.8850 | 0.6823 | 0.6508 | 0.2856 |
| Dicoumarol | 0.6537 | 0.6308 | 0.6250 | 0.9279 | 0.8702 | 0.8557 | 0.8846 | 0.6823 | 0.6508 | 0.2856 |
| Sorafenib (BAY 43-9006) | 0.5857 | 0.6183 | 0.7047 | 0.7565 | 0.8387 | 1.0565 | 0.8839 | 0.6823 | 0.6508 | 0.2856 |
| Nimorazole | 0.6972 | 0.5943 | 0.6164 | 1.0376 | 0.7782 | 0.8338 | 0.8832 | 0.6823 | 0.6508 | 0.2856 |
| Sotorasib (AMG510) | 0.5826 | 0.6986 | 0.6259 | 0.7487 | 1.0411 | 0.8579 | 0.8826 | 0.6823 | 0.6508 | 0.2856 |
| Ethyl gallate | 0.6975 | 0.5312 | 0.6738 | 1.0383 | 0.6191 | 0.9785 | 0.8787 | 0.6823 | 0.6508 | 0.2856 |
| Lenalidomide (CC-5013) | 0.5117 | 0.7193 | 0.6684 | 0.5700 | 1.0933 | 0.9650 | 0.8761 | 0.6823 | 0.6508 | 0.2856 |
| Dioscin | 0.6373 | 0.6130 | 0.6465 | 0.8866 | 0.8253 | 0.9098 | 0.8739 | 0.6823 | 0.6508 | 0.2856 |
| Venetoclax (ABT-199) | 0.6008 | 0.6321 | 0.6637 | 0.7946 | 0.8735 | 0.9532 | 0.8737 | 0.6823 | 0.6508 | 0.2856 |
| Fluorouracil (5-Fluorouracil, 5-FU) | 0.6466 | 0.6310 | 0.6175 | 0.9100 | 0.8707 | 0.8367 | 0.8724 | 0.6823 | 0.6508 | 0.2856 |
| Tucidinostat (Chidamide) | 0.5864 | 0.6706 | 0.6364 | 0.7583 | 0.9705 | 0.8842 | 0.8710 | 0.6823 | 0.6508 | 0.2856 |
| Xylitol | 0.6025 | 0.6383 | 0.6501 | 0.7988 | 0.8891 | 0.9189 | 0.8689 | 0.6823 | 0.6508 | 0.2856 |
| Cilengitide trifluoroacetate | 0.6329 | 0.5885 | 0.6693 | 0.8755 | 0.7635 | 0.9673 | 0.8688 | 0.6823 | 0.6508 | 0.2856 |
| Chloroambucil | 0.6110 | 0.6250 | 0.6533 | 0.8203 | 0.8556 | 0.9268 | 0.8676 | 0.6823 | 0.6508 | 0.2856 |
| Cyclophosphamide | 0.6853 | 0.5854 | 0.6175 | 1.0076 | 0.7557 | 0.8367 | 0.8667 | 0.6823 | 0.6508 | 0.2856 |
| Palbociclib | 0.6023 | 0.6506 | 0.6342 | 0.7983 | 0.9201 | 0.8788 | 0.8657 | 0.6823 | 0.6508 | 0.2856 |
| Neratinib (HKI-272) | 0.6259 | 0.6146 | 0.6459 | 0.8578 | 0.8293 | 0.9082 | 0.8651 | 0.6823 | 0.6508 | 0.2856 |
| Raloxifene HCl | 0.6332 | 0.6493 | 0.6034 | 0.8762 | 0.9168 | 0.8012 | 0.8647 | 0.6823 | 0.6508 | 0.2856 |
| Ginsenoside Rg1 | 0.6327 | 0.5764 | 0.6752 | 0.8750 | 0.7330 | 0.9821 | 0.8634 | 0.6823 | 0.6508 | 0.2856 |
| Cabozantinib (BMS-907351) | 0.6190 | 0.5994 | 0.6637 | 0.8404 | 0.7910 | 0.9531 | 0.8615 | 0.6823 | 0.6508 | 0.2856 |
| Hydroxyzine pamoate | 0.6238 | 0.6496 | 0.6077 | 0.8525 | 0.9176 | 0.8120 | 0.8607 | 0.6823 | 0.6508 | 0.2856 |
| Harmine hydrochloride | 0.6105 | 0.6639 | 0.6057 | 0.8190 | 0.9536 | 0.8069 | 0.8598 | 0.6823 | 0.6508 | 0.2856 |
| Formestane | 0.6480 | 0.5968 | 0.6315 | 0.9135 | 0.7845 | 0.8719 | 0.8567 | 0.6823 | 0.6508 | 0.2856 |
| Methenamine Hippurate | 0.5131 | 0.6913 | 0.6718 | 0.5735 | 1.0227 | 0.9736 | 0.8566 | 0.6823 | 0.6508 | 0.2856 |
| Sophoricoside | 0.6104 | 0.6165 | 0.6474 | 0.8188 | 0.8341 | 0.9120 | 0.8550 | 0.6823 | 0.6508 | 0.2856 |
| Coumarin | 0.5683 | 0.6675 | 0.6368 | 0.7126 | 0.9627 | 0.8854 | 0.8536 | 0.6823 | 0.6508 | 0.2856 |
| Veratric acid | 0.5941 | 0.6288 | 0.6474 | 0.7777 | 0.8651 | 0.9121 | 0.8516 | 0.6823 | 0.6508 | 0.2856 |
| Dacarbazine (NSC-45388) | 0.5658 | 0.6673 | 0.6350 | 0.7063 | 0.9622 | 0.8809 | 0.8498 | 0.6823 | 0.6508 | 0.2856 |
| Trametinib (GSK1120212) | 0.6387 | 0.6201 | 0.6083 | 0.8901 | 0.8432 | 0.8135 | 0.8489 | 0.6823 | 0.6508 | 0.2856 |
| Propranolol HCl | 0.6143 | 0.6441 | 0.6078 | 0.8286 | 0.9037 | 0.8123 | 0.8482 | 0.6823 | 0.6508 | 0.2856 |
| Quercitrin | 0.6284 | 0.5968 | 0.6383 | 0.8641 | 0.7845 | 0.8890 | 0.8459 | 0.6823 | 0.6508 | 0.2856 |
| Lactitol monohydrate | 0.6240 | 0.6269 | 0.6096 | 0.8530 | 0.8603 | 0.8166 | 0.8433 | 0.6823 | 0.6508 | 0.2856 |
| Protocatechuic acid | 0.6647 | 0.5557 | 0.6374 | 0.9556 | 0.6809 | 0.8868 | 0.8411 | 0.6823 | 0.6508 | 0.2856 |
| Temozolomide Acid | 0.5386 | 0.7351 | 0.5840 | 0.6378 | 1.1331 | 0.7523 | 0.8411 | 0.6823 | 0.6508 | 0.2856 |
| Rutin hydrate | 0.6197 | 0.6444 | 0.5927 | 0.8422 | 0.9045 | 0.7742 | 0.8403 | 0.6823 | 0.6508 | 0.2856 |
| (S)-crizotinib | 0.5731 | 0.6091 | 0.6738 | 0.7247 | 0.8155 | 0.9785 | 0.8396 | 0.6823 | 0.6508 | 0.2856 |
| Bendamustine (SDX105) | 0.6623 | 0.6078 | 0.5858 | 0.9496 | 0.8122 | 0.7568 | 0.8395 | 0.6823 | 0.6508 | 0.2856 |
| Ligustrazine hydrochloride | 0.5812 | 0.6308 | 0.6439 | 0.7451 | 0.8702 | 0.9032 | 0.8395 | 0.6823 | 0.6508 | 0.2856 |
| Eugenol | 0.6147 | 0.6325 | 0.6074 | 0.8296 | 0.8745 | 0.8113 | 0.8384 | 0.6823 | 0.6508 | 0.2856 |
| Bergenin | 0.5635 | 0.7021 | 0.5873 | 0.7005 | 1.0499 | 0.7606 | 0.8370 | 0.6823 | 0.6508 | 0.2856 |
| Etoposide (VP-16) | 0.5924 | 0.6331 | 0.6272 | 0.7734 | 0.8760 | 0.8610 | 0.8368 | 0.6823 | 0.6508 | 0.2856 |
| Lorlatinib (PF-6463922) | 0.5523 | 0.6109 | 0.6874 | 0.6723 | 0.8200 | 1.0129 | 0.8351 | 0.6823 | 0.6508 | 0.2856 |
| Menthol | 0.5161 | 0.7239 | 0.6059 | 0.5810 | 1.1049 | 0.8074 | 0.8311 | 0.6823 | 0.6508 | 0.2856 |
| Madecassoside | 0.6966 | 0.5419 | 0.6063 | 1.0360 | 0.6461 | 0.8084 | 0.8302 | 0.6823 | 0.6508 | 0.2856 |
| Estradiol Benzoate | 0.6302 | 0.6101 | 0.6038 | 0.8687 | 0.8180 | 0.8021 | 0.8296 | 0.6823 | 0.6508 | 0.2856 |
| Lapatinib (GW-572016) Ditosylate | 0.6279 | 0.5775 | 0.6348 | 0.8629 | 0.7358 | 0.8803 | 0.8263 | 0.6823 | 0.6508 | 0.2856 |
| Guaiacol | 0.6357 | 0.5952 | 0.6067 | 0.8825 | 0.7804 | 0.8095 | 0.8242 | 0.6823 | 0.6508 | 0.2856 |
| Sorbitol | 0.5982 | 0.6216 | 0.6174 | 0.7880 | 0.8470 | 0.8365 | 0.8238 | 0.6823 | 0.6508 | 0.2856 |
| Ribociclib (LEE011) | 0.5269 | 0.6294 | 0.6795 | 0.6083 | 0.8666 | 0.9930 | 0.8226 | 0.6823 | 0.6508 | 0.2856 |
| Erythritol | 0.6273 | 0.6086 | 0.5994 | 0.8614 | 0.8142 | 0.7909 | 0.8222 | 0.6823 | 0.6508 | 0.2856 |
| Talazoparib (BMN 673) | 0.6128 | 0.6155 | 0.6068 | 0.8248 | 0.8316 | 0.8098 | 0.8221 | 0.6823 | 0.6508 | 0.2856 |
| Ripretinib (DCC-2618) | 0.5806 | 0.6468 | 0.6059 | 0.7436 | 0.9105 | 0.8075 | 0.8205 | 0.6823 | 0.6508 | 0.2856 |
| Vanillic acid | 0.5788 | 0.6634 | 0.5893 | 0.7391 | 0.9524 | 0.7655 | 0.8190 | 0.6823 | 0.6508 | 0.2856 |
| Crizotinib hydrochloride | 0.6307 | 0.5985 | 0.6020 | 0.8699 | 0.7888 | 0.7976 | 0.8187 | 0.6823 | 0.6508 | 0.2856 |
| Nilotinib hydrochloride | 0.5757 | 0.6704 | 0.5848 | 0.7313 | 0.9700 | 0.7542 | 0.8185 | 0.6823 | 0.6508 | 0.2856 |
| Dasatinib (BMS-354825) | 0.5800 | 0.6339 | 0.6167 | 0.7421 | 0.8780 | 0.8348 | 0.8183 | 0.6823 | 0.6508 | 0.2856 |
| (−)-β-Pinene | 0.6966 | 0.5528 | 0.5809 | 1.0360 | 0.6736 | 0.7445 | 0.8180 | 0.6823 | 0.6508 | 0.2856 |
| Sonidegib (NVP-LDE225) | 0.6238 | 0.5913 | 0.6140 | 0.8525 | 0.7706 | 0.8278 | 0.8170 | 0.6823 | 0.6508 | 0.2856 |
| Sotorasib (AMG510) racemate | 0.5846 | 0.6364 | 0.6074 | 0.7537 | 0.8843 | 0.8113 | 0.8164 | 0.6823 | 0.6508 | 0.2856 |
| Ponatinib (AP24534) | 0.6264 | 0.5969 | 0.6032 | 0.8591 | 0.7847 | 0.8006 | 0.8148 | 0.6823 | 0.6508 | 0.2856 |
| CB-5339 | 0.5090 | 0.6931 | 0.6209 | 0.5631 | 1.0272 | 0.8453 | 0.8119 | 0.6823 | 0.6508 | 0.2856 |
| Abarelix Acetate | 0.5533 | 0.6874 | 0.5806 | 0.6748 | 1.0129 | 0.7436 | 0.8104 | 0.6823 | 0.6508 | 0.2856 |
| Ginkgolide C | 0.5807 | 0.6428 | 0.5974 | 0.7439 | 0.9004 | 0.7860 | 0.8101 | 0.6823 | 0.6508 | 0.2856 |
| Echinacoside | 0.6089 | 0.6130 | 0.5978 | 0.8150 | 0.8253 | 0.7871 | 0.8091 | 0.6823 | 0.6508 | 0.2856 |
| Triapine | 0.5034 | 0.6074 | 0.7074 | 0.5490 | 0.8112 | 1.0631 | 0.8078 | 0.6823 | 0.6508 | 0.2856 |
| Fumagillin | 0.5716 | 0.5863 | 0.6593 | 0.7209 | 0.7580 | 0.9420 | 0.8070 | 0.6823 | 0.6508 | 0.2856 |
| Tucatinib | 0.5820 | 0.6085 | 0.6237 | 0.7472 | 0.8140 | 0.8524 | 0.8045 | 0.6823 | 0.6508 | 0.2856 |
| Sunitinib (SU11248) | 0.5473 | 0.6153 | 0.6391 | 0.6597 | 0.8311 | 0.8911 | 0.7940 | 0.6823 | 0.6508 | 0.2856 |
| Paeoniflorin (NSC 178886) | 0.5989 | 0.6127 | 0.5889 | 0.7898 | 0.8246 | 0.7646 | 0.7930 | 0.6823 | 0.6508 | 0.2856 |
| Cabozantinib malate | 0.6079 | 0.5560 | 0.6349 | 0.8125 | 0.6816 | 0.8805 | 0.7915 | 0.6823 | 0.6508 | 0.2856 |
| Avapritinib (BLU-285) | 0.6836 | 0.5331 | 0.5821 | 1.0033 | 0.6239 | 0.7473 | 0.7915 | 0.6823 | 0.6508 | 0.2856 |
| Baicalin (NSC-661431) | 0.5619 | 0.6295 | 0.6061 | 0.6965 | 0.8669 | 0.8080 | 0.7905 | 0.6823 | 0.6508 | 0.2856 |
| Osimertinib (AZD9291) | 0.5550 | 0.5746 | 0.6657 | 0.6791 | 0.7285 | 0.9582 | 0.7886 | 0.6823 | 0.6508 | 0.2856 |
| Liquiritin | 0.6056 | 0.5777 | 0.6109 | 0.8067 | 0.7363 | 0.8201 | 0.7877 | 0.6823 | 0.6508 | 0.2856 |
| Encorafenib (LGX818) | 0.6461 | 0.5590 | 0.5873 | 0.9087 | 0.6892 | 0.7605 | 0.7862 | 0.6823 | 0.6508 | 0.2856 |
| Epirubicin (IMI 28) HCl | 0.5265 | 0.6608 | 0.6048 | 0.6073 | 0.9458 | 0.8048 | 0.7859 | 0.6823 | 0.6508 | 0.2856 |
| Ribociclib succinate | 0.5988 | 0.6047 | 0.5885 | 0.7895 | 0.8044 | 0.7635 | 0.7858 | 0.6823 | 0.6508 | 0.2856 |
| Asiatic Acid | 0.5039 | 0.6849 | 0.6013 | 0.5503 | 1.0066 | 0.7959 | 0.7843 | 0.6823 | 0.6508 | 0.2856 |
| Pomalidomide (CC-4047) | 0.6066 | 0.5804 | 0.6026 | 0.8092 | 0.7431 | 0.7991 | 0.7838 | 0.6823 | 0.6508 | 0.2856 |
| Nifurtimox | 0.6585 | 0.5427 | 0.5875 | 0.9400 | 0.6481 | 0.7610 | 0.7830 | 0.6823 | 0.6508 | 0.2856 |
| Ivosidenib (AG-120) | 0.5490 | 0.6143 | 0.6238 | 0.6640 | 0.8286 | 0.8524 | 0.7817 | 0.6823 | 0.6508 | 0.2856 |
| Anlotinib (AL3818) dihydrochloride | 0.5361 | 0.6641 | 0.5838 | 0.6315 | 0.9541 | 0.7517 | 0.7791 | 0.6823 | 0.6508 | 0.2856 |
| Ribociclib hydrochloride | 0.5814 | 0.6121 | 0.5890 | 0.7457 | 0.8230 | 0.7649 | 0.7779 | 0.6823 | 0.6508 | 0.2856 |
| Niraparib (MK-4827) tosylate | 0.5448 | 0.6214 | 0.6157 | 0.6534 | 0.8465 | 0.8322 | 0.7773 | 0.6823 | 0.6508 | 0.2856 |
| Notoginsenoside R1 | 0.5436 | 0.6668 | 0.5713 | 0.6504 | 0.9609 | 0.7203 | 0.7772 | 0.6823 | 0.6508 | 0.2856 |
| Brigatinib (AP26113) | 0.5711 | 0.6066 | 0.5982 | 0.7197 | 0.8092 | 0.7880 | 0.7723 | 0.6823 | 0.6508 | 0.2856 |
| Idelalisib | 0.6100 | 0.5609 | 0.6048 | 0.8177 | 0.6940 | 0.8047 | 0.7722 | 0.6823 | 0.6508 | 0.2856 |
| Cytarabine (U-19920A) | 0.5372 | 0.6158 | 0.5454 | 0.6889 | 0.9042 | 0.7114 | 0.7682 | 0.6823 | 0.6508 | 0.2856 |
| Camptothecin (NSC-100880) | 0.5996 | 0.5707 | 0.5963 | 0.7915 | 0.7187 | 0.7832 | 0.7645 | 0.6823 | 0.6508 | 0.2856 |
| Cabazitaxel (XRP6258) | 0.5693 | 0.5863 | 0.6093 | 0.7151 | 0.7580 | 0.8161 | 0.7631 | 0.6823 | 0.6508 | 0.2856 |
| Enasidenib (AG-221) | 0.5533 | 0.6349 | 0.5695 | 0.6748 | 0.8805 | 0.7157 | 0.7570 | 0.6823 | 0.6508 | 0.2856 |
| Tamibarotene | 0.5743 | 0.5828 | 0.5974 | 0.7278 | 0.7492 | 0.7861 | 0.7543 | 0.6823 | 0.6508 | 0.2856 |
| Berberine Sulfate | 0.5792 | 0.6155 | 0.5594 | 0.7401 | 0.8316 | 0.6901 | 0.7540 | 0.6823 | 0.6508 | 0.2856 |
| Pirarubicin (NSC-333054) | 0.5776 | 0.5702 | 0.6032 | 0.7361 | 0.7174 | 0.8006 | 0.7514 | 0.6823 | 0.6508 | 0.2856 |
| Rotenone (Barbasco) | 0.6392 | 0.5083 | 0.6012 | 0.8914 | 0.5614 | 0.7956 | 0.7495 | 0.6823 | 0.6508 | 0.2856 |
| Radotinib | 0.5493 | 0.5761 | 0.6179 | 0.6647 | 0.7323 | 0.8378 | 0.7449 | 0.6823 | 0.6508 | 0.2856 |
| Ceritinib dihydrochloride | 0.6096 | 0.5651 | 0.5684 | 0.8167 | 0.7046 | 0.7128 | 0.7447 | 0.6823 | 0.6508 | 0.2856 |
| Doramectin | 0.5787 | 0.6037 | 0.5597 | 0.7388 | 0.8019 | 0.6910 | 0.7439 | 0.6823 | 0.6508 | 0.2856 |
| Olmutinib (BI 1482694) | 0.5535 | 0.5783 | 0.6063 | 0.6753 | 0.7378 | 0.8085 | 0.7405 | 0.6823 | 0.6508 | 0.2856 |
| Oxaliplatin (NSC 266046) | 0.5722 | 0.5281 | 0.5671 | 0.7848 | 0.6640 | 0.7709 | 0.7399 | 0.6823 | 0.6508 | 0.2856 |
| Prucalopride Succinate | 0.5515 | 0.6085 | 0.5739 | 0.6703 | 0.8140 | 0.7267 | 0.7370 | 0.6823 | 0.6508 | 0.2856 |
| Darolutamide (ODM-201) | 0.5354 | 0.6063 | 0.5874 | 0.6297 | 0.8084 | 0.7609 | 0.7330 | 0.6823 | 0.6508 | 0.2856 |
| Cytisine | 0.5755 | 0.5820 | 0.5698 | 0.7308 | 0.7472 | 0.7165 | 0.7315 | 0.6823 | 0.6508 | 0.2856 |
| Mycophenolic acid | 0.5399 | 0.6722 | 0.5131 | 0.6410 | 0.9745 | 0.5734 | 0.7297 | 0.6823 | 0.6508 | 0.2856 |
| Saikosaponin A | 0.5596 | 0.6037 | 0.5616 | 0.6907 | 0.8019 | 0.6959 | 0.7295 | 0.6823 | 0.6508 | 0.2856 |
| 6-Aminonicotinamide | 0.5868 | 0.5693 | 0.5674 | 0.7593 | 0.7151 | 0.7103 | 0.7282 | 0.6823 | 0.6508 | 0.2856 |
| Sodium dichloroacetate (DCA) | 0.5295 | 0.5846 | 0.6058 | 0.6148 | 0.7537 | 0.8071 | 0.7252 | 0.6823 | 0.6508 | 0.2856 |
| Dianhydrogalactitol (VAL-083) | 0.6160 | 0.5273 | 0.5680 | 0.8329 | 0.6093 | 0.7120 | 0.7180 | 0.6823 | 0.6508 | 0.2856 |
| Abemaciclib mesylate (LY2835219) | 0.5963 | 0.4869 | 0.6273 | 0.7832 | 0.5074 | 0.8615 | 0.7174 | 0.6823 | 0.6508 | 0.2856 |
| Liarozole dihydrochloride | 0.6053 | 0.5351 | 0.5679 | 0.8059 | 0.6289 | 0.7117 | 0.7155 | 0.6823 | 0.6508 | 0.2856 |
| Letrozole (CGS 20267) | 0.5828 | 0.5476 | 0.5723 | 0.7492 | 0.6604 | 0.7227 | 0.7108 | 0.6823 | 0.6508 | 0.2856 |
| Fludarabine Phosphate | 0.5834 | 0.5610 | 0.5544 | 0.7507 | 0.6942 | 0.6775 | 0.7075 | 0.6823 | 0.6508 | 0.2856 |
| Abiraterone Acetate (CB7630) | 0.5681 | 0.5488 | 0.5795 | 0.7121 | 0.6635 | 0.7410 | 0.7055 | 0.6823 | 0.6508 | 0.2856 |
| Chlorogenic Acid | 0.5706 | 0.5369 | 0.5877 | 0.7184 | 0.6335 | 0.7616 | 0.7045 | 0.6823 | 0.6508 | 0.2856 |
| Apatinib (YN968D1) mesylate | 0.6256 | 0.5190 | 0.5504 | 0.8571 | 0.5884 | 0.6675 | 0.7043 | 0.6823 | 0.6508 | 0.2856 |
| Doxorubicin | 0.4865 | 0.6158 | 0.5873 | 0.5064 | 0.8324 | 0.7605 | 0.6998 | 0.6823 | 0.6508 | 0.2856 |
| Nerolidol | 0.5460 | 0.6080 | 0.5310 | 0.6564 | 0.8127 | 0.6187 | 0.6959 | 0.6823 | 0.6508 | 0.2856 |
| Gilteritinib (ASP2215) | 0.4950 | 0.5671 | 0.6203 | 0.5279 | 0.7096 | 0.8438 | 0.6937 | 0.6823 | 0.6508 | 0.2856 |
| Duvelisib (IPI-145) | 0.6289 | 0.5394 | 0.5138 | 0.8654 | 0.6398 | 0.5752 | 0.6935 | 0.6823 | 0.6508 | 0.2856 |
| Gentisic acid | 0.5287 | 0.5625 | 0.5860 | 0.6128 | 0.6980 | 0.7574 | 0.6894 | 0.6823 | 0.6508 | 0.2856 |
| Erdafitinib (JNJ-42756493) | 0.5716 | 0.5546 | 0.5468 | 0.7209 | 0.6781 | 0.6585 | 0.6858 | 0.6823 | 0.6508 | 0.2856 |
| Catalpol | 0.5833 | 0.5790 | 0.5019 | 0.7504 | 0.7396 | 0.5453 | 0.6785 | 0.6823 | 0.6508 | 0.2856 |
| Relugolix | 0.5065 | 0.5665 | 0.5890 | 0.5568 | 0.7081 | 0.7649 | 0.6766 | 0.6823 | 0.6508 | 0.2856 |
| Pralsetinib (BLU-667) | 0.5034 | 0.5831 | 0.5679 | 0.5490 | 0.7499 | 0.7117 | 0.6702 | 0.6823 | 0.6508 | 0.2856 |
| 6-Mercaptopurine (6-MP) Monohydrate | 0.5041 | 0.5686 | 0.5784 | 0.5508 | 0.7134 | 0.7380 | 0.6674 | 0.6823 | 0.6508 | 0.2856 |
| Raloxifene | 0.5183 | 0.5598 | 0.5727 | 0.5866 | 0.6912 | 0.7238 | 0.6672 | 0.6823 | 0.6508 | 0.2856 |
| p-Coumaric Acid | 0.5997 | 0.5353 | 0.5157 | 0.7918 | 0.6294 | 0.5801 | 0.6671 | 0.6823 | 0.6508 | 0.2856 |
| Cinchonine(LA40221) | 0.5214 | 0.5582 | 0.5675 | 0.5944 | 0.6872 | 0.7105 | 0.6640 | 0.6823 | 0.6508 | 0.2856 |
| Squalane | 0.5237 | 0.5549 | 0.5639 | 0.6002 | 0.6789 | 0.7015 | 0.6602 | 0.6823 | 0.6508 | 0.2856 |
| Pyrvinium | 0.5587 | 0.4957 | 0.5790 | 0.6884 | 0.5296 | 0.7396 | 0.6526 | 0.6823 | 0.6508 | 0.2856 |
| Tannic acid | 0.5677 | 0.4839 | 0.5764 | 0.7111 | 0.4999 | 0.7331 | 0.6480 | 0.6823 | 0.6508 | 0.2856 |
| Dasatinib hydrochloride | 0.5737 | 0.5184 | 0.5069 | 0.7262 | 0.5868 | 0.5579 | 0.6237 | 0.6823 | 0.6508 | 0.2856 |
| HMN-214 | 0.5439 | 0.5361 | 0.5175 | 0.6511 | 0.6315 | 0.5846 | 0.6224 | 0.6823 | 0.6508 | 0.2856 |
| Nilutamide | 0.5304 | 0.5605 | 0.5036 | 0.6171 | 0.6930 | 0.5495 | 0.6198 | 0.6823 | 0.6508 | 0.2856 |
| Acalabrutinib (ACP-196) | 0.5326 | 0.5557 | 0.5015 | 0.6226 | 0.6809 | 0.5443 | 0.6159 | 0.6823 | 0.6508 | 0.2856 |
| Meisoindigo | 0.5067 | 0.5390 | 0.4982 | 0.5573 | 0.6388 | 0.5360 | 0.5774 | 0.6823 | 0.6508 | 0.2856 |
| Sorbic acid | 0.5220 | 0.5086 | 0.4974 | 0.5959 | 0.5621 | 0.5339 | 0.5640 | 0.6823 | 0.6508 | 0.2856 |
| Topotecan | 0.4634 | 0.5638 | 0.4827 | 0.4482 | 0.7013 | 0.4969 | 0.5488 | 0.6823 | 0.6508 | 0.2856 |
| D-Ribose | 0.4582 | 0.5793 | 0.4648 | 0.4351 | 0.7404 | 0.4517 | 0.5424 | 0.6823 | 0.6508 | 0.2856 |
| Nimustine Hydrochloride | 0.5544 | 0.4470 | 0.4813 | 0.6776 | 0.4069 | 0.4933 | 0.5259 | 0.6823 | 0.6508 | 0.2856 |
| Caffeic Acid | 0.4778 | 0.5021 | 0.5028 | 0.4845 | 0.5458 | 0.5474 | 0.5259 | 0.6823 | 0.6508 | 0.2856 |
| Topotecan (NSC609699) HCl | 0.4952 | 0.4720 | 0.5014 | 0.5284 | 0.4699 | 0.5440 | 0.5141 | 0.6823 | 0.6508 | 0.2856 |
| Midostaurin (PKC412) | 0.4722 | 0.5200 | 0.4690 | 0.4704 | 0.5909 | 0.4624 | 0.5079 | 0.6823 | 0.6508 | 0.2856 |
| Methotrexate disodium | 0.5502 | 0.4141 | 0.4473 | 0.7245 | 0.3519 | 0.4429 | 0.5064 | 0.6823 | 0.6508 | 0.2856 |
| Docetaxel (RP56976) | 0.4572 | 0.4851 | 0.5031 | 0.4326 | 0.5029 | 0.5484 | 0.4946 | 0.6823 | 0.6508 | 0.2856 |
| Rubitecan | 0.4871 | 0.4894 | 0.4665 | 0.5079 | 0.5137 | 0.4561 | 0.4926 | 0.6823 | 0.6508 | 0.2856 |
| Daunorubicin (RP 13057) HCl | 0.4174 | 0.5056 | 0.5196 | 0.3322 | 0.5546 | 0.5899 | 0.4922 | 0.6823 | 0.6508 | 0.2856 |
| Carglumic Acid | 0.4687 | 0.5035 | 0.4575 | 0.4616 | 0.5493 | 0.4332 | 0.4814 | 0.6823 | 0.6508 | 0.2856 |
| Paclitaxel (NSC 125973) | 0.4976 | 0.4115 | 0.4691 | 0.5344 | 0.3174 | 0.4627 | 0.4382 | 0.6823 | 0.6508 | 0.2856 |
| Dasatinib Monohydrate | 0.4636 | 0.4892 | 0.4118 | 0.4487 | 0.5132 | 0.3181 | 0.4267 | 0.6823 | 0.6508 | 0.2856 |
| Mitoxantrone (NSC-301739) 2HCl | 0.4311 | 0.5039 | 0.4268 | 0.3668 | 0.5503 | 0.3560 | 0.4244 | 0.6823 | 0.6508 | 0.2856 |
| Hydroxy Camptothecine | 0.4398 | 0.4220 | 0.4837 | 0.3887 | 0.3438 | 0.4993 | 0.4106 | 0.6823 | 0.6508 | 0.2856 |
| Pixantrone Maleate | 0.4911 | 0.4356 | 0.3982 | 0.5180 | 0.3781 | 0.2839 | 0.3934 | 0.6823 | 0.6508 | 0.2856 |
| Vincristine (NSC-67574) sulfate | 0.4162 | 0.4397 | 0.4418 | 0.3292 | 0.3885 | 0.3936 | 0.3704 | 0.6823 | 0.6508 | 0.2856 |
| Vindesine sulfate | 0.4205 | 0.4208 | 0.4484 | 0.3401 | 0.3408 | 0.4103 | 0.3637 | 0.6823 | 0.6508 | 0.2856 |
| Romidepsin | 0.4564 | 0.4127 | 0.4097 | 0.4306 | 0.3204 | 0.3129 | 0.3546 | 0.6823 | 0.6508 | 0.2856 |
| Triptolide (PG490) | 0.4292 | 0.3904 | 0.4567 | 0.3620 | 0.2642 | 0.4314 | 0.3525 | 0.6823 | 0.6508 | 0.2856 |
| Pralatrexate (NSC 754230) | 0.4285 | 0.4148 | 0.4015 | 0.3602 | 0.3257 | 0.2922 | 0.3261 | 0.6823 | 0.6508 | 0.2856 |
| Ansamitocin p-3 (Maytansinol isobutyrate, NSC292222) | 0.3960 | 0.4218 | 0.4264 | 0.2783 | 0.3433 | 0.3548 | 0.3255 | 0.6823 | 0.6508 | 0.2856 |
| Octinoxate | 0.3851 | 0.4484 | 0.3984 | 0.2508 | 0.4104 | 0.2842 | 0.3152 | 0.6823 | 0.6508 | 0.2856 |
| Podophyllotoxin (Podofilox) | 0.4186 | 0.3830 | 0.3947 | 0.3353 | 0.2455 | 0.2750 | 0.2853 | 0.6823 | 0.6508 | 0.2856 |
| Triptonide | 0.3960 | 0.4013 | 0.3738 | 0.2783 | 0.2917 | 0.2223 | 0.2641 | 0.6823 | 0.6508 | 0.2856 |
| Vinblastine (NSC-49842) sulfate | 0.3881 | 0.3943 | 0.3684 | 0.2584 | 0.2740 | 0.2086 | 0.2470 | 0.6823 | 0.6508 | 0.2856 |
| Methotrexate (CL-14377) | 0.3924 | 0.3735 | 0.3679 | 0.2692 | 0.2216 | 0.2076 | 0.2328 | 0.6823 | 0.6508 | 0.2856 |
| Docetaxel Trihydrate | 0.3799 | 0.3930 | 0.3548 | 0.2377 | 0.2707 | 0.1745 | 0.2276 | 0.6823 | 0.6508 | 0.2856 |
| Saikosaponin D | 0.3671 | 0.4072 | 0.3510 | 0.2054 | 0.3065 | 0.1650 | 0.2257 | 0.6823 | 0.6508 | 0.2856 |
| Linalool | 0.3693 | 0.3841 | 0.3540 | 0.2110 | 0.2483 | 0.1725 | 0.2106 | 0.6823 | 0.6508 | 0.2856 |
| Combretastatin A4 | 0.3776 | 0.3756 | 0.3479 | 0.2319 | 0.2269 | 0.1571 | 0.2053 | 0.6823 | 0.6508 | 0.2856 |
| Homoharringtonine (CGX-635) | 0.3629 | 0.3386 | 0.3738 | 0.1949 | 0.1336 | 0.2222 | 0.1836 | 0.6823 | 0.6508 | 0.2856 |
| Picropodophyllin (PPP) | 0.3528 | 0.3841 | 0.3309 | 0.1694 | 0.2483 | 0.1143 | 0.1773 | 0.6823 | 0.6508 | 0.2856 |
| Harringtonine | 0.3559 | 0.3432 | 0.3548 | 0.1772 | 0.1452 | 0.1743 | 0.1656 | 0.6823 | 0.6508 | 0.2856 |
| Monomethyl auristatin E (MMAE) | 0.3299 | 0.3595 | 0.3375 | 0.1117 | 0.1863 | 0.1309 | 0.1429 | 0.6823 | 0.6508 | 0.2856 |
| Axitinib (AG 013736) | 0.3150 | 0.3553 | 0.3417 | 0.0741 | 0.1758 | 0.1413 | 0.1304 | 0.6823 | 0.6508 | 0.2856 |
| Exatecan Mesylate | 0.3204 | 0.3623 | 0.3159 | 0.0877 | 0.1933 | 0.0764 | 0.1191 | 0.6823 | 0.6508 | 0.2856 |
| Vinorelbine ditartrate (KW-2307) | 0.3083 | 0.3674 | 0.3136 | 0.0572 | 0.2062 | 0.0705 | 0.1113 | 0.6823 | 0.6508 | 0.2856 |
| Ixazomib Citrate (MLN9708) Analogue | 0.3036 | 0.3284 | 0.3063 | 0.0454 | 0.1079 | 0.0521 | 0.0684 | 0.6823 | 0.6508 | 0.2856 |
| Afatinib (BIBW2992) | 0.3156 | 0.2943 | 0.3169 | 0.0756 | 0.0219 | 0.0790 | 0.0589 | 0.6823 | 0.6508 | 0.2856 |
| Protopine | 0.3642 | 0.2863 | 0.2689 | 0.1981 | 0.0018 | -0.0420 | 0.0526 | 0.6823 | 0.6508 | 0.2856 |
| Carfilzomib (PR-171) | 0.3036 | 0.2997 | 0.3104 | 0.0454 | 0.0355 | 0.0625 | 0.0478 | 0.6823 | 0.6508 | 0.2856 |
| Selumetinib (AZD6244) | 0.3000 | 0.3223 | 0.2900 | 0.0363 | 0.0926 | 0.0112 | 0.0467 | 0.6823 | 0.6508 | 0.2856 |
| Bortezomib (PS-341) | 0.3099 | 0.3116 | 0.2905 | 0.0613 | 0.0655 | 0.0125 | 0.0464 | 0.6823 | 0.6508 | 0.2856 |
| Ixazomib (MLN2238) | 0.3022 | 0.3109 | 0.2959 | 0.0418 | 0.0638 | 0.0259 | 0.0439 | 0.6823 | 0.6508 | 0.2856 |
| Ixabepilone (BMS-247550) | 0.3143 | 0.2949 | 0.2983 | 0.0723 | 0.0234 | 0.0320 | 0.0426 | 0.6823 | 0.6508 | 0.2856 |
| Staurosporine (AM-2282) | 0.3089 | 0.3065 | 0.2858 | 0.0587 | 0.0527 | 0.0006 | 0.0373 | 0.6823 | 0.6508 | 0.2856 |
| Halofuginone | 0.2890 | 0.3010 | 0.2837 | 0.0086 | 0.0388 | -0.0049 | 0.0142 | 0.6823 | 0.6508 | 0.2856 |
| Ixazomib Citrate (MLN9708) | 0.2861 | 0.2882 | 0.2843 | 0.0013 | 0.0066 | -0.0032 | 0.0015 | 0.6823 | 0.6508 | 0.2856 |

Table S7. Hydrogen bond interactions at the interface between ABHD10 and ACSL4

| **ABHD10 interface residue(s)** | | | **ACSL4 interface residue(s)** | | | **ABHD10 - ACSL4 interface residue pair(s)** | | | |
| --- | --- | --- | --- | --- | --- | --- | --- | --- | --- |
|  |  |  |  |  |  | **ABHD10** |  | **ACSL4** |  |
| Amino Acid Name | Position/Chain Number | Hydrogen Bonds(Å) | Amino Acid Name | Position/Chain Number | Hydrogen Bonds(Å) | Position/  Chain Number |  | Position/Chain Number | Hydrogen Bonds(Å) |
| ARG | 41A | 2.344 | MET | 1A | 2.653 | 41A | - | 22A | 3.415 |
| ALA | 42A | 3.646 | LYS | 2A | 4.724 | 41A | - | 26A | 2.344 |
| PRO | 43A | 3.022 | LEU | 3A | 3.677 | 42A | - | 22A | 3.646 |
| TRP | 45A | 1.540 | LEU | 5A | 4.698 | 42A | - | 26A | 3.866 |
| LYS | 74A | 4.883 | THR | 9A | 4.884 | 43A | - | 21A | 4.961 |
| PHE | 99A | 3.132 | LEU | 12A | 3.963 | 43A | - | 22A | 3.222 |
| SER | 102A | 4.378 | LEU | 13A | 2.419 | 43A | - | 25A | 3.022 |
| LEU | 103A | 3.346 | PRO | 14A | 4.248 | 43A | - | 26A | 3.213 |
| ASP | 270A | 4.070 | HIS | 16A | 2.783 | 45A | - | 21A | 1.54 |
| HIS | 276A | 2.653 | LEU | 17A | 4.005 | 45A | - | 22A | 4.701 |
| SER | 277A | 3.561 | ILE | 19A | 3.475 | 45A | - | 25A | 3.222 |
| ASP | 278A | 3.207 | THR | 20A | 2.957 | 74A | - | 28A | 4.883 |
| GLU | 283A | 3.677 | ILE | 21A | 1.540 | 99A | - | 24A | 3.132 |
| LYS | 284A | 2.419 | TYR | 22A | 3.222 | 102A | - | 24A | 4.647 |
| ALA | 285A | 4.389 | SER | 23A | 3.105 | 102A | - | 25A | 4.378 |
| ASP | 286A | 4.755 | ALA | 24A | 3.132 | 103A | - | 24A | 3.346 |
| GLN | 288A | 2.966 | LEU | 25A | 3.022 | 103A | - | 25A | 4.675 |
| LEU | 289A | 3.988 | ILE | 26A | 2.344 | 103A | - | 28A | 3.698 |
| VAL | 291A | 4.522 | PHE | 27A | 2.367 | 270A | - | 44A | 4.07 |
| TYR | 292A | 2.957 | ILE | 28A | 3.136 | 276A | - | 1A | 2.653 |
| ASP | 295A | 2.934 | TYR | 31A | 3.007 | 276A | - | 2A | 4.724 |
| ASP | 296A | 2.594 | PHE | 32A | 3.284 | 277A | - | 1A | 3.561 |
| ASP | 299A | 2.367 | LYS | 37A | 2.659 | 278A | - | 1A | 3.207 |
| LYS | 300A | 3.122 | LYS | 44A | 4.070 | 283A | - | 1A | 3.697 |
| SER | 302A | 3.440 | TYR | 406A | 3.317 | 283A | - | 3A | 3.677 |
| THR | 303A | 2.733 | GLN | 410A | 4.411 | 283A | - | 5A | 4.698 |
| ILE | 304A | 2.659 | LYS | 413A | 3.617 | 284A | - | 9A | 4.884 |
| ASN | 306A | 4.606 | TYR | 415A | 2.854 | 284A | - | 12A | 3.963 |
|  |  |  |  |  |  | 284A | - | 13A | 2.419 |
|  |  |  |  |  |  | 284A | - | 14A | 4.248 |
|  |  |  |  |  |  | 284A | - | 16A | 2.783 |
|  |  |  |  |  |  | 284A | - | 17A | 4.005 |
|  |  |  |  |  |  | 285A | - | 1A | 4.389 |
|  |  |  |  |  |  | 286A | - | 1A | 4.755 |
|  |  |  |  |  |  | 288A | - | 16A | 2.966 |
|  |  |  |  |  |  | 288A | - | 19A | 4.379 |
|  |  |  |  |  |  | 288A | - | 20A | 3.069 |
|  |  |  |  |  |  | 288A | - | 406A | 4.075 |
|  |  |  |  |  |  | 289A | - | 413A | 3.988 |
|  |  |  |  |  |  | 291A | - | 20A | 4.522 |
|  |  |  |  |  |  | 292A | - | 19A | 3.475 |
|  |  |  |  |  |  | 292A | - | 20A | 2.957 |
|  |  |  |  |  |  | 292A | - | 23A | 3.105 |
|  |  |  |  |  |  | 292A | - | 24A | 4.762 |
|  |  |  |  |  |  | 292A | - | 406A | 3.317 |
|  |  |  |  |  |  | 292A | - | 410A | 4.411 |
|  |  |  |  |  |  | 292A | - | 413A | 4.302 |
|  |  |  |  |  |  | 295A | - | 23A | 3.567 |
|  |  |  |  |  |  | 295A | - | 24A | 3.667 |
|  |  |  |  |  |  | 295A | - | 27A | 2.934 |
|  |  |  |  |  |  | 295A | - | 28A | 3.93 |
|  |  |  |  |  |  | 296A | - | 27A | 2.594 |
|  |  |  |  |  |  | 296A | - | 31A | 4.664 |
|  |  |  |  |  |  | 296A | - | 413A | 3.617 |
|  |  |  |  |  |  | 296A | - | 415A | 2.854 |
|  |  |  |  |  |  | 299A | - | 27A | 2.367 |
|  |  |  |  |  |  | 299A | - | 28A | 3.136 |
|  |  |  |  |  |  | 299A | - | 31A | 3.007 |
|  |  |  |  |  |  | 299A | - | 32A | 4.182 |
|  |  |  |  |  |  | 300A | - | 31A | 3.122 |
|  |  |  |  |  |  | 300A | - | 37A | 3.997 |
|  |  |  |  |  |  | 302A | - | 32A | 3.44 |
|  |  |  |  |  |  | 303A | - | 31A | 3.182 |
|  |  |  |  |  |  | 303A | - | 32A | 3.284 |
|  |  |  |  |  |  | 303A | - | 37A | 2.733 |
|  |  |  |  |  |  | 304A | - | 37A | 2.659 |
|  |  |  |  |  |  | 306A | - | 32A | 4.606 |
